# Cerebrospinal fluid proteomics define the natural history of autosomal dominant Alzheimer’s disease

**DOI:** 10.1038/s41591-023-02476-4

**Published:** 2023-08-07

**Authors:** Erik C. B. Johnson, Shijia Bian, Rafi U. Haque, E. Kathleen Carter, Caroline M. Watson, Brian A. Gordon, Lingyan Ping, Duc M. Duong, Michael P. Epstein, Eric McDade, Nicolas R. Barthélemy, Celeste M. Karch, Chengjie Xiong, Carlos Cruchaga, Richard J. Perrin, Aliza P. Wingo, Thomas S. Wingo, Jasmeer P. Chhatwal, Gregory S. Day, James M. Noble, Sarah B. Berman, Ralph Martins, Neill R. Graff-Radford, Peter R. Schofield, Takeshi Ikeuchi, Hiroshi Mori, Johannes Levin, Martin Farlow, James J. Lah, Christian Haass, Mathias Jucker, John C. Morris, Tammie L. S. Benzinger, Blaine R. Roberts, Randall J. Bateman, Anne M. Fagan, Nicholas T. Seyfried, Allan I. Levey, the Dominantly Inherited Alzheimer Network, Jonathan Vöglein, Ricardo Allegri, Patricio Chrem Mendez, Ezequiel Surace, Sarah B. Berman, Snezana Ikonomovic, Neelesh Nadkarni, Francisco Lopera, Laura Ramirez, David Aguillon, Yudy Leon, Claudia Ramos, Diana Alzate, Ana Baena, Natalia Londono, Sonia Moreno, Christoph Laske, Elke Kuder-Buletta, Susanne Graber-Sultan, Oliver Preische, Anna Hofmann, Kensaku Kasuga, Yoshiki Niimi, Kenji Ishii, Michio Senda, Raquel Sanchez-Valle, Pedro Rosa-Neto, Nick Fox, Dave Cash, Jae-Hong Lee, Jee Hoon Roh, Meghan Riddle, William Menard, Courtney Bodge, Mustafa Surti, Leonel Tadao Takada, V. J. Sanchez-Gonzalez, Maribel Orozco-Barajas, Alison Goate, Alan Renton, Bianca Esposito, Jacob Marsh, Carlos Cruchaga, Victoria Fernandez, Gina Jerome, Elizabeth Herries, Jorge Llibre-Guerra, William Brooks, Jacob Bechara, Jason Hassenstab, Erin Franklin, Allison Chen, Charles Chen, Shaney Flores, Nelly Friedrichsen, Nancy Hantler, Russ Hornbeck, Steve Jarman, Sarah Keefe, Deborah Koudelis, Parinaz Massoumzadeh, Austin McCullough, Nicole McKay, Joyce Nicklaus, Christine Pulizos, Qing Wang, Sheetal Mishall, Edita Sabaredzovic, Emily Deng, Madison Candela, Hunter Smith, Diana Hobbs, Jalen Scott, Peter Wang, Xiong Xu, Yan Li, Emily Gremminger, Yinjiao Ma, Ryan Bui, Ruijin Lu, Ana Luisa Sosa Ortiz, Alisha Daniels, Laura Courtney, Charlene Supnet-Bell, Jinbin Xu, John Ringman

**Affiliations:** 1 Goizueta Alzheimer’s Disease Research Center, Emory University School of Medicine, Atlanta, GA USA; 2 Department of Neurology, Emory University School of Medicine, Atlanta, GA USA; 3 Department of Biostatistics and Bioinformatics, Rollins School of Public Health, Emory University, Atlanta, GA USA; 4 Department of Biochemistry, Emory University School of Medicine, Atlanta, GA USA; 5 Mallinckrodt Institute of Radiology, Washington University in St Louis, St Louis, MO USA; 6 Department of Human Genetics, Emory University School of Medicine, Atlanta, GA USA; 7 Department of Neurology, Washington University in St Louis, St Louis, MO USA; 8 Department of Psychiatry, Washington University in St Louis, St Louis, MO USA; 9 Division of Biostatistics, Washington University in St Louis, St Louis, MO USA; 10 Department of Pathology and Immunology, Washington University in St Louis, St Louis, MO USA; 11 Department of Psychiatry, Emory University School of Medicine, Atlanta, GA USA; 12 Division of Mental Health, Atlanta VA Medical Center, Atlanta, GA USA; 13 Massachusetts General and Brigham & Women’s Hospitals, Harvard Medical School, Boston, MA USA; 14 Department of Neurology, Mayo Clinic, Jacksonville, FL USA; 15 Department of Neurology, Taub Institute for Research on Alzheimer’s Disease and the Aging Brain, and GH Sergievsky Center, Columbia University Irving Medical Center, New York, NY USA; 16 Departments of Neurology and Clinical and Translational Science, Pittsburgh Institute for Neurodegenerative Diseases, University of Pittsburgh, Pittsburgh, PA USA; 17 Edith Cowan University, Perth, Western Australia Australia; 18 Neuroscience Research Australia, Sydney, New South Wales Australia; 19 School of Biomedical Sciences, University of New South Wales, Sydney, New South Wales Australia; 20 Department of Molecular Genetics, Brain Research Institute, Niigata University, Niigata, Japan; 21 Osaka Metropolitan University Medical School, Nagaoka Sutoku University, Nagaoka, Japan; 22 Department of Neurology, Ludwig-Maximilians-Universität München, Munich, Germany; 23 Indiana University, Bloomington, IN USA; 24 German Center for Neurodegenerative Diseases (DZNE), Munich, Germany; 25 Metabolic Biochemistry, Biomedical Center (BMC), Ludwig-Maximilians University, Munich, Germany; 26 Munich Cluster for Systems Neurology (SyNergy), Munich, Germany; 27 Department of Cellular Neurology, Hertie Institute for Clinical Brain Research, University of Tübingen, Tübingen, Germany; 28 German Center for Neurodegenerative Diseases (DZNE), Tübingen, Germany; 29 Department of Cognitive Neurology, Institute for Neurological Research Fleni, Buenos Aires, Argentina; 30 Department of Molecular Biology and Neuropathology, Institute for Neurological Research Fleni, Buenos Aires, Argentina; 31 Department of Neurology, University of Pittsburgh, Pittsburgh, PA USA; 32 Clinical and Translational Science, Pittsburgh Institute for Neurodegenerative Diseases, University of Pittsburgh, Pittsburgh, PA USA; 33 Department of Geriatric Medicine, University of Pittsburgh, Pittsburgh, PA USA; 34 Grupo de Neurociencias de Antioquia (GNA), Universidad de Antioquia, Medellín, Colombia; 35 Hertie Institute for Clinical Brain Research, University of Tübingen, Tübingen, Germany; 36 Department of Psychiatry and Psychotherapy, University of Tübingen, Tübingen, Germany; 37 Brain Research Institute, Niigata University, Niigata, Japan; 38 The University of Tokyo Hospital Unit for Early and Exploratory Clinical Development, Tokyo, Japan; 39 Tokyo Metropolitan Institute of Gerontology, Tokyo, Japan; 40 Kobe City Medical Center General Hospital, Tokyo, Japan; 41 Alzheimer’s Disease and Other Cognitive Disorders Unit, Neurology Service, Hospital Clinic de Barcelona, Barcelona, Spain; 42 McGill University, Montreal, Quebec Canada; 43 Dementia Research Centre, UCL Queen Square Institute of Neurology, London, UK; 44 UK Dementia Research Institute at UCL, London, UK; 45 Korea University College of Medicine, Seoul, South Korea; 46 Butler Hospital, Warren Alpert School of Medicine, Brown University, Providence, RI USA; 47 Hospital das Clinicas, University of São Paulo School of Medicine, São Paulo, Brazil; 48 Doctorado en Biociencias & Departamento de Clinicas, Centro Universitario de Los Altos, UDG, Tepatitlán de Morelos, Mexico; 49 Department of Genetics and Genomic Sciences, Icahn School of Medicine at Mount Sinai, New York, NY USA; 50 Nash Family Department of Neuroscience, Icahn School of Medicine at Mount Sinai, New York, NY USA; 51 Ronald M. Loeb Center for Alzheimer’s Disease, Icahn School of Medicine at Mount Sinai, New York, NY USA; 52 NeuroGenomics and Informatics Center, Washington University School of Medicine, St Louis, MO USA; 53 School of Medical Sciences, University of New South Wales, Sydney, New South Wales Australia; 54 Department of Psychological and Brain Sciences, Washington University in St Louis, St Louis, MO USA; 55 Mexico City, Mexico; 56 Washington University School of Medicine in St Louis, St Louis, MO USA; 57 Department of Radiology, Washington University in St Louis, St Louis, MO USA; 58 Department of Neurology, Keck School of Medicine, University of Southern California, Los Angeles, CA USA

**Keywords:** Alzheimer's disease, Alzheimer's disease, Diagnostic markers, Alzheimer's disease

## Abstract

Alzheimer’s disease (AD) pathology develops many years before the onset of cognitive symptoms. Two pathological processes—aggregation of the amyloid-β (Aβ) peptide into plaques and the microtubule protein tau into neurofibrillary tangles (NFTs)—are hallmarks of the disease. However, other pathological brain processes are thought to be key disease mediators of Aβ plaque and NFT pathology. How these additional pathologies evolve over the course of the disease is currently unknown. Here we show that proteomic measurements in autosomal dominant AD cerebrospinal fluid (CSF) linked to brain protein coexpression can be used to characterize the evolution of AD pathology over a timescale spanning six decades. SMOC1 and SPON1 proteins associated with Aβ plaques were elevated in AD CSF nearly 30 years before the onset of symptoms, followed by changes in synaptic proteins, metabolic proteins, axonal proteins, inflammatory proteins and finally decreases in neurosecretory proteins. The proteome discriminated mutation carriers from noncarriers before symptom onset as well or better than Aβ and tau measures. Our results highlight the multifaceted landscape of AD pathophysiology and its temporal evolution. Such knowledge will be critical for developing precision therapeutic interventions and biomarkers for AD beyond those associated with Aβ and tau.

## Abstract (web summary)

Proteomic analysis of cerebrospinal fluid from individuals with autosomal dominant Alzheimer’s disease reveals how this complex and chronic disease evolves over many decades.

## Main

AD is a devastating neurodegenerative disease with increasing prevalence in aging societies^1^. AD is currently defined at a research level by the presence of high levels of aggregated Aβ peptide and tau NFTs in the brain, either in the presence or absence of cognitive impairment^2^. Assessment of Aβ plaque and NFT neuropathological burden can be performed by positron emission tomography (PET) imaging using radioactive tracers that bind to plaques and tangles, or by molecular protein biomarkers in CSF, and more recently in blood, that are currently available at either a clinical or research level^3–6^. However, it is widely appreciated that AD is a complex brain disorder with multiple pathological alterations that occur during the prodromal stage of the disease in addition to Aβ and tau dyshomeostasis, many of which are not readily apparent by neuropathological examination^7,8^. These other pathological processes may mechanistically link Aβ and tau pathology and provide promising therapeutic targets for AD other than Aβ and tau. Although the landscape of AD pathophysiology has been extensively characterized through multiomic studies on post-mortem brain tissue, such as those conducted through the Accelerating Medicines Partnership for Alzheimer’s Disease consortium^8–10^, limitations inherent in the study of molecular changes in brain tissue during life necessitate the development of biomarkers that can reflect the sequencing of these pathological changes over the course of the disease.

A key challenge to the study of AD prodromal changes is capturing these changes over the course of many years when people are otherwise relatively young and healthy. Another challenge is characterizing these changes in those who may never develop symptoms during their lifetimes despite the presence of Aβ plaque and NFT neuropathology. One approach to address these challenges is to study individuals who carry an autosomal dominantly inherited AD (ADAD) mutation in the *amyloid precursor protein* (*APP*), *presenilin 1* (*PSEN1*) or *presenilin 2* (*PSEN2*) gene that leads to increased relative production of the Aβ42 peptide throughout life and early brain Aβ plaque deposition^11,12^. ADAD mutations display nearly 100% disease penetrance, and the age of symptomatic onset is highly predictable based on the nature of the mutation and the family pedigree. The Dominantly Inherited Alzheimer Network (DIAN) observational study is a multisite worldwide effort to enroll and study individuals who carry ADAD mutations to increase understanding of the natural history of AD^11,13,14^. The DIAN observational study examines ADAD mutation carriers and their noncarrier family members using multiple assessments including imaging, cognitive, CSF and plasma measures, among others. Because of the relatively precise estimated year of disease onset (EYO) in ADAD mutation carriers, cross-sectional study assessments can provide highly valuable information on AD biomarker changes within a longitudinal framework.

Previous proteomic studies of sporadic AD CSF have revealed multiple proteins that are altered in later stages of the disease when individuals are cognitively impaired, and these proteins have been validated in multiple cohorts^9,15–17^. Based on these findings in late-onset AD (LOAD), we created a panel of 59 proteins and measured their CSF levels cross-sectionally in 286 ADAD mutation carriers and 184 noncarriers across the EYO continuum using a targeted quantitative mass spectrometry (MS) method called selected reaction monitoring mass spectrometry (SRM-MS)^18,19^. We used a recent large consensus protein coexpression analysis of AD brain in which 44 coexpression modules were generated from more than 8,600 proteins for biological interpretation of each biomarker^8^. By relating these proteins back to the AD brain coexpression modules with which they are associated, we were able to link these protein changes to multiple different AD brain pathological processes and estimate when and how these biomarkers change over the course of the disease. We also incorporated MS-based and enzyme-linked immunosorbent assay (ELISA) affinity measures of other high-value biomarker targets—such as Aβ and tau species—and different imaging and cognitive measures acquired in DIAN in the analysis to serve as benchmarks for the proteomic changes observed.

## Results

### Proteomics identifies early elevations in SMOC1 and the matrisome with subsequent cascading pathological changes

**Fig. 1:**
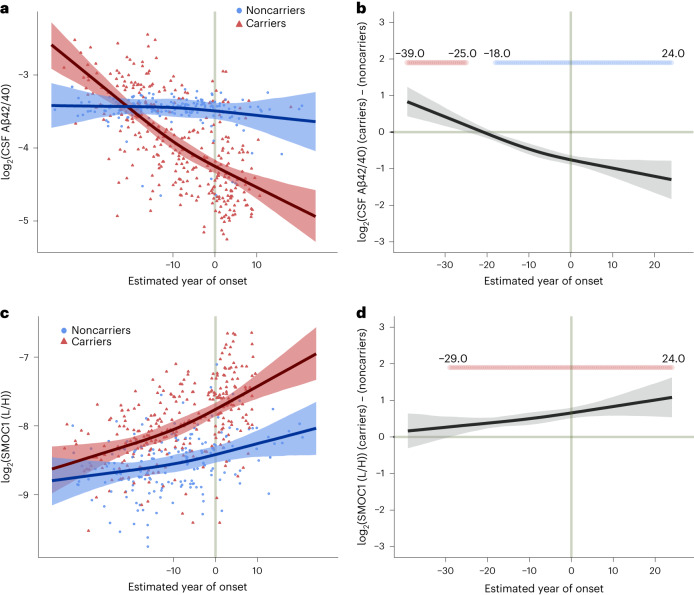
Aβ42/40 ratio and SMOC1 level in CSF by EYO in ADAD. **a**,**b**, The ratio of CSF Aβ42 to Aβ40 peptide as a measure of Aβ brain deposition (**a**) in ADAD mutation carriers and noncarriers and (**b**) the difference between carriers and noncarriers, by EYO. One outlier was removed from **a** for visualization purposes. **c**,**d**, CSF level of SMOC1—an Aβ plaque-associated protein—(**c**) in mutation carriers and noncarriers and (**d**) the difference between carriers and noncarriers, by EYO. One outlier was removed from **c** for visualization purposes. EYO labels outside the range of –10 to 10 in **a** and **c** are removed to maintain research participant confidentiality. Periods of significant difference between carriers and noncarriers are highlighted in **b** and **d** (red indicates significantly increased levels in carriers, blue indicates significantly decreased levels in carriers). Lines represent the median of the posterior estimates at each EYO point for carriers and noncarriers. Shaded areas represent the 99% credible interval. Aβ42 and Aβ40 measurements were from the Fujirebio Lumipulse assay, whereas the SMOC1 measurement was from SRM-MS. L/H, ratio of endogenous peptide signal (light) to the isotopically labeled standard peptide signal (heavy).

**Fig. 2:**
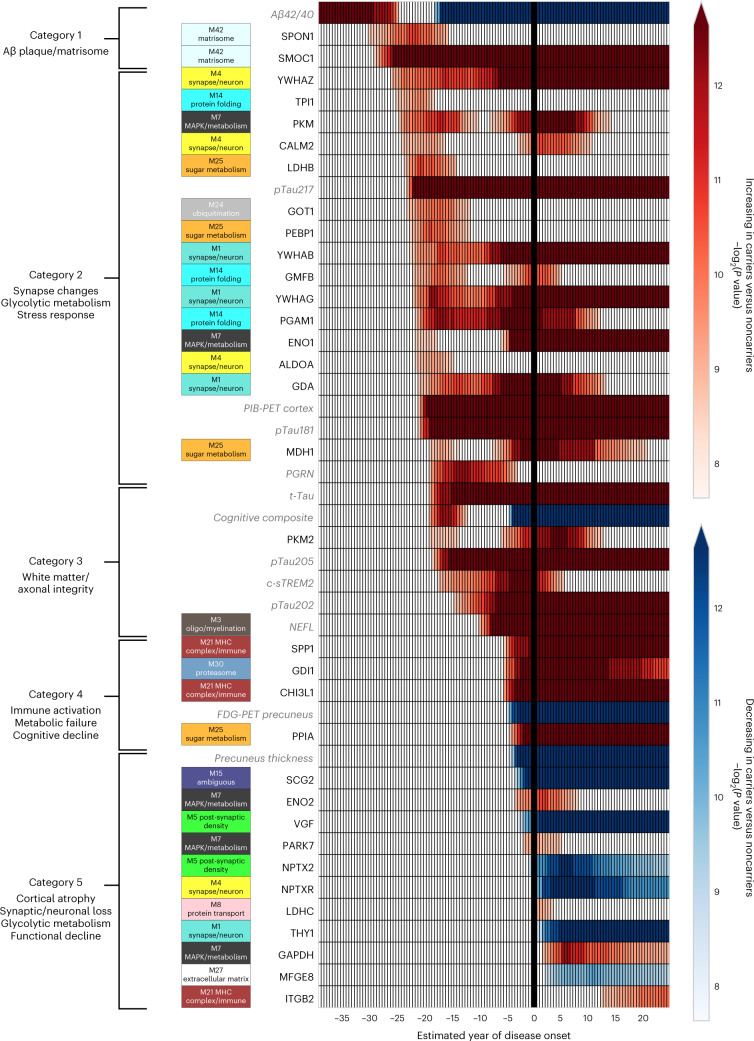
Categories of biomarker changes by EYO in ADAD. Differences between ADAD mutation carriers and noncarriers in levels of CSF biomarker proteins, imaging measures and cognitive function were modeled across the disease course by EYO. Heat represents significant differences between mutation carriers and noncarriers, with the color threshold set at the 99% credible interval (red, increased in carriers; blue, decreased in carriers). All CSF proteins were measured by MS except for PGRN, c-sTREM2 and NEFL, which were measured by ELISA as previously described^20,38,68^. Aβ42/40 ratio was measured by the Fujirebio Lumipulse ELISA assay. Additional biomarker measurements are provided in Extended Data Fig. 1. Biomarker measurements available in DIAN used to benchmark the targeted proteomic measurements are shown in gray italics. CSF proteins were mapped to the corresponding AD brain coexpression module as described in ref. ^8^. Unmapped proteins were not measured in brain. Targeted proteins are listed by their gene symbols. UniProt accessions for each targeted protein are provided in Supplementary Table 2. ALDOA, fructose-bisphosphate aldolase A; CALM2, calmodulin-2; ENO1, alpha-enolase; ENO2, gamma-enolase; FDG-PET precuneus, FDG-PET precuneus signal; GAPDH, glyceraldehyde-3-phosphate dehydrogenase; GDA, guanine deaminase; GDI1, rab GDP dissociation inhibitor alpha; GMFB, glia maturation factor beta; GOT1, aspartate aminotransferase; ITGB2, integrin beta-2; LDHB, l-lactate dehydrogenase B chain; LDHC, l-lactate dehydrogenase C chain; MDH1, malate dehydrogenase, cytoplasmic; MFGE8, lactadherin; NPTXR, neuronal pentraxin receptor; NPTX2, neuronal pentraxin-2; PARK7, parkinson disease protein 7; PEBP1, phosphatidylethanolamine-binding protein 1; PGAM1, phosphoglycerate mutase 1; PKM, pyruvate kinase; PKM2, pyruvate kinase 2; PIB-PET Cortex, PIB-PET total cortex signal; PPIA, peptidyl-prolyl cis–trans isomerase A; SCG2, secretogranin-2; t-Tau, tau peptide 181–190, a marker of total tau levels; THY1, thy1 membrane glycoprotein; TPI1, triosephosphate isomerase; VGF, neurosecretory protein VGF; YWHAB, 14-3-3 protein beta; YWHAG, 14-3-3 protein gamma; YWHAZ, 14-3-3 protein zeta.

A summary of the measurements and cohort is provided in Table 1 and Supplementary Table 1. Our SRM-MS measures provided a relative protein abundance level among all subjects that could be modeled across EYO time points. We employed a Bayesian regression model incorporating a Markov chain Monte Carlo algorithm to estimate, at the 99% confidence level, protein level and other outcome differences between mutation carriers and noncarriers at 0.5 EYO intervals between –30 to –40 and +20 to +30, adjusting for shared genetic background^20^. Sex and apolipoprotein E (*APOE*) ε4 allele status—the strongest genetic risk factor for LOAD—did not significantly influence the results and were therefore not included in the final model. An example of the model fit and difference between carrier and noncarrier for two measures—the Aβ42/40 ratio and SPARC-related modular calcium-binding protein 1 (SMOC1)—is shown in Fig. 1. A decrease in the Aβ42/40 ratio correlates with the development of Aβ plaques^21^. The SMOC1 protein has been shown to colocalize with Aβ plaques, and is one of the most strongly elevated proteins in asymptomatic AD cortex^22^. Each protein was placed within the context of the biological process to which it could be ascribed using a recently published consensus proteomic analysis of AD brain^8^. Of the 59 proteins measured by SRM-MS, 33 were significantly different at the 99% credible interval between ADAD mutation carriers and noncarriers at some EYO time point, with most changing before onset of symptoms (Fig. 2 and Supplementary Information).

**Table 1:** Study participants

	Noncarriers (*N* = 230)			Mutation carriers (*N* = 355)			
	*n* (%)	Mean	s.d.	*n* (%)	Mean	s.d.	*P* value
Age^a^		38.1	11.2		38.4	10.8	0.7
Sex (male)	96 (42)			158 (45)			
Mutation							
*APP*				65 (18)			
*PSEN1*				264 (74)			
*PSEN2*				26 (7)			
*APOE* genotype							
ε2/2	3 (1)			2 (1)			
ε2/3	22 (10)			35 (10)			
ε2/4	7 (3)			8 (2)			
ε3/3	134 (58)			215 (61)			
ε3/4	61 (27)			87 (25)			
ε4/4	3 (1)			8 (2)			
EYO^a^		−9.4	11.2		−7.4	10.9	0.01
SRM-MS protein measurements	184 (80)			286 (81)			
Aβ42/40 ratio	196 (85)	0.09	0.01	319 (90)	0.07	0.03	5.67 × 10^−12^
pTau181 (ng ml^−1^)	152 (66)	0.09	0.03	230 (65)	0.19	0.15	3.50 × 10^−15^
pTau202 (ng ml^−1^)	152 (66)	0.01	0.005	230 (65)	0.02	0.009	1.75 × 10^−8^
pTau205 (ng ml^−1^)	151 (66)	0.002	0.001	230 (65)	0.005	0.006	2.58 × 10^−12^
pTau217 (ng ml^−1^)	152 (66)	0.004	0.004	230 (65)	0.03	0.04	9.90 × 10^−15^
t-Tau (ng ml^−1^)	152 (66)	0.41	0.14	230 (65)	0.60	0.32	1.63 × 10^−11^
NEFL (pg ml^−1^)	192 (83)	250.5	148.1	304 (86)	510.0	529.3	8.92 × 10^−11^
PGRN (pg ml^−1^)	161 (70)	745.1	248.5	250 (70)	816.5	266.8	0.007
c-sTREM2 (pg ml^−1^)	151 (66)	3.3	1.34	242 (68)	4.0	1.6	5.73 × 10^−5^
FDG-PET (SUVR)	163 (71)	1.92	0.16	246 (69)	1.84	0.23	8.33 × 10^−5^
PIB-PET (SUVR)	165 (72)	1.06	0.17	238 (67)	1.95	1.08	2.98 × 10^−23^
MRI (mm^3^)	173 (75)	4.75	0.27	260 (73)	4.58	0.44	5.35 × 10^−6^
Cognitive composite	229 (99)	0.24	0.47	337 (95)	−0.16	0.95	5.54 × 10^−9^

^a^Calculated at the sample level at time of assessment.

Data were from DIAN data freeze 15. Additional trait data are available in Supplementary Table 1. Differences were assessed by two-sided *t*-test without correction for multiple comparisons. pTau202, tau phosphorylated at residue 202; SUVR, standardized uptake value ratio.

**Extended Data Fig. 1:**
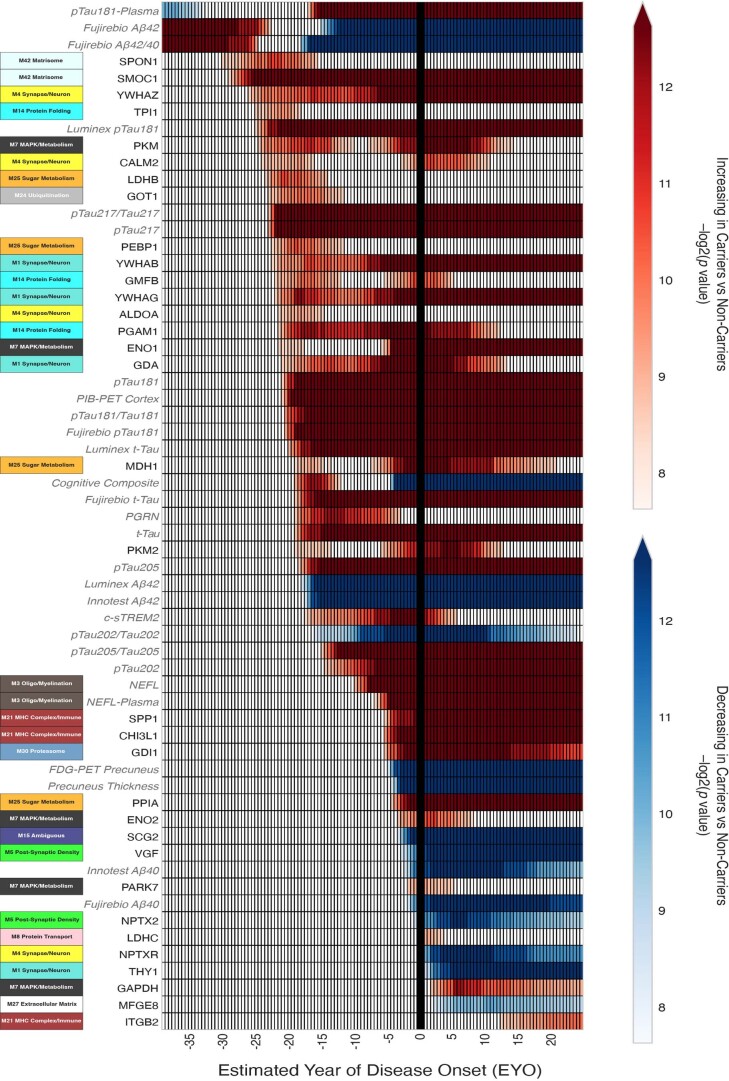
Biomarker Changes by Estimated Year of Disease Onset in ADAD. Data is presented as described in Fig. 2, but includes additional measurements of Aβ and tau species. Biomarker measurements available in DIAN used to benchmark the targeted proteomic measurements are shown in gray italics.

The biomarker changes could be conceptualized into five general categories that evolved over the disease time course. The first category was characterized by proteins associated with an AD brain protein coexpression module we previously termed the ‘M42 matrisome’ module^8^. The ‘matrisome’ refers to the ensemble of proteins associated with the extracellular matrix^23^. M42 matrisome contains the amyloid precursor protein (considered a surrogate measurement for total Aβ levels in MS-based proteomics of AD brain) as well as multiple proteins that have been shown to colocalize with Aβ plaques likely through interactions mediated by heparin-binding domains^22,24–26^. One of these proteins is apolipoprotein E (*APOE*), genetic variation in which has been shown to influence brain M42 matrisome levels^8^. Remarkably, SMOC1—a principal driver of M42 matrisome coexpression in brain—was found to be elevated in mutation carriers 29 years before the onset of symptoms and progressively increased throughout the disease course. The increase in SMOC1 levels preceded a significant decrease in absolute levels of CSF Aβ42 or Aβ42/40 ratio compared with noncarriers that is typically associated with the formation of Aβ plaques^27^, and before elevation in phosphorylated tau at residues 181 and 217 (pTau181 and pTau217)—two markers that have also been shown to increase with initial brain Aβ deposition^28–30^. This finding was observed across different Aβ and tau assays used for measurement of these proteins (Extended Data Fig. 1), and before changes in Aβ plaque deposition were measurable by PET using the radiotracer Pittsburgh Compound-B (PIB-PET). We observed similar early elevation in the level of spondin 1 (SPON1), another member of the M42 matrisome module, although unlike SMOC1 elevation of SPON1 did not persist throughout the disease course.

A second category could be identified after matrisome changes that was characterized by an increase in the 14-3-3 family of proteins YWHAZ (1433Z), YWHAB (1433B) and YWHAG (1433G) associated with synaptic and neuronal coexpression, as well as multiple proteins associated with intermediary glycolytic metabolism including pyruvate kinase, l-lactate dehydrogenase B chain, fructose-bisphosphate aldolase A and phosphoglycerate mutase 1 that mapped to a diverse set of AD brain coexpression modules. Interestingly, although the 14-3-3 proteins were significantly elevated at approximately −26 to −22 EYO, their levels did not begin to rapidly increase until −8 EYO, approximately the time at which neurofilament light chain (NEFL)—a well-known marker of neurodegeneration for multiple central and peripheral nervous system disorders^31^—also began to increase. The early elevations in proteins involved in glycolytic metabolism did not persist throughout the disease course, with a peak at approximately −17 EYO, followed by a period of similar levels compared with noncarriers until around symptom onset, when levels were again elevated. The early period of glycolytic metabolic change was associated with elevation in other protein markers that may reflect an early compensatory neuroprotective response, such as progranulin (PGRN), aspartate aminotransferase, glia maturation factor beta and phosphatidylethanolamine-binding protein 1. PGRN is a secreted factor that has been shown to promote neuronal survival and integrity^32^. Aspartate aminotransferase acts as a scavenger of excess glutamate in the brain and is involved in redox metabolism and the regulation of hydrogen sulfide production important for neuroprotection^33–35^. Glia maturation factor beta is involved in the stimulation of neural regeneration^36^. Phosphatidylethanolamine-binding protein 1 is a negative regulator of the mitogen-activated protein kinase (MAPK) cascade and is also involved in the proper function of presynaptic cholinergic neurons in the central nervous system^37^. Interestingly, early elevation of these proteins coincided with a period of improved cognitive function in mutation carriers compared with noncarriers.

**Fig. 3:**
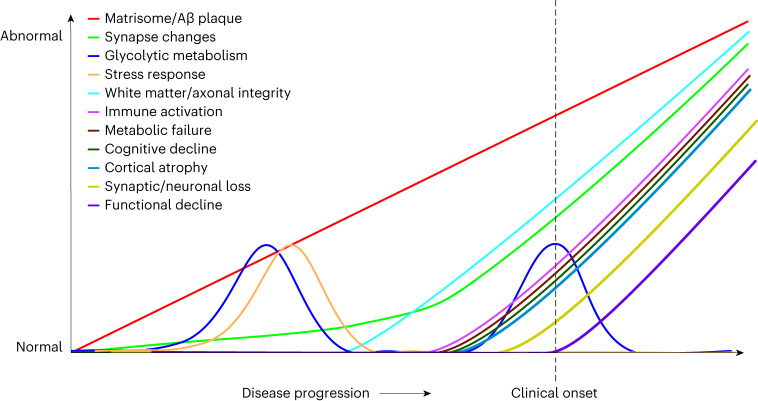
Proposed biomarker cascade in ADAD. The magnitude of change depicted by the *y* axis is arbitrary, and magnitudes are not comparable across different biomarker categories.

A third category of changes could be identified beginning at approximately −19 EYO with elevation in total tau (t-Tau) and tau phosphorylated at residue 205 (pTau205) levels, followed soon after by mild elevation in the cleaved soluble form of triggering receptor expressed on myeloid cells 2 (c-sTREM2) associated with microglial activation^38,39^, and eventual elevation in NEFL beginning at −10 EYO^20^. Elevated levels of pTau205 and NEFL have been associated with loss of white matter and axonal integrity^40,41^. The time span between the elevation in t-Tau and pTau205 levels and elevation in NEFL levels was, therefore, nearly 10 years, suggesting a long period of evolving axonal and white matter changes. Elevation in NEFL was followed by a fourth category of changes beginning at approximately −6 EYO that was characterized by increases in inflammatory proteins osteopontin (SPP1), chitinase-3-like protein 1 (CHI3L1, also known as YKL-40), and more intense elevation in c-sTREM2. SPP1 is a multifunctional protein that has been associated with T lymphocyte and microglial activation^42,43^, whereas CHI3L1 is associated with astrocyte activation^44,45^. These inflammatory changes coincided with gross metabolic impairment as assessed by a decreased fluoro-2-deoxy-d-glucose positron emission tomography (FDG-PET) signal, and the onset of cognitive decline. A fifth and final category of changes included the onset of brain atrophy and decreases in neuronal and neurosecretory proteins such as secretogranin-2, VGF, thy1 membrane glycoprotein, and neuropentraxin and its receptor, suggesting frank synaptic and neuronal loss. A second phase of increased glycolytic metabolism was present during this period with elevation in proteins associated with the M7 MAPK/metabolism and M25 sugar metabolism brain modules including malate dehydrogenase, alpha- and gamma-enolase, pyruvate kinase and pyruvate kinase 2, peptidyl-prolyl *cis*–*trans* isomerase A and glyceraldehyde-3-phosphate dehydrogenase. A general scheme summarizing biomarker progression over the disease course is provided in Fig. 3. Additional rationale for categories is provided in the Supplementary Information.

### The proteome strongly discriminates mutation carriers from noncarriers before symptom onset

**Fig. 4:**
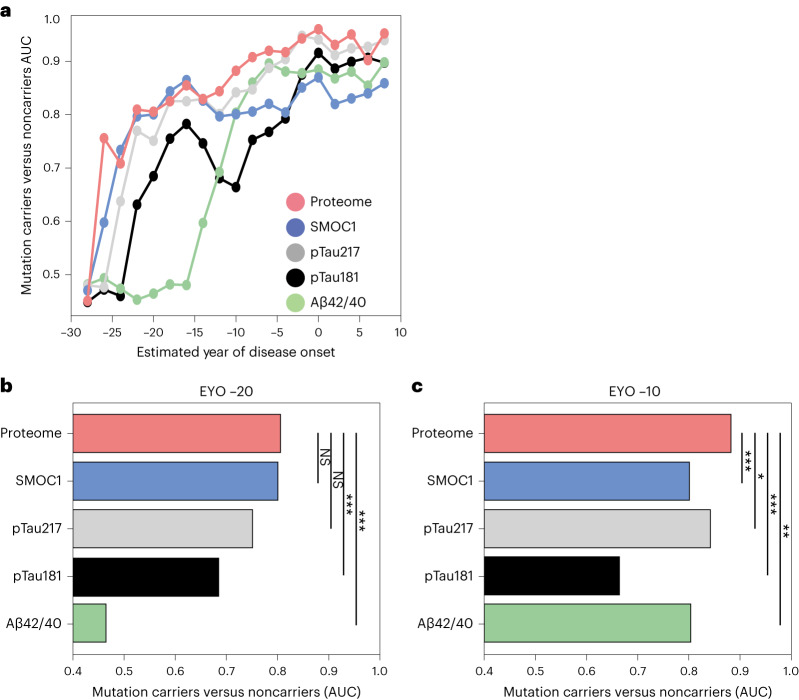
Discrimination of ADAD mutation carriers from noncarriers. **a**, The ability of Aβ42/40, pTau181, pTau217, SMOC1 and a composite of 33 proteins (proteome) to discriminate mutation carriers from noncarriers across the disease course was assessed using the AUC (higher values equal better discrimination). Each point indicates classification performance (AUC) for carriers and noncarriers over a 10-year time window centered at that particular time point. **b**, AUC of the ROC curve for each measure with the 10-year time window centered at EYO −20. **c**, AUC of the ROC curve for each measure with the 10-year time window centered at EYO −10. Significant differences between the proteome and other measures were determined using a nonparametric permutation procedure as described in Methods. The resulting two-sided *P* values were not corrected for multiple comparisons. *P < 0.05, **P < 0.01, ***P < 0.001. NS, not significant.

We assessed the ability of SMOC1 and a composite of the targeted 33 proteins significantly altered in ADAD mutation carriers to correctly categorize carriers from noncarriers across the disease time course compared with current and emerging pTau biomarkers (Fig. 4). Both SMOC1 and the proteome composite measure compared favorably with amyloid and tau biomarkers, particularly in the very early stages of the disease.

## Discussion

In this study we used targeted proteomics to relate biomarker changes in AD CSF to brain pathological changes over the course of six decades. We found that SMOC1 and SPON1—two proteins from the M42 matrisome AD brain coexpression module related to brain Aβ deposition—were elevated in AD CSF nearly 30 years before the onset of symptoms, and before a significant decrease in CSF Aβ42 levels or Aβ42/40 ratio, increase in PIB binding or increase in levels of different pTau species related to Aβ plaque formation. SMOC1, like other M42 proteins, has been shown to colocalize with Aβ plaques^22^. It has also been shown to be elevated in the preclinical stage of sporadic AD and is increased in both AD CSF and plasma by affinity-based proteomic measurement^46,47^. SMOC1 is therefore a promising biofluid AD biomarker of brain Aβ deposition that may be particularly useful in the context of early detection of Aβ plaques and assessment of their clearance with anti-Aβ immunotherapies. Further proteomic analysis of AD biofluids may reveal other promising M42 biomarker proteins.

The M42 matrisome class of proteins, of which Aβ is a member, may not only contain promising AD biomarkers, but also represent promising new therapeutic targets for the disease. M42 proteins may mediate the pathologic effects of Aβ plaques through either gain or loss of function as a consequence of physical interactions with plaques—interactions which themselves may modulate the dynamics of plaque formation. *APOE*, which is the strongest common genetic risk factor for AD and is a member of the M42 matrisome module^8,48^, likely associates with Aβ plaques through its heparin-binding domain similar to other M42 proteins. Notably, the Christchurch *APOE* mutation (*APOEch*) eliminates the ability of the protein to bind heparin, and this mutation has been shown to afford remarkable protection against ADAD^49^. The APOE ε2 allele, protective against LOAD, also has reduced heparin-binding activity^49,50^. Modulation of Aβ plaque interaction with other M42 proteins may afford similar disease benefit. One of these M42 proteins, vascular endothelial growth factor receptor 1, is a receptor tyrosine kinase that activates the MAPK signaling cascade^51^. Early dysfunction in its biology may lead to downstream activation of MAPK as captured by the brain M7 MAPK/metabolism module, elevation of which we have shown previously to be associated with cognitive decline^8^. Other M42 members such as SPON1 are involved in neurite development and may link Aβ to neuritic dystrophy^52^. Genetic variation in SPON1 has been linked to the rate of cognitive decline in AD^53,54^.

Whereas the first category of CSF biomarker changes was related to M42 proteins, the second category encompassed many proteins related to glycolytic metabolism that were associated with multiple different brain modules. In an early consensus AD brain proteomic study, we observed increased markers of glycolytic metabolism that appeared to be associated with astrocyte and microglial activation^9^. However, more recent AD brain proteomic work has suggested that coexpression modules associated with glycolytic metabolism are not necessarily specific to any single brain cell type^9,46^. Changes in glucose metabolism may be shared by multiple brain cell types. For instance, an increase in glycolysis in neurons in the presence of Aβ has been observed^55^, while microglia are also known to increase glycolytic flux as they engage Aβ plaques for phagocytosis^39,56,57^. Astrocytes have also been proposed to increase glucose metabolism in early stages of the disease^58^. The early increase in metabolic markers that followed the increase in M42 markers was associated with increases in other proteins likely associated with a compensatory response, and may represent a response by neurons or other cell types to stress induced by aggregated Aβ. Interestingly, the early elevation in metabolic markers did not persist throughout the disease course, but a second elevation occurred concurrently with the time of intense immune activation, as represented by increases in c-sTREM2, SPP1 and CHI3L1 levels that immediately preceded metabolic impairment as indicated by a reduced FDG-PET signal, rapid neurodegeneration and cognitive decline. It is possible that the astroglial response during this period leads to a reduction in homeostatic metabolic support to neurons via a reduction in the astrocyte–neuron lactate shuttle^59^, with subsequent impairment of neuronal metabolism leading to a reduced FDG-PET signal. It is also possible that this second phase of elevated glycolytic metabolism may represent strong glial activation to dying neurons. Further studies using approaches that can resolve metabolic changes at the single cell level will likely be required to more precisely identify which cell types are driving the observed increased levels of metabolic markers in CSF at a given stage in the AD disease course.

The 33 proteins when considered together were better able to discriminate carriers from noncarriers compared with Aβ or pTau181, especially at early stages of the disease, and had similar classification performance to pTau217. Additional diagnostic information is likely available through proteomic measurements in CSF and plasma that provide greater coverage beyond the analysis presented here. Such multidimensional proteomic data will be important in subtyping and staging AD for precision medicine approaches to the disease.

Our findings provide a relative time frame between observed biomarker changes over the disease course. Absolute time estimates of biomarker changes will likely skew to earlier time points as the size of the DIAN cohort grows and estimates of biomarker differences between mutation carrier and noncarriers increase in confidence. However, given that our estimates were at the 99% credible interval, we do not expect most absolute time estimates to change dramatically and that the relative ordering of marker changes will remain consistent with additional data. Autosomal dominantly inherited forms of AD and sporadic LOAD have been shown to have similar pathophysiology^14,60^, but it is possible that there may be differences between ADAD and LOAD that could influence the sequence and degree of biomarker changes observed. For instance, although multiple neuropathologies are present in a substantial proportion of both ADAD and LOAD cases, ADAD cases tend to have a higher Aβ plaque and NFT burden, higher cerebral amyloid angiopathy burden, and lower Lewy body and microvascular disease burden compared with LOAD^61^. TAR DNA-binding protein 43 aggregation is also more common in aged individuals with LOAD^62^. Another difference is that ADAD is associated with overproduction of Aβ42, whereas LOAD is associated with reduced brain Aβ42 clearance^12,63^. Overproduction of Aβ42 may increase the time between Aβ plaque formation and decreased CSF levels of this marker when compared with mutation noncarriers. It may also affect the point at which Aβ deposition plateaus in ADAD and LOAD^49,64,65^. In our study, we did not observe a significant effect of *APOE* ε4 on biomarker changes, consistent with the lack of effect of *APOE* ε4 on disease onset previously observed in ADAD^66^. This is in contrast to LOAD, where *APOE* ε4 has a significant effect on AD biomarkers and disease onset^67^. Finally, although the DIAN cohort is quite young (average age 38 for carriers and noncarriers), LOAD biomarkers that may change many decades before symptom onset in mutation noncarriers could affect estimated differences between mutation carriers and noncarriers. Further studies on ADAD brain proteomics, and LOAD progression over the course of many decades through studies such as the Alzheimer’s Disease Neuroimaging Initiative, will be required to more fully examine potential differences between ADAD and LOAD.

Our study demonstrates how AD pathology evolves over the course of the disease, and suggests there may be at least three critical periods for therapeutic intervention in ADAD and also likely LOAD: (1) the onset of amyloid plaque formation 30 years before the onset of cognitive symptoms; (2) the onset of axonal and white matter integrity problems starting 19 years before symptoms; and (3) the strong inflammatory response beginning 6 years before symptoms that is proximate to cognitive decline and cortical atrophy. Targeting pathological changes in each category for therapeutic intervention will likely be most successful before, at or near the onset of such changes. Once an individual develops symptoms, a multitarget therapeutic approach will likely be required to optimally slow disease progression.

## Methods

### Participants

Individuals at 50% risk of carrying an autosomal dominant Alzheimer’s disease mutation in one of three genes (*APP, PSEN1, PSEN2*) were enrolled in the DIAN observational study (that is, mutation carriers and noncarriers from the same family). DIAN participants are assessed at baseline and at subsequent follow-up visits that occur every one to three years. Assessments included collection of body fluids (CSF, blood), clinical testing (Clinical Dementia Rating (CDR)), neuropsychological testing and imaging modalities (magnetic resonance imaging (MRI), PIB-PET and 18F-FDG) as previously described^13,69^^–^^72^. The institutional review board at Washington University in St Louis provided supervisory review and human studies approval. Participants or their caregivers provided informed consent in accordance with their local institutional review boards. Details on the number of participants and number of measurements for each trait analyzed in this study are provided in Supplementary Table 1, which was generated using scipy v.1.9.3. Data were from DIAN data freeze 15.

### Clinical assessment and EYO

The presence of symptoms was assessed using the CDR^71^. Clinical evaluators were blinded to each participant’s mutation status. For every visit, a participant’s EYO was calculated based on their age at the visit relative to their mutation-specific expected age at symptom onset. The mutation-specific expected age of symptom onset was computed by averaging the reported age of symptom onset across individuals with the same specific mutation from the DIAN cohort as well as from the published literature, as previously described^66^. If the mutation-specific expected age at symptom onset could not be calculated because only single families with a mutation were available (8% of participants), the individual EYO was calculated from the age at which the parental cognitive decline began (parental age of onset). The parental age of clinical symptom onset was determined by a semi-structured interview with the use of all available historical data. The EYO was calculated identically for both mutation carriers and noncarriers. As an example, if the expected age of onset for a particular ADAD mutation is 50 and two fraternal twins were aged 40, one of whom is a carrier for the mutation and one of whom is not, they would both have an EYO of −10. The unaffected mutation noncarrier family member therefore serves as a direct control to the mutation carrier, which can help control for subject-specific factors that may be shared between family members. Given the young age of the DIAN cohort (mean age 38), biomarker changes due to the potential development of sporadic LOAD in mutation noncarriers are unlikely to substantially influence the analysis and results reported in DIAN. Mutation status was determined using polymerase chain reaction-based amplification of the appropriate exon followed by Sanger sequencing^13^.

### CSF and plasma sample collection

CSF and blood plasma were collected in the morning under fasting conditions. Blood was drawn into two 10-ml syringes precoated with 0.5 M EDTA, then transferred to two 15-ml polypropylene tubes containing 120 μl of 0.5 M EDTA. The samples were kept on wet ice until centrifugation. After venipuncture, CSF was collected by gravity drip into two 13-ml polypropylene tubes using standard lumbar puncture procedures (L4–L5) with an atraumatic Sprotte spinal needle (22G). Plasma and CSF were flash-frozen upright on dry ice. Samples collected in the United States were shipped overnight on dry ice to the DIAN biomarker core laboratory at Washington University, whereas samples collected at sites outside the United States were stored at −80 °C and shipped quarterly on dry ice to Washington University. At the core laboratory, the frozen samples were subsequently thawed, combined into a single polypropylene tube of plasma or CSF, and aliquoted (300 or 500 μl) into polypropylene Corning microcentrifuge tubes (Thermo Fisher Scientific), after which they were again flash-frozen on dry ice and stored at −80 °C. DIAN CSF samples were shipped to Emory University for SRM-MS analysis.

### Measurement of CSF protein levels by SRM-MS

Fifty-nine proteins previously identified as altered in AD CSF were targeted for measurement by SRM-MS using the ratio of the endogenous proteotypic peptide level to an isotopically labeled heavy standard, according to best practices^9,16,73^. CSF proteins from 475 DIAN baseline samples and 65 quality controls (QC) were analyzed. The QCs were generated from a cohort of Emory subjects by pooling approximately 50 individuals from one of three groups: a biomarker-positive group representing low Aβ and high t-Tau; a biomarker-negative group representing high Aβ and low t-Tau; and a biomarker-intermediate group representing intermediate Aβ and t-Tau levels. The QCs were processed independently in parallel and analyzed identically to the DIAN CSF samples to ensure proper assay performance.

A 95-µl aliquot of CSF was reduced and alkylated with 2 µl of 0.5 M tris-2(-carboxyethyl)-phosphine (Thermo Fisher Scientific, catalog no. 77720), 5 µl of 0.8 M chloroacetamide (Sigma, catalog no. 22790) and 2.5 µl of 1 M ammonium bicarbonate (Sigma, catalog no. 09830) while heating at 90 °C for 10 min, followed by water bath sonication for 15 min. Urea buffer (8 M) made with urea (Sigma, catalog no. U0631), 10 mM Tris (J.T. Baker, catalog no. 4109-06) and 100 mM NaH_2_PO_4_ (Sigma, catalog no. S0751) at pH 8.5 was used as the denaturant. Urea buffer (105 µl) and Lys-C enzyme (5 µg, 1:20 enzyme to protein ratio; Wako, catalog no. 125-02543) were added for overnight digestion at room temperature. The urea was diluted to 1 M with 50 mM ammonium bicarbonate (615 µl) and trypsin (10 µg, 1:10 enzyme to protein ratio; Thermo Fisher Scientific, catalog no. 90058) was added for overnight digestion. Trypsin digestion was stopped by adding final concentration of 1% formic acid (FA; Thermo Fisher Scientific, catalog no. A117) and 0.1% trifluoroacetic acid (TFA; Thermo Fisher Scientific, catalog no. 85183).

Peptides were desalted with 30 mg C18 HLB 96-well plates (Waters, catalog no. 186008054) using a positive pressure system. Each HLB well was conditioned (1 ml of methanol) and equilibrated twice (1 ml of 0.1% TFA) before the samples were added. Each well was washed twice (1 ml of 0.1% TFA) and eluted twice (500 µl of 50% acetonitrile with 0.1% FA). A portion (450 µl) of the solid-phase extraction elution was transferred to new plates for targeted MS analysis. All samples and QCs were dried using a SpeedVac.

Samples were reconstituted in 40 µl of heavy standards (4 µl) and Promega 6 × 5 LC-MS/MS Peptide Reference Mix (50 fmol µl^−1^; Promega, catalog no. V7491) in mobile phase A (0.1% FA in water; Thermo Fisher Scientific, catalog no. LS118). Peptide eluents (20 µl) were separated on an AdvanceBio Peptide Map Guard column (2.1 × 5 mm, 2.7 μm; Agilent, catalog no. 851725-911) connected to an AdvanceBio Peptide analytical column (2.1 × 150 mm, 2.7 μm; Agilent, catalog no. 653750-902) by a 1290 Infinity II system (Agilent) and monitored on an TSQ Altis Triple Quadrupole mass spectrometer (Thermo Fisher Scientific). Sample elution was performed over a 14-min gradient using mobile phase A (0.1% FA in water) and mobile phase B (0.1% FA in acetonitrile; Thermo Fisher Scientific, catalog no. LS120) at a flow rate of 0.4 ml min^−1^. The gradient was from 2% to 24% mobile phase B over 12.1 min, then from 24% to 80% over 0.2 min, and held at 80% mobile phase B for 0.7 min. The mass spectrometer was set to acquire data in positive-ion mode using selected reaction monitoring acquisition. Three transitions were acquired for each target analyte, the cycle time set to 0.8 s, Q1 resolution to 0.7 full-width at half-maximum, Q2 resolution at 1.2 full-width at half-maximum, and collision-induced dissociation gas at 1.5 mTorr. Data were uploaded into Skyline-Daily v.22.2.1.351 for analysis. Total area ratios for each peptide were calculated by summing the area for each light (3) and heavy (3) transition and dividing the light total area by the heavy total area. Each batch included QCs at the beginning, end and after every 20 samples per plate. Using the coefficient of variation for the 30 monitored Promega peptides, we estimated the lowest limits of detection to be between 1 and 10 femtomoles for each peptide. All peptide measurements had coefficients of variation less than 30%, with most less than 20% (Supplementary Table 2). We used the light peptide signal within a sample to determine sample quality. Based on our inspections, two DIAN identifiers were removed from our matrix because the sample quality was deemed unacceptable. A total of 470 subjects with sufficient trait data were included in the final statistical analysis of the SRM protein measurements. Gene symbols for each targeted protein in this study were used to maintain consistency with brain proteomic data and to facilitate integration with other -omics data. UniProt accessions and peptide sequences for all targeted proteins are provided in Supplementary Table 2.

### NonSRM-MS molecular biomarker measurements

MS-based measurements of tau and pTau species used in this analysis have been previously described^60^. ELISA measurements of Aβ, tau and pTau were obtained using the Luminex, Fujirebio and Innotest platforms^13^. Plasma pTau181 and NEFL ELISA measurements were obtained on the Simoa HD-1 platform as previously described^20^. PGRN and c-sTREM2 measurements were obtained on the Meso Scale Discovery platform as previously described^38,68^.

### Imaging

Imaging protocols and data processing for MRI and PET studies in DIAN have previously been described in detail^69,70^. We used the precuneus region for cortical thickness and metabolic imaging analyses given that it has been shown to be the region most sensitive to early AD changes in ADAD^69^. Precuneus measurements were averaged across hemispheres. For PIB-PET, we used the total cortical mean signal. PET measurements were corrected for partial volume effects.

### Cognitive measures

In this analysis we used the Mini Mental State Examination (MMSE) and a composite cognitive measure^72^. The cognitive composite measure was generated by converting four different cognitive outcomes measures into *z*-scores, then averaging the four *z*-scores into one composite measure. The outcome measures used for the composite were animal naming (DIAN variable ANIMALS), digit symbol substitution (DIAN variable WAIS), delayed logical memory (DIAN variable MEMUNITS) and the MMSE.

### Statistical analysis

#### Bayesian modeling

We analyzed each participant’s first CSF and plasma measurement in this study. Measures for all protein biomarkers underwent log_2_ transformation to approximate normality before analysis. Measurements greater than five standard deviations from the mean after log_2_ transformation were removed before analysis. Inclusion of outliers did not significantly alter the analysis.

We carefully studied the variables that could be used to model the cross-sectional CSF and plasma outcomes. We did not include age in our model because it is highly correlated with EYO. Our ad hoc analysis also revealed that adding commonly utilized predictors, such as sex and APOE ε4 status, did not provide any additional benefit to our model for modeling phenotypic outcomes in AD. The independent variables in our final model included ADAD carrier/noncarrier status and EYO.

To better approximate the complex nonlinear relationships between the biomarkers and EYO, and according to previously published work^20^, we modeled EYO using a restricted cubic spline transformation with three knots at the 0.1, 0.5 and 0.9 quantiles (Formula 1). The restricted cubic spline transformation decomposes EYO into one linear term and one cubic term, which ensures the resulting fitted curve is smooth and continuous at each quantile segment.

We used a Bayesian framework to analyze the relationship between biomarkers and the independent variables and achieve accurate and robust statistical inference from these family-based samples. The Bayesian framework can account for random effects induced by strong family relatedness. The Bayesian regression model was implemented by Markov Chain Monte Carlo (MCMC)—a powerful and robust MCMC algorithm called the Hamiltonian Monte Carlo algorithm. We implemented the algorithm in R v.4.1.2.

Our primary objective of using the Bayesian method was to provide an estimation of the uncertainty that is associated with the unknown parameters in the generalized linear model (GLM). Through quantifying this uncertainty, we aimed to derive insights into the changes in biomarker levels across EYO. Because our model was designed to be objective, we expect that the posterior distribution of the biomarker levels is not significantly impacted by the prior information. We used the default R package settings to implement flat or weak informative priors. Combined with the moderate sample size, this approach enabled us to obtain posterior estimates that closely approximated the likelihood, aligning with our goals of utilizing the Bayesian framework. Furthermore, by plotting the fitted model, we were able to visualize that the expected biomarker levels at specific EYO produced by our Bayesian GLM aligned well with the observed data points, serving as a sanity check and confirming that the posterior distribution was not significantly influenced by the prior information. Therefore, we do not expect the results to change with different sets of noninformative priors or flat priors.

We applied the Bayesian GLMs with identify link function for continuous outcomes. Our independent variables of fixed effects included ADAD status, linear EYO term, cubic EYO term and the interaction effects between ADAD status and EYO (Formula 2). We selected weak informative Cauchy distribution (location parameter was 0 and scale parameter was 2.5) as the prior distribution of the regression coefficients and the intercept because our method aimed to utilize a more objective data-driven approach. For the MCMC simulation setup, we initialized eight Markov chains using four cores, and each Markov chain generated 10,000 iterations, including a warmup period of 5,000 iterations that were discarded. We also kept every ten simulations for the post-warmup sampling realizations. To ensure that the 4,000 post-warmup samples were a reliable representation of the posterior estimates for both the main effects and the interaction effects, we meticulously examined and tracked the convergence of the parameter estimates. Finally, we estimated the two-sided Bayesian credible interval of the continuous outcomes for ADAD mutation carriers and noncarriers and the credible interval of the difference between carriers and noncarriers. The empirical *P* value was also estimated to measure the probability that carrier and noncarrier were different under the null hypothesis. All estimates were performed at each EYO in 0.5-unit increments. Results were visualized using ggplot2 (v.3.3.6) (Fig. 1) and in a heatmap (Fig. 2) generated using custom Python v.3.10.8 code with the packages seaborn v.0.12.1 and matplotlib v.3.6.2. The Bayesian GLMs were implemented using the open-source R package rstanarm (v.2.21.3).

Our study had two categorical outcomes, CDR global score and the MMSE score, which have ceiling and floor effects that could not be adequately handled using a Gaussian distribution (Formula 3). Therefore, we used Bayesian mixed-effect ordinal regression models with a cumulative link function to model the two categorical outcomes (Formula 3)^74,75^. We encoded CDR (CDR = 0, CDR = 0.5 and CDR ≥ 1) and MMSE (MMSE > 24, 19 ≤ MMSE ≤ 24, MMSE < 19) into three categories that generally correspond to cognitively normal, mild cognitive impairment and dementia stages of AD. This led to a natural ordering for the encoded MMSE and CDR. We specified the ordinal regression with cumulative probabilities. We used a flat prior for the regression coefficients, and we used Student’s *t* distribution (degrees of freedom was 3, location was 0 and scale was 2.5) as the prior for the intercepts. With the same MCMC simulation setup (8 chains on 4 cores, each chain had 10,000 iterations with 5,000 warmups, kept every 10 simulations), we estimated the probability of being in one category and its credible interval at each EYO in 0.5-unit increments based on the 4,000 post-warmup posterior coefficient estimates. The Bayesian mixed-effects ordinal regression model was implemented using the open-source R package brms (v.2.18.0).

Formula 1:

splinefit = rcspline.eval(EYO, nk=3, norm = 2, pc = FALSE, inclx=TRUE)

Formula 2:

Formula = Outcome ~ EYO_Spline_Linear + EYO_Spline_Cubic + MUTATION + EYO_Spline_Linear * MUTATION + EYO_Spline_Cubic * MUTATION + (1 | MASTER_FAMID)

stan_BL <- stan_glmer(Formula, data, family=gaussian(), prior = cauchy(), prior_intercept = cauchy(), chains = 8, cores = 4, iter = 10,000, thin = 10)

Formula 3:

Formula = Encoded CDRGLOB ~ EYO_Spline_Linear + EYO_Spline_Cubic + MUTATION + EYO_Spline_Linear * MUTATION + EYO_Spline_Cubic * MUTATION + (1 | MASTER_FAMID)

stan_CDR <- brm(f, data = BL_traits_pep, family = cumulative, chains = 8, cores = 4, iter = 10,000,

thin = 10)

MMSE is modeled by replacing CDR with MMSE.

### Classification

For the classification analysis, 313 subjects (188 mutation carriers, 125 noncarriers) were analyzed who had measurements of Aβ42/40 ratio, pTau217, pTau181, SMOC1 and the panel of 33 proteins measured by SRM (proteome) at a given EYO. The participants were separated into 10-year time windows spaced 2 years apart based on their EYO. All time windows without a minimum of 30 participants were excluded. For each 10-year time window, logistic regression classifiers with elastic net regularization were trained with fivefold cross-validation to estimate mutation status using Aβ42/40 ratio, pTau217, pTau181, SMOC1 and the proteome measure using Custom Python v.3.9 code and sklearn v.0.24.2. The best L1 ratio for regularization was selected using a fivefold cross-validation procedure within the training set. Performance was assessed using the area under the receiver operating characteristic (ROC) curve (AUC) of the testing sets.

A nonparametric permutation procedure was used to compare performance of the logistic regression models trained using the proteome and other biomarkers. Our null hypothesis was that across participants the proteome showed no difference in AUC compared with the other biomarkers. We computed the true difference in performance between the proteome and the other biomarkers. We then randomly permuted the estimation generated by the proteome and the other biomarkers for each participant and recomputed the difference in performance^76^. Significance was established using 1,000 permutations.

### Reporting summary

Further information on research design is available in the Nature Portfolio Reporting Summary linked to this article.

## Online content

Any methods, additional references, Nature Portfolio reporting summaries, source data, extended data, supplementary information, acknowledgements, peer review information; details of author contributions and competing interests; and statements of data and code availability are available at 10.1038/s41591-023-02476-4.

### Supplementary information

Supplementary Information Supplementary Discussion of categories and additional biomarker plots.

Reporting Summary

Supplementary Tables Supplementary Tables 1 and 2 containing cohort information and SRM-MS QC statistics.

## Data Availability

DIAN trait data are available through request. Instructions can be found at https://dian.wustl.edu/our-research/for-investigators/dian-observational-study-investigator-resources/data-request-terms-and-instructions/. Source data are under controlled access to protect mutation carrier confidentiality. Data requests will be reviewed based on scientific merit and feasibility, appropriateness of the investigator’s qualifications and resources to protect the data, and appropriateness to DIAN goals/themes. De-identified DIAN data will be made available to investigators to conduct analyses after approval by the PI and the relevant DIAN Core Leader. The data request form can be found at https://dian.wustl.edu/our-research/for-investigators/dian-observational-study-investigator-resources/data-request-form/. Data access requests are typically processed within 30–60 days.

## Code availability

Code used for analyses in this study is available upon request.
