# Supplementary material for: Cerebrospinal fluid proteomics define the natural history of autosomal dominant Alzheimer’s disease
Source: Nat Med. 2023 Aug 7;29(8):1979–88. doi: 10.1038/s41591-023-02476-4 (PMC10427428; doi:10.1038/s41591-023-02476-4)
Supplement: Supplementary file 1 — Supplementary Discussion of categories and additional biomarker plots. [file 41591_2023_2476_MOESM1_ESM.pdf]

nature medicine

Article

https://doi.org/10.1038/s41591-023-02476-4

Cerebrospinal fluid proteomics define the natural history of autosomal dominant Alzheimer's disease

In the format provided by the authors and unedited

Category 1 – *Aβ plaque/matrisome*: this category is defined by the Aβ42/40 ratio—a recognized marker of brain amyloid deposition[1]—and two proteins that map to the M42 matrisome brain module, SPON1 and SMOC1.

Category 2 – *Synapse Changes, Glycolytic Metabolism, Stress Response*: these categories are again derived from brain co-expression, where a majority of the proteins fall into either the M1 synapse/neuron, M4 synapse/neuron, M7 MAPK/metabolism, M25 sugar metabolism, or M14 protein folding and M24 ubiquitination modules. We interpret the M14 protein folding module to reflect a stress response. We also interpret the early elevation in PGRN levels to reflect a stress response, although PRGN is not measured in our brain proteomic studies and therefore does not map to a brain module. GOT1 maps to the ubiquitination module. Because GOT1 acts as a scavenger of excess glutamate in the brain and is involved in redox metabolism and the regulation of hydrogen sulfide production important for neuroprotection[2-4], we interpret this protein to be involved in stress response as well. As we note at the beginning of the results section, other protein and non-protein biomarkers measured in DIAN are incorporated into the analysis to serve as benchmarks for the proteomic measures. pTau217, pTau181, and PIB-PET happen to fall into Category 2 based on the time of change, although we are not able to use brain co-expression to map pTau changes directly to the ontologies that describe Category 2 because pTau was not measured in our brain proteomics studies. PIB-PET reflects Aβ plaques, but is a less sensitive measure than the Aβ42/40 ratio for plaques[5,6], and therefore ends up falling into Category 2 based on time of change.

Category 3 – *White Matter, Axonal Integrity*: this category is bounded by t-Tau and NEFL. NEFL consistently maps in our studies to the oligo/myelination module in brain, as it likely reflects loss of myelinated axonal integrity along with pTau205[7,8]. The other markers do not have a cognate brain module because they were not measured in our brain proteomic studies. CSF t-Tau, along with the pTau measurements, was measured as described by Barthelemy *et al*.[9], and was not mapped to MAPT in brain because the brain MAPT mass spec protein measurement is strongly confounded by truncated tau isoforms and does not show a strong increase in AD. This stands in contrast to the MTBR region itself, which shows a strong increase in AD brain by mass spec, but the MTBR is not represented as a canonical protein in the protein search database. Tau binds to microtubules and is considered to be an axonal marker[10], which is why it is included in this category. The cognitive composite measure is another benchmark biomarker in DIAN that falls into this category based on its relative time of change. MDH1 and PKM2 are both involved in glycolysis, although PKM2 does not map to a brain co-expression module. One could plausibly extend Category 2 to include PKM2, which would consequently encompass t-Tau as well into Category 2. However, we decided instead to use t-Tau as the cutpoint for Category 3 based on its relationship to axonal biology.

Category 4 – *Immune Activation, Metabolic Failure, Cognitive Decline*: this category is where we first see proteins associated with the M21 MHC/Immune brain module, SPP1 and CHI3L1. SPP1, or osteopontin, is a multifunctional protein that has been associated with T-lymphocyte and microglial activation[11,12], whereas CHI3L1 is associated with astrocyte activation in brain and is considered to reflect an inflammatory response[13,14]. The metabolic failure term comes from the decrease in the FDG-PET signal. PPIA maps to the M25 sugar metabolism module and may also reflect this metabolic change. The cognitive decline term comes from the decrease in the cognitive composite measure at the same time as the inflammatory changes and change in FDG-PET signal.

Category 5 – *Cortical Atrophy, Synaptic/Neuronal Loss, Glycolytic Metabolism, Functional Decline*: the cortical atrophy term comes from the decrease in precuneus thickness in this

category of changes. The synaptic/neuronal loss term comes from declines in SCG2 (https://www.uniprot.org/uniprotkb/P13521/entry) and VGF (https://www.uniprot.org/uniprotkb/O15240/entry), two well-known neurosecretory proteins, the neuropentraxin proteins NPTX2 and NPTXR that map to synapse/neuron and post-synaptic density modules in brain, and THY1. THY1 maps to the M1 Synapse/Neuron brain module. This conceptually makes sense, as THY1 is strongly expressed in brain (https://www.proteinatlas.org/ENSG00000154096-THY1/tissue) and is also used as a neuron-specific promoter in many transgenic mouse models. The glycolytic metabolism term comes from ENO2, PARK7, and GAPDH that map to the MAPK/metabolism module in brain. L-lactate dehydrogenase C chain (LDHC) could also be considered in this term, although it maps to the M8 protein transport brain module. The term "functional decline" comes from the observation that changes in this category coincide with onset and progression of clinical symptoms as reflected by increase in the Clinical Dementia Rating (CDR). This is shown in the Extended Data plot for CDR. We were not able to include the MMSE and CDR traits in the heatmap because they are ordinal, rather than continuous, traits, and could not be modeled in the same fashion as the other continuous measures.

References

1. Janelidze, S., *et al.* CSF Abeta42/Abeta40 and Abeta42/Abeta38 ratios: better diagnostic markers of Alzheimer disease. *Ann Clin Transl Neurol* **3**, 154-165 (2016).
2. Chen, W.L., *et al.* Neuroprotective effects of hydrogen sulfide and the underlying signaling pathways. *Rev Neurosci* **26**, 129-142 (2015).
3. Dopico-Lopez, A., *et al.* Inhibition of endogenous blood glutamate oxaloacetate transaminase enhances the ischemic damage. *Transl Res* **230**, 68-81 (2021).
4. Kremer, D.M., *et al.* GOT1 inhibition promotes pancreatic cancer cell death by ferroptosis. *Nat Commun* **12**, 4860 (2021).
5. Palmqvist, S., Mattsson, N., Hansson, O. & Alzheimer's Disease Neuroimaging, I. Cerebrospinal fluid analysis detects cerebral amyloid-beta accumulation earlier than positron emission tomography. *Brain* **139**, 1226-1236 (2016).
6. Palmqvist, S., *et al.* Detailed comparison of amyloid PET and CSF biomarkers for identifying early Alzheimer disease. *Neurology* **85**, 1240-1249 (2015).
7. Schultz, S.A., *et al.* Serum neurofilament light chain levels are associated with white matter integrity in autosomal dominant Alzheimer's disease. *Neurobiol Dis* **142**, 104960 (2020).
8. Strain, J.F., *et al.* CSF Tau phosphorylation at Thr205 is associated with loss of white matter integrity in autosomal dominant Alzheimer disease. *Neurobiol Dis* **168**, 105714 (2022).
9. Barthelemy, N.R., *et al.* A soluble phosphorylated tau signature links tau, amyloid and the evolution of stages of dominantly inherited Alzheimer's disease. *Nat Med* **26**, 398-407 (2020).
10. Higuchi, M., Lee, V.M. & Trojanowski, J.Q. Tau and axonopathy in neurodegenerative disorders. *Neuromolecular Med* **2**, 131-150 (2002).
11. Cantor, H. & Shinohara, M.L. Regulation of T-helper-cell lineage development by osteopontin: the inside story. *Nature reviews. Immunology* **9**, 137-141 (2009).
12. Rosmus, D.D., Lange, C., Ludwig, F., Ajami, B. & Wieghofer, P. The Role of Osteopontin in Microglia Biology: Current Concepts and Future Perspectives. *Biomedicines* **10**(2022).
13. Connolly, K., *et al.* Potential role of chitinase-3-like protein 1 (CHI3L1/YKL-40) in neurodegeneration and Alzheimer's disease. *Alzheimers Dement* (2022).
14. Craig-Schapiro, R., *et al.* YKL-40: a novel prognostic fluid biomarker for preclinical Alzheimer's disease. *Biol Psychiatry* **68**, 903-912 (2010).

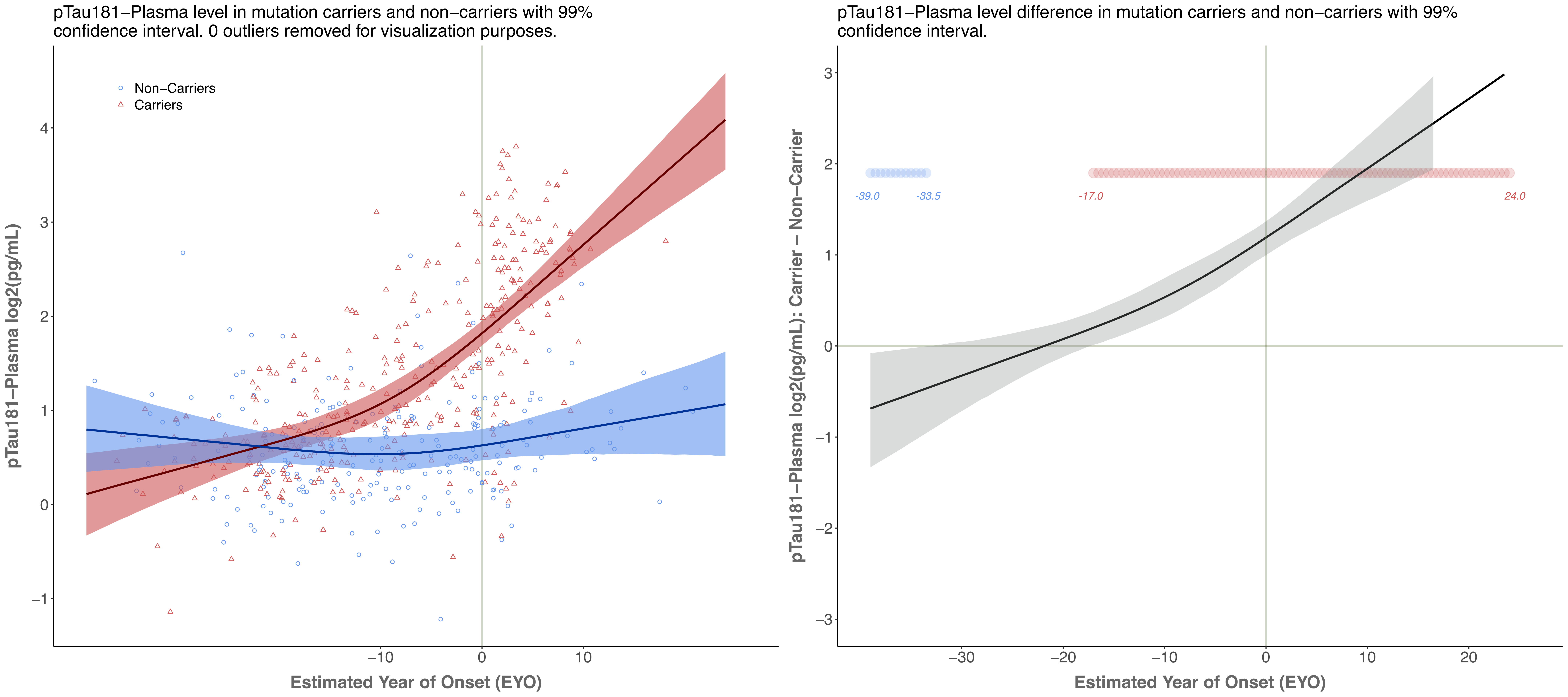

pTau181−Plasma level in mutation carriers and non−carriers with 99% confidence interval. 0 outliers removed for visualization purposes.
Non−Carriers
Carriers
pTau181−Plasma log2(pg/mL)
Estimated Year of Onset (EYO)
−10
0
10
4
3
2
1
0
−1
pTau181−Plasma level difference in mutation carriers and non−carriers with 99% confidence interval.
pTau181−Plasma log2(pg/mL): Carrier − Non−Carrier
-39.0
-33.5
-17.0
24.0
−30
−20
−10
0
10
20
3
2
1
0
−1
−2
−3

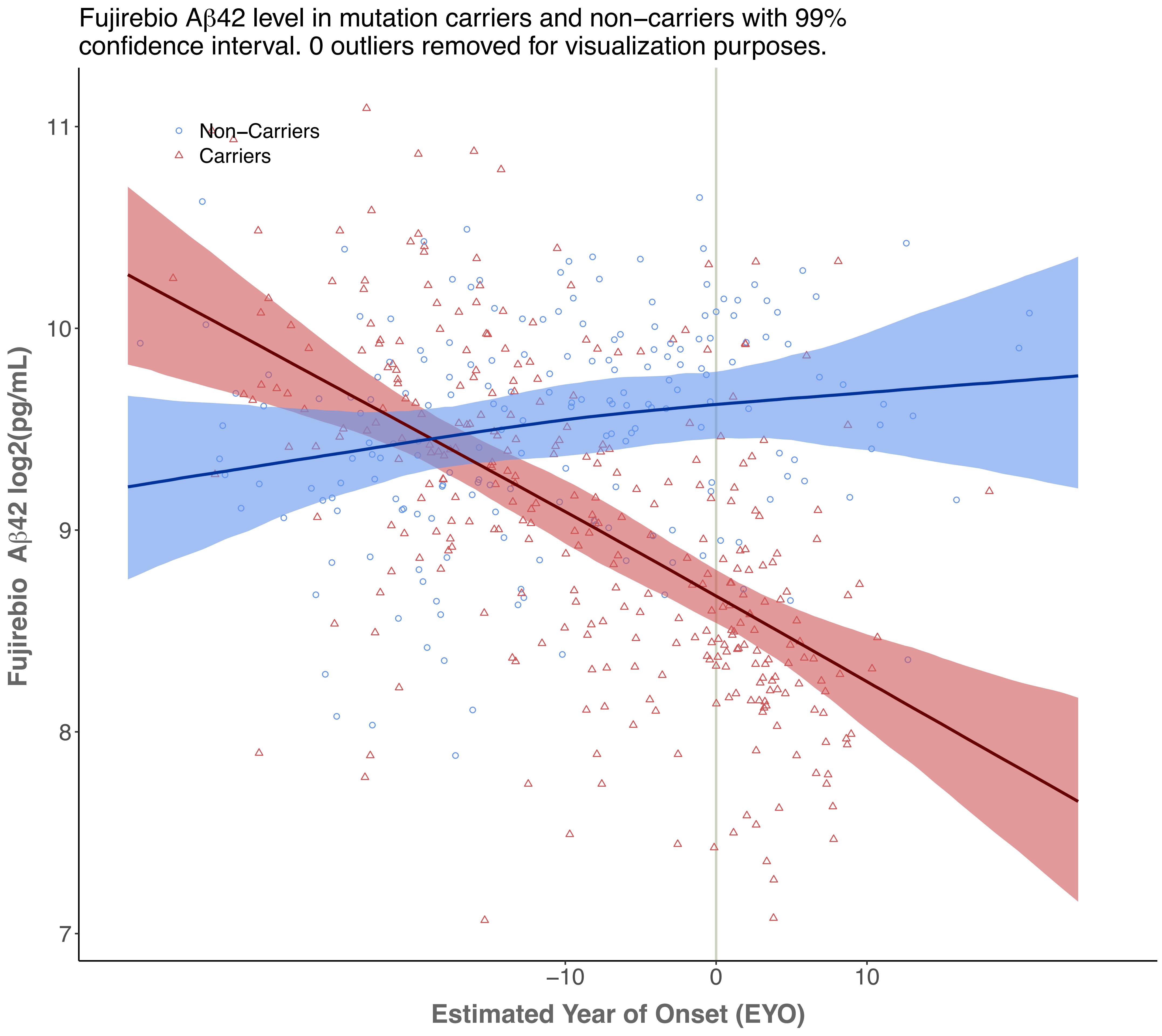

Fujirebio Aβ42 level in mutation carriers and non−carriers with 99% confidence interval. 0 outliers removed for visualization purposes.
Non−Carriers
Carriers
11
10
9
8
7
−10
0
10
Fujirebio Aβ42 log2(pg/mL)
Estimated Year of Onset (EYO)

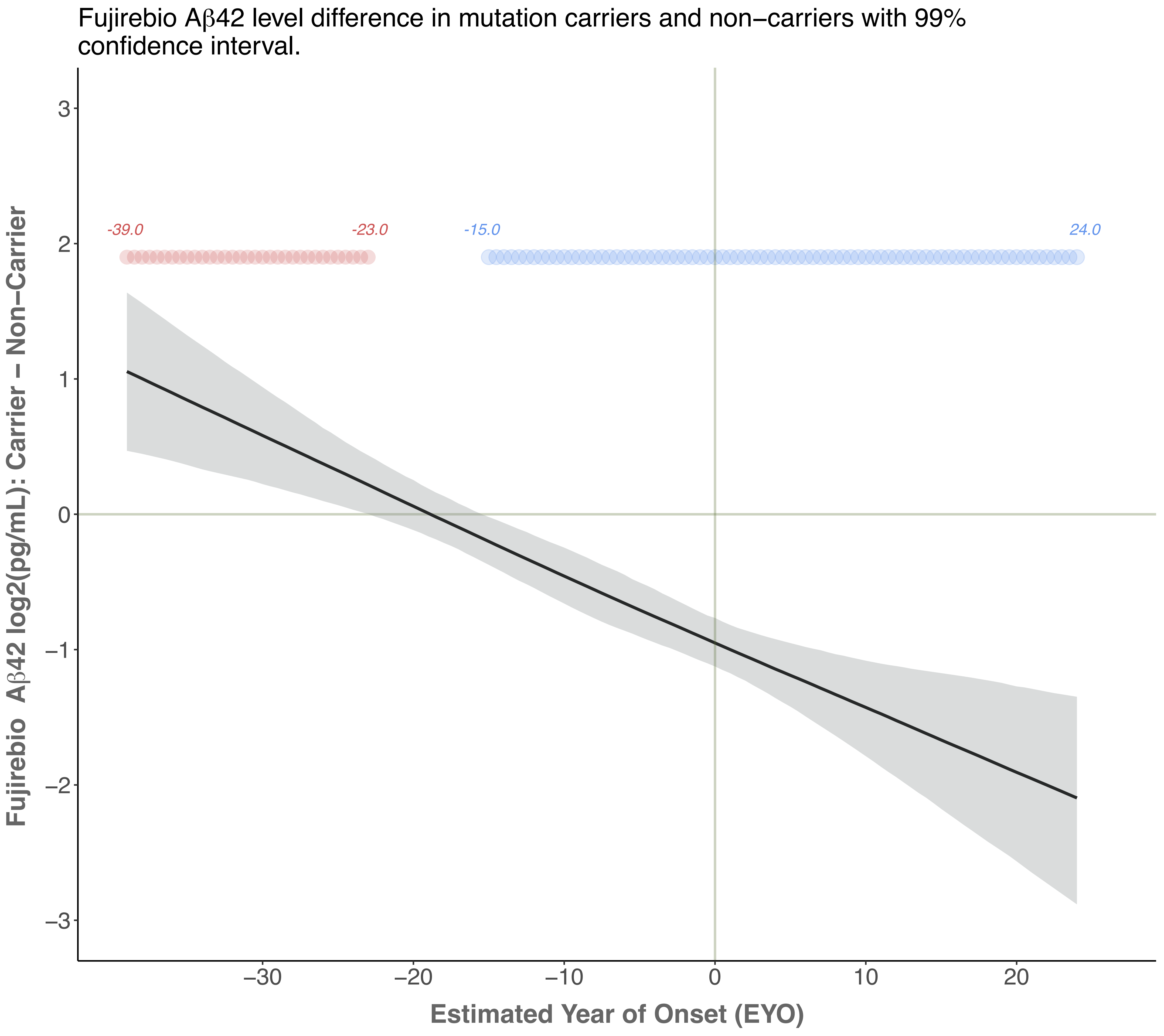

Fujirebio Aβ42 level difference in mutation carriers and non−carriers with 99% confidence interval.
-39.0
-23.0
-15.0
24.0
3
2
1
0
−1
−2
−3
−30
−20
−10
0
10
20
Fujirebio Aβ42 log2(pg/mL): Carrier − Non−Carrier
Estimated Year of Onset (EYO)

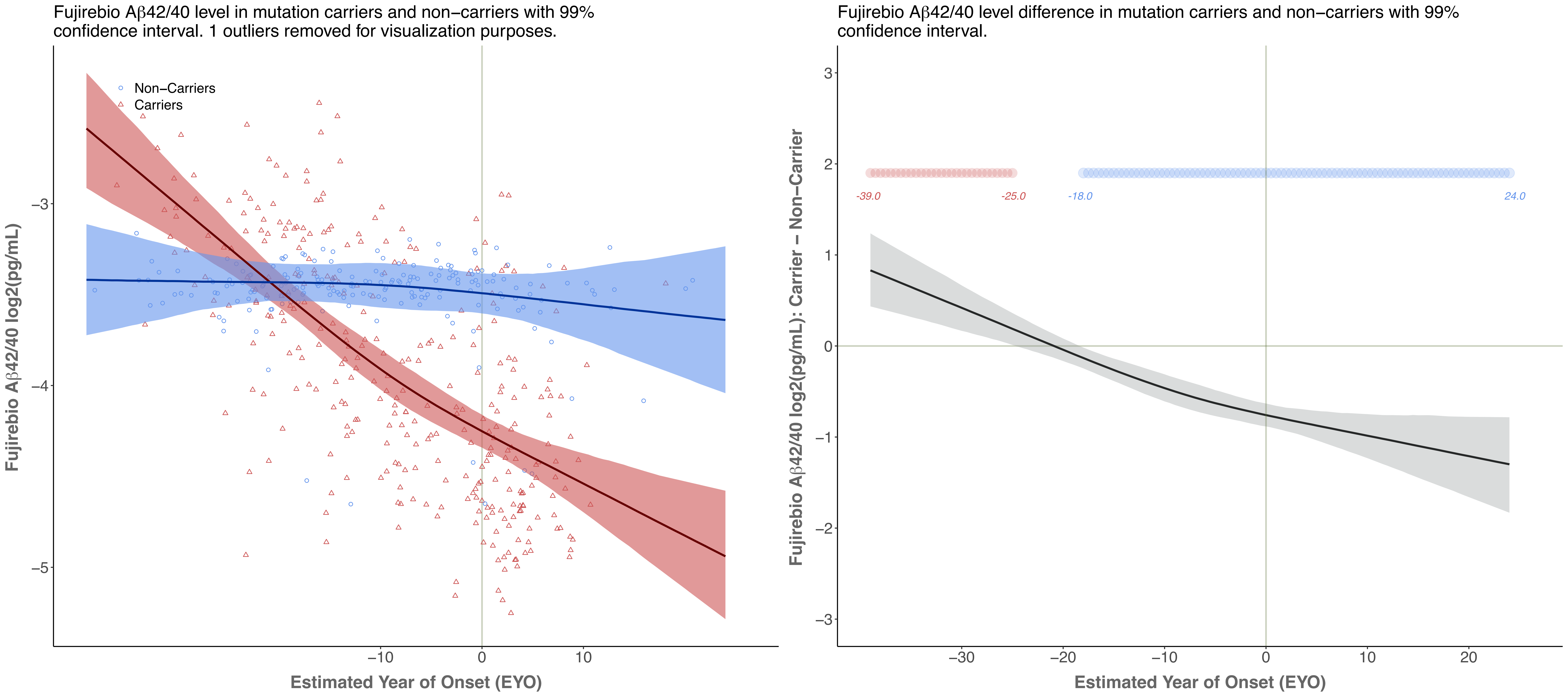

Fujirebio Aβ42/40 level in mutation carriers and non−carriers with 99% confidence interval. 1 outliers removed for visualization purposes.
Non−Carriers
Carriers
Fujirebio Aβ42/40 log2(pg/mL)
−3
−4
−5
−10
0
10
Estimated Year of Onset (EYO)
Fujirebio Aβ42/40 level difference in mutation carriers and non−carriers with 99% confidence interval.
Fujirebio Aβ42/40 log2(pg/mL): Carrier − Non−Carrier
3
2
1
0
−1
−2
−3
-39.0
-25.0
-18.0
24.0
−30
−20
−10
0
10
20
Estimated Year of Onset (EYO)

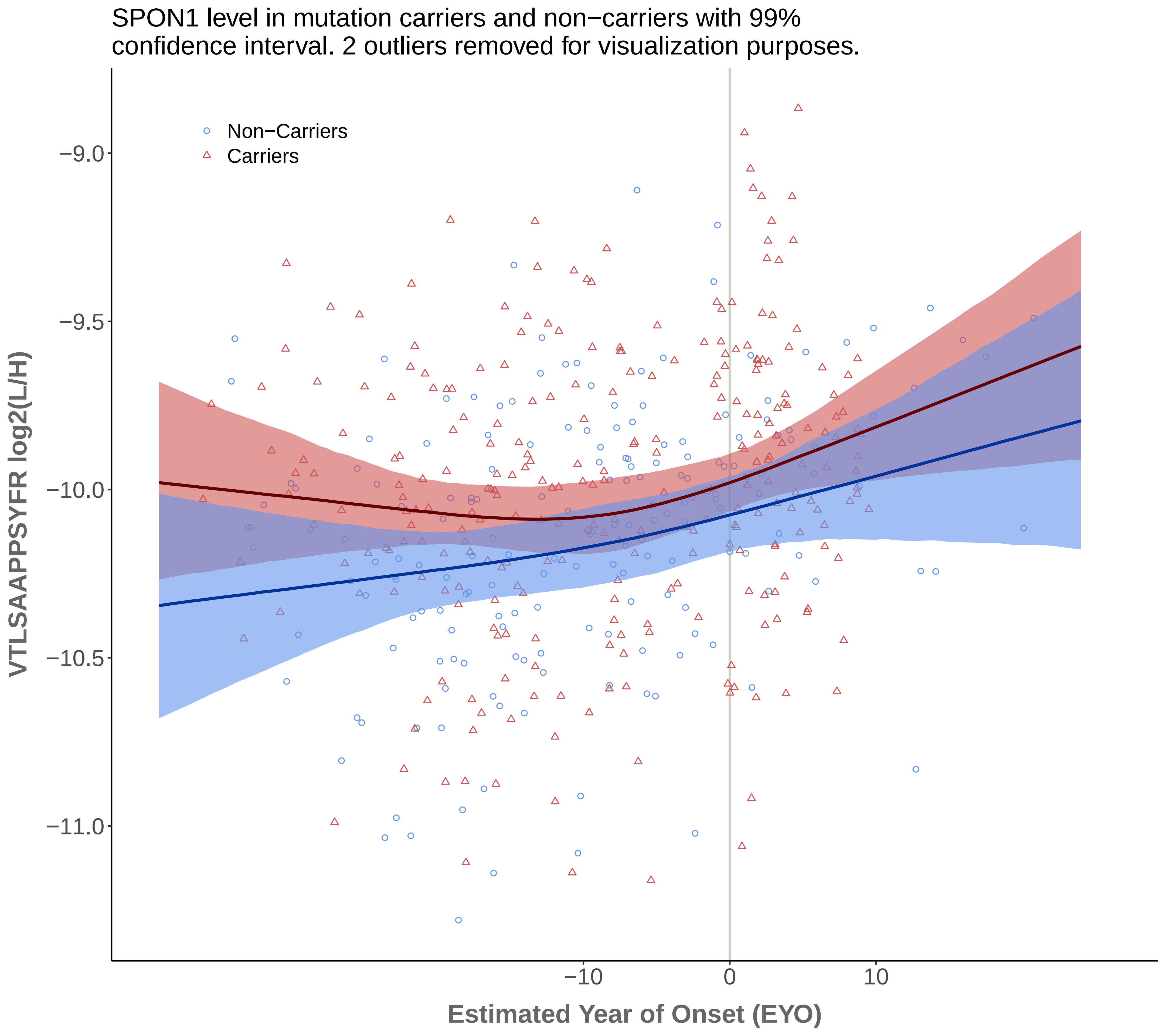

SPON1 level in mutation carriers and non−carriers with 99% confidence interval. 2 outliers removed for visualization purposes.
Non−Carriers
Carriers
VTLSAAPPSYFR log2(L/H)
Estimated Year of Onset (EYO)
−9.0
−9.5
−10.0
−10.5
−11.0
−10
0
10

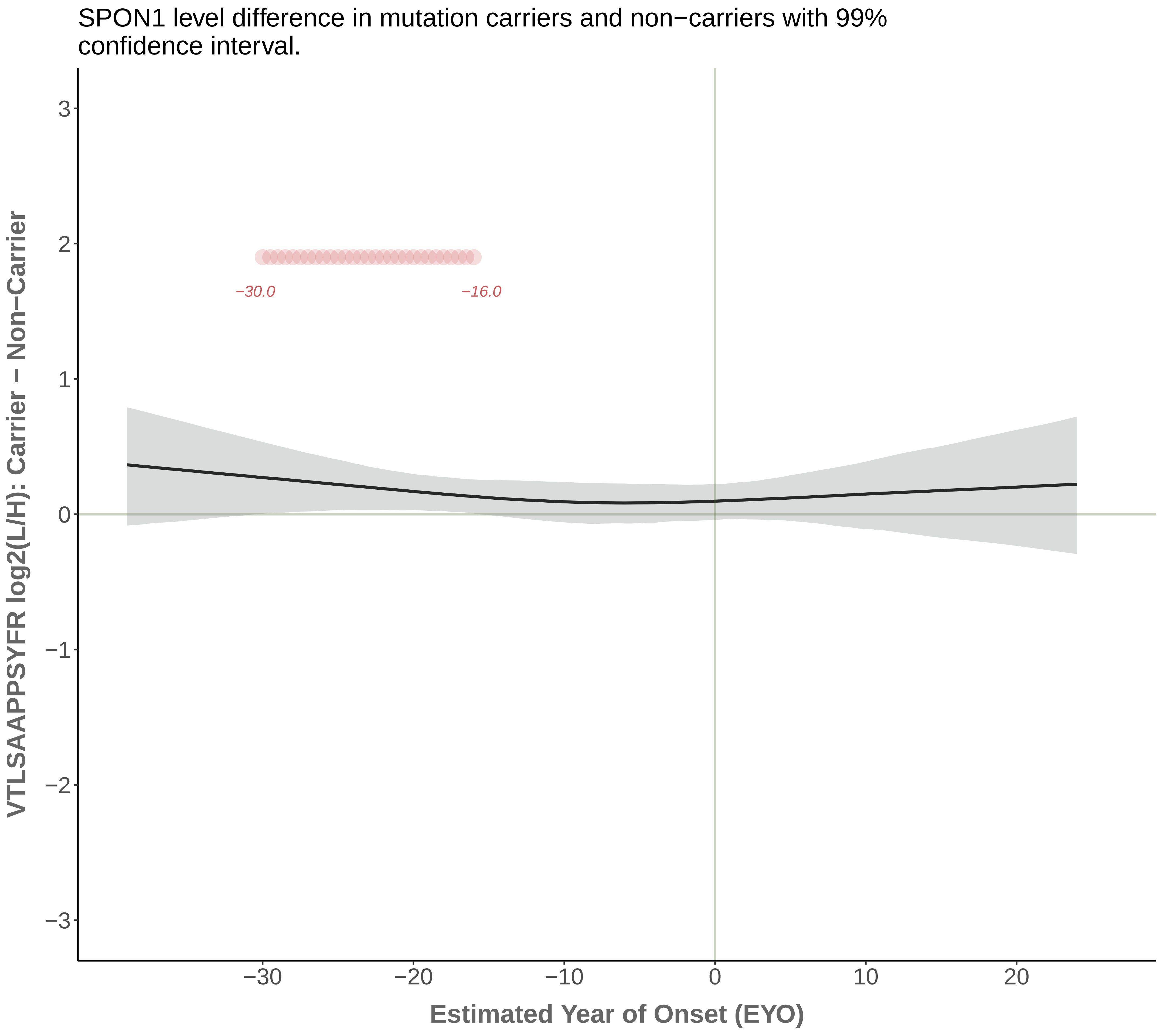

SPON1 level difference in mutation carriers and non−carriers with 99% confidence interval.
VTLSAAPPSYFR log2(L/H): Carrier − Non−Carrier
Estimated Year of Onset (EYO)
−30.0
−16.0
3
2
1
0
−1
−2
−3
−30
−20
−10
0
10
20

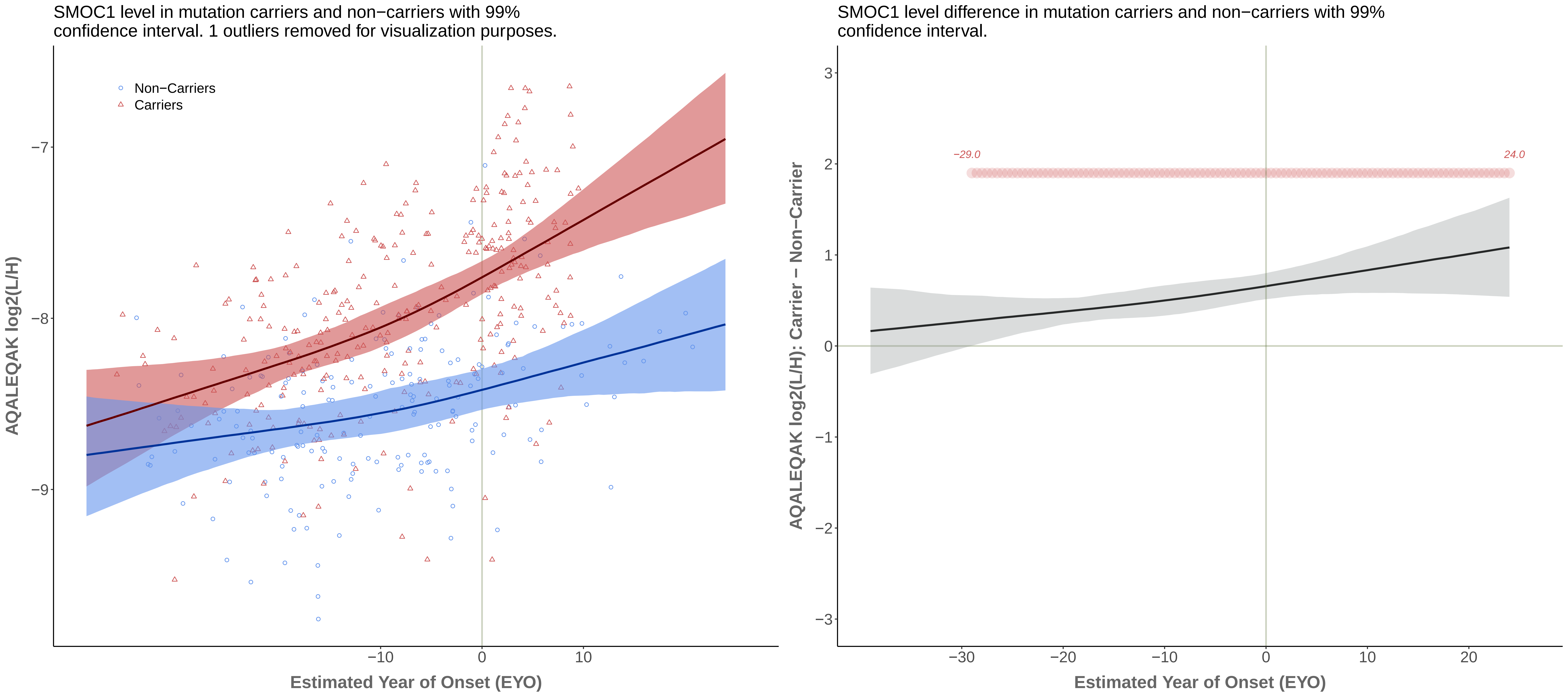
SMOC1 level in mutation carriers and non−carriers with 99% confidence interval. 1 outliers removed for visualization purposes.
Non−Carriers
Carriers
AQALEQAK log2(L/H)
−7
−8
−9
−10
0
10
Estimated Year of Onset (EYO)
SMOC1 level difference in mutation carriers and non−carriers with 99% confidence interval.
AQALEQAK log2(L/H): Carrier − Non−Carrier
−29.0
24.0
3
2
1
0
−1
−2
−3
−30
−20
−10
0
10
20
Estimated Year of Onset (EYO)

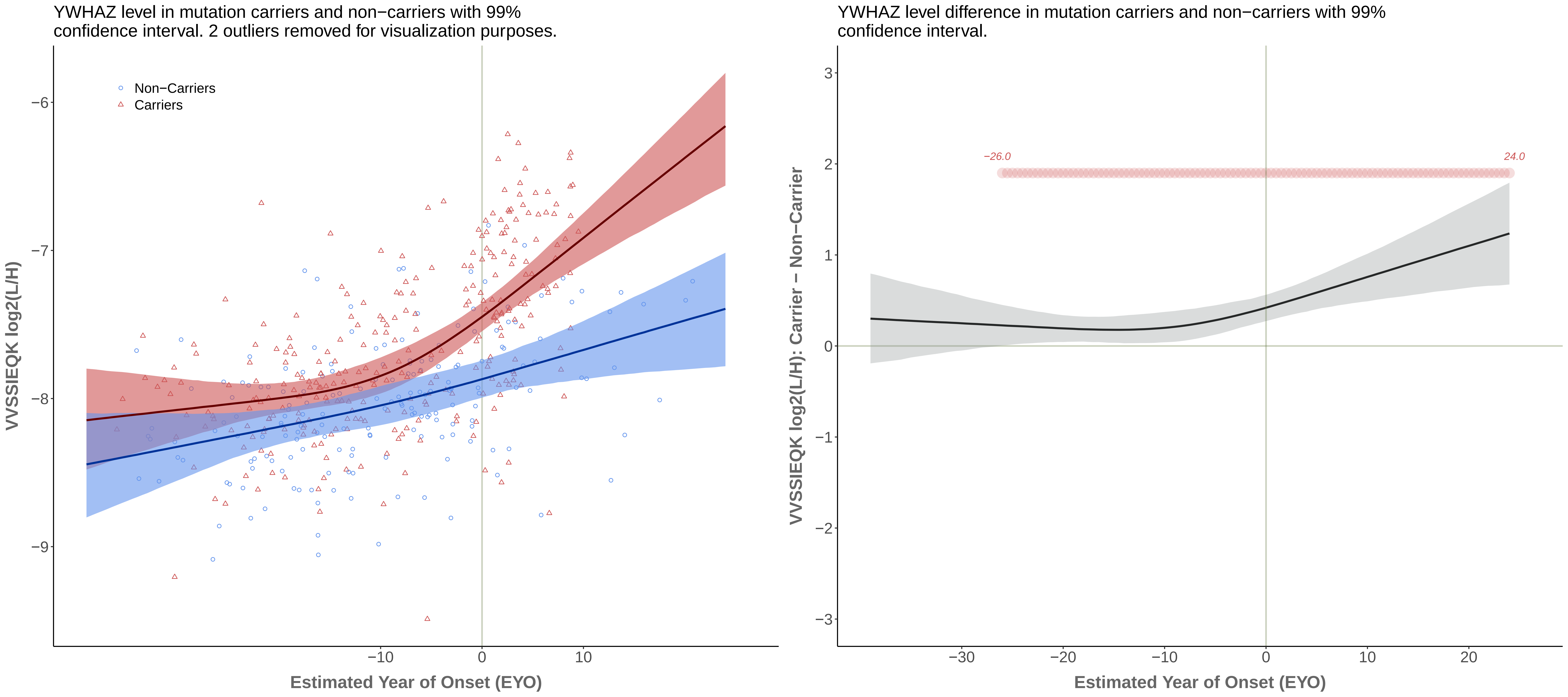
YWHAZ level in mutation carriers and non−carriers with 99% confidence interval. 2 outliers removed for visualization purposes.
Non−Carriers
Carriers
−6
−7
−8
−9
VVSSIEQK log2(L/H)
−10
0
10
Estimated Year of Onset (EYO)
YWHAZ level difference in mutation carriers and non−carriers with 99% confidence interval.
−26.0
24.0
3
2
1
0
−1
−2
−3
VVSSIEQK log2(L/H): Carrier − Non−Carrier
−30
−20
−10
0
10
20
Estimated Year of Onset (EYO)

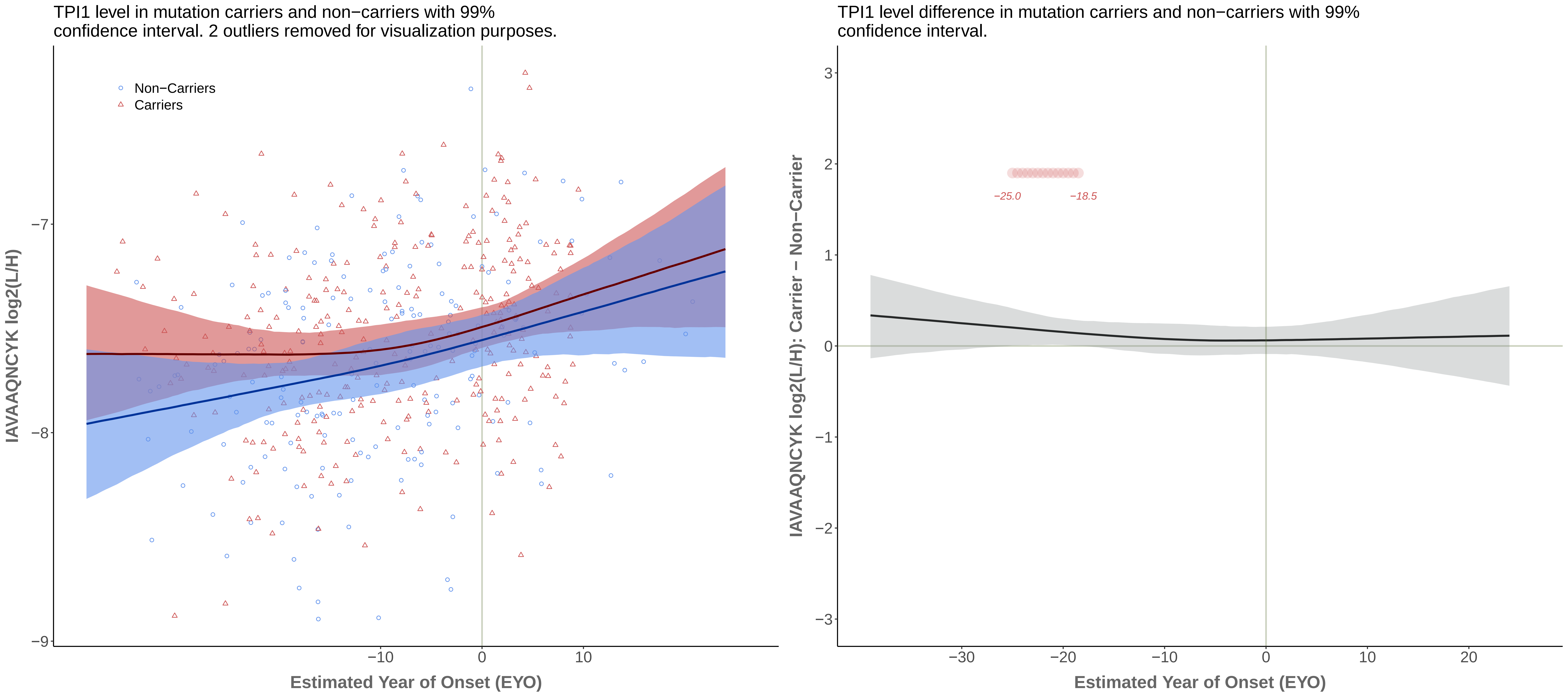
TPI1 level in mutation carriers and non−carriers with 99% confidence interval. 2 outliers removed for visualization purposes.
Non−Carriers
Carriers
IAVAAQNCYK log2(L/H)
−7
−8
−9
−10
0
10
Estimated Year of Onset (EYO)
TPI1 level difference in mutation carriers and non−carriers with 99% confidence interval.
IAVAAQNCYK log2(L/H): Carrier − Non−Carrier
3
2
1
0
−1
−2
−3
−25.0
−18.5
−30
−20
−10
0
10
20
Estimated Year of Onset (EYO)

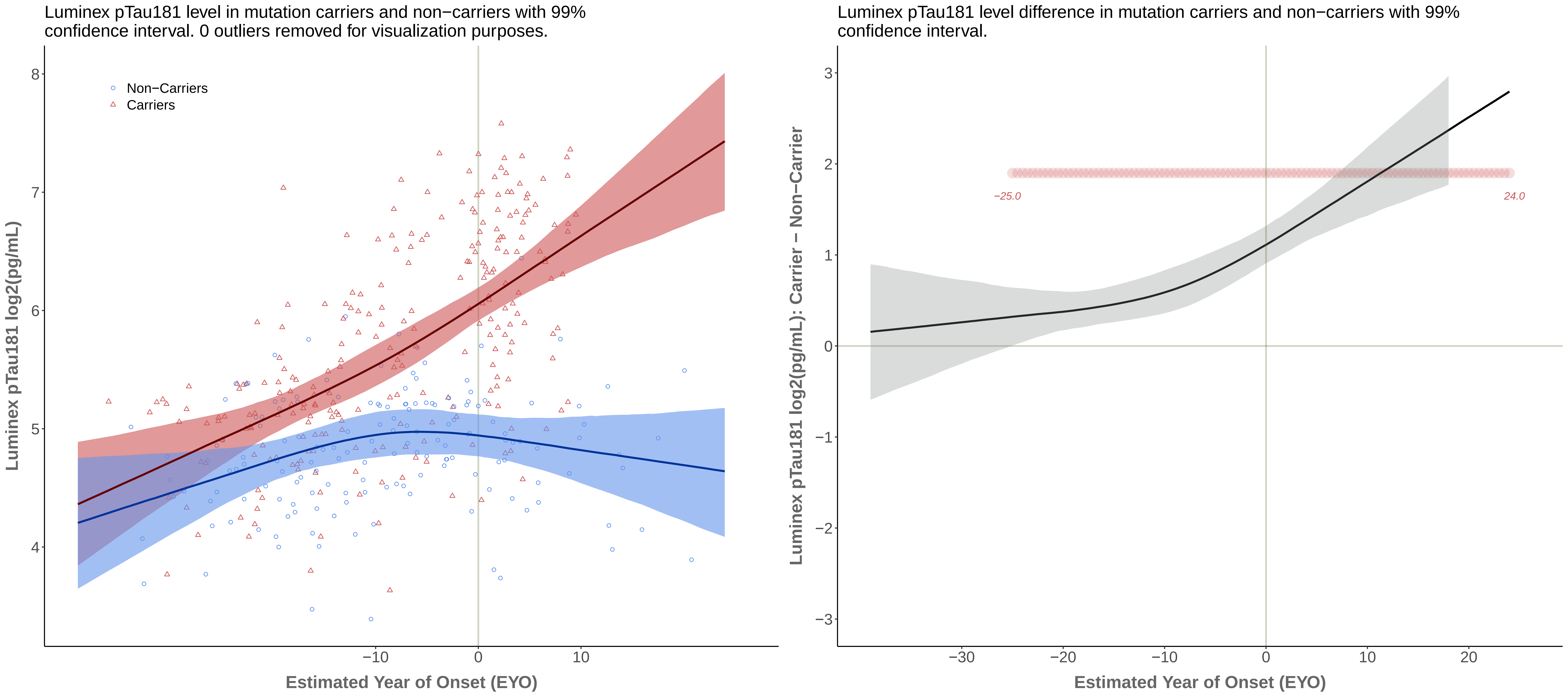

Luminex pTau181 level in mutation carriers and non−carriers with 99% confidence interval. 0 outliers removed for visualization purposes.
Non−Carriers
Carriers
Luminex pTau181 log2(pg/mL)
Estimated Year of Onset (EYO)
Luminex pTau181 level difference in mutation carriers and non−carriers with 99% confidence interval.
Luminex pTau181 log2(pg/mL): Carrier − Non−Carrier
−25.0
24.0
Estimated Year of Onset (EYO)

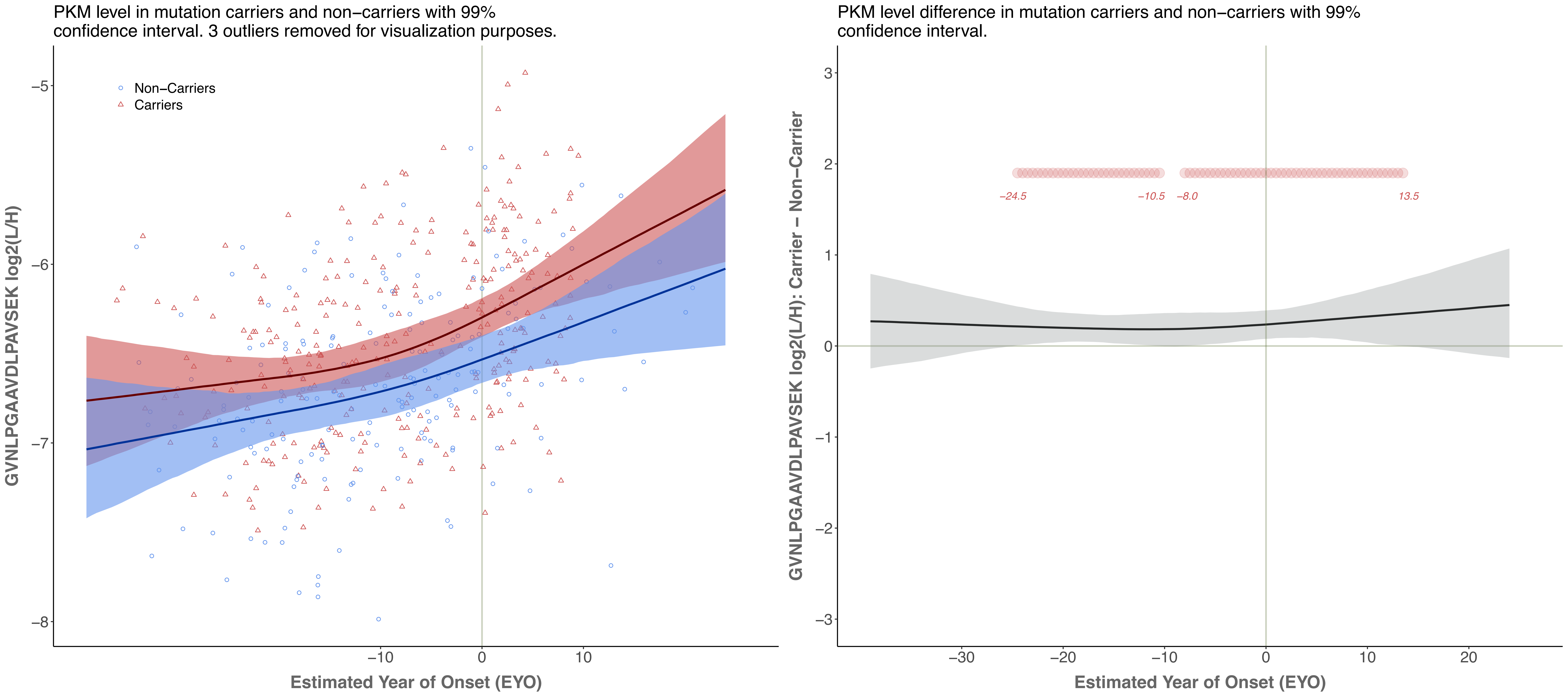

PKM level in mutation carriers and non−carriers with 99% confidence interval. 3 outliers removed for visualization purposes.
Non−Carriers
Carriers
GVNLPGAAVDLPAVSEK log2(L/H)
−5
−6
−7
−8
−10
0
10
Estimated Year of Onset (EYO)
PKM level difference in mutation carriers and non−carriers with 99% confidence interval.
GVNLPGAAVDLPAVSEK log2(L/H): Carrier − Non−Carrier
3
2
1
0
−1
−2
−3
−24.5
−10.5
−8.0
13.5
−30
−20
−10
0
10
20
Estimated Year of Onset (EYO)

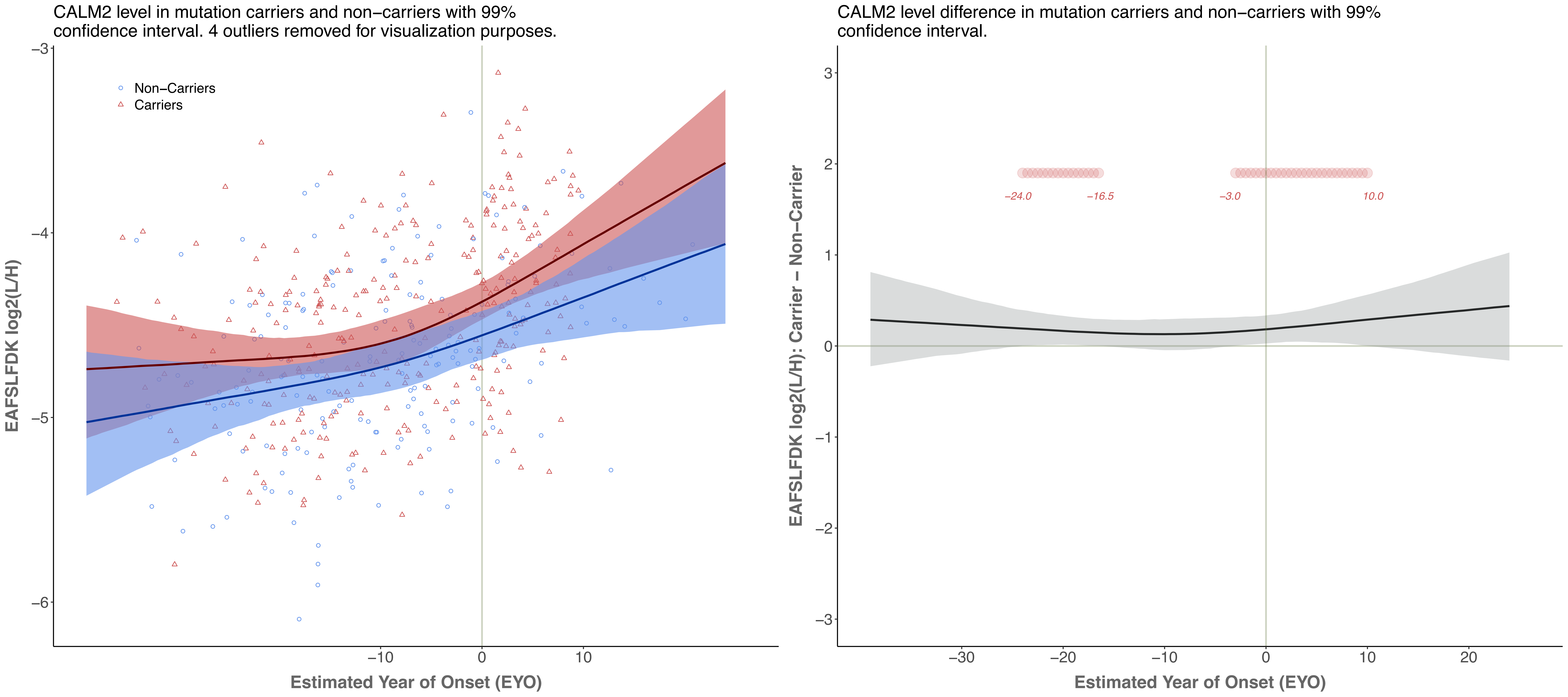
CALM2 level in mutation carriers and non−carriers with 99% confidence interval. 4 outliers removed for visualization purposes.
Non−Carriers
Carriers
EAFSLFDK log2(L/H)
Estimated Year of Onset (EYO)
−3
−4
−5
−6
−10
0
10
CALM2 level difference in mutation carriers and non−carriers with 99% confidence interval.
EAFSLFDK log2(L/H): Carrier − Non−Carrier
−24.0
−16.5
−3.0
10.0
3
2
1
0
−1
−2
−3
−30
−20
−10
0
10
20
Estimated Year of Onset (EYO)

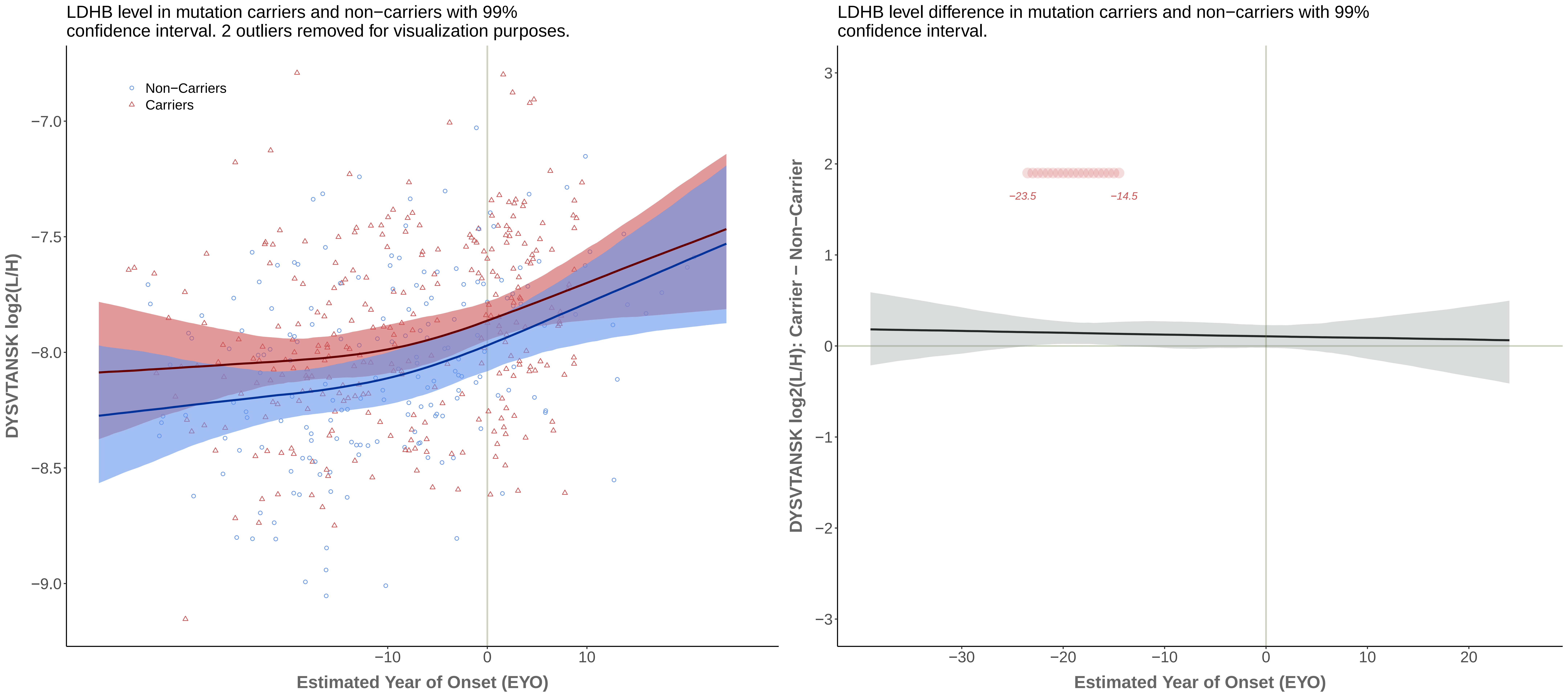

LDHB level in mutation carriers and non−carriers with 99% confidence interval. 2 outliers removed for visualization purposes.
Non−Carriers
Carriers
−7.0
−7.5
−8.0
−8.5
−9.0
DYSVTANSK log2(L/H)
−10
0
10
Estimated Year of Onset (EYO)
LDHB level difference in mutation carriers and non−carriers with 99% confidence interval.
−23.5
−14.5
3
2
1
0
−1
−2
−3
DYSVTANSK log2(L/H): Carrier − Non−Carrier
−30
−20
−10
0
10
20
Estimated Year of Onset (EYO)

GOT1 level in mutation carriers and non−carriers with 99% confidence interval. 4 outliers removed for visualization purposes.

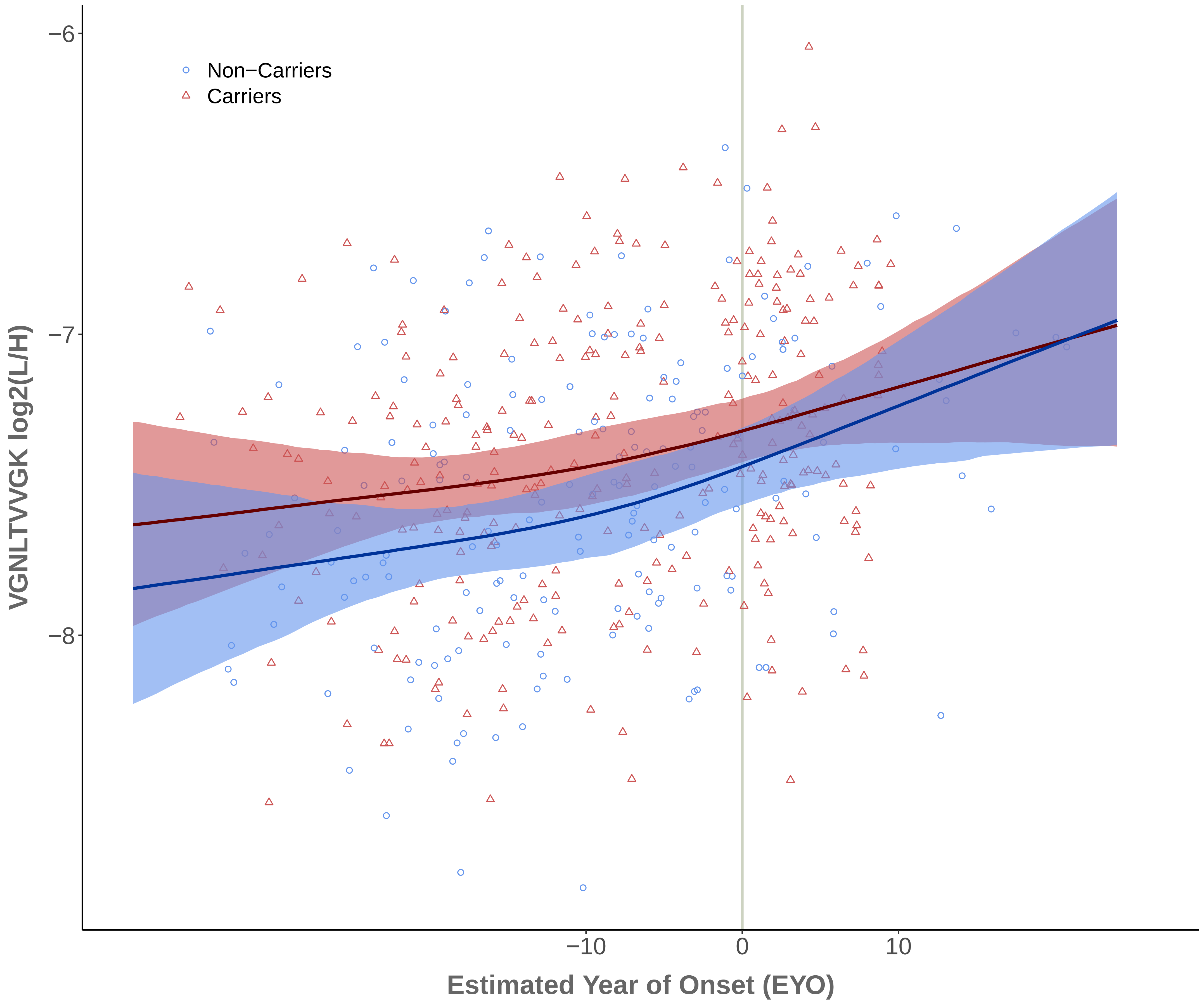

GOT1 level difference in mutation carriers and non−carriers with 99% confidence interval.

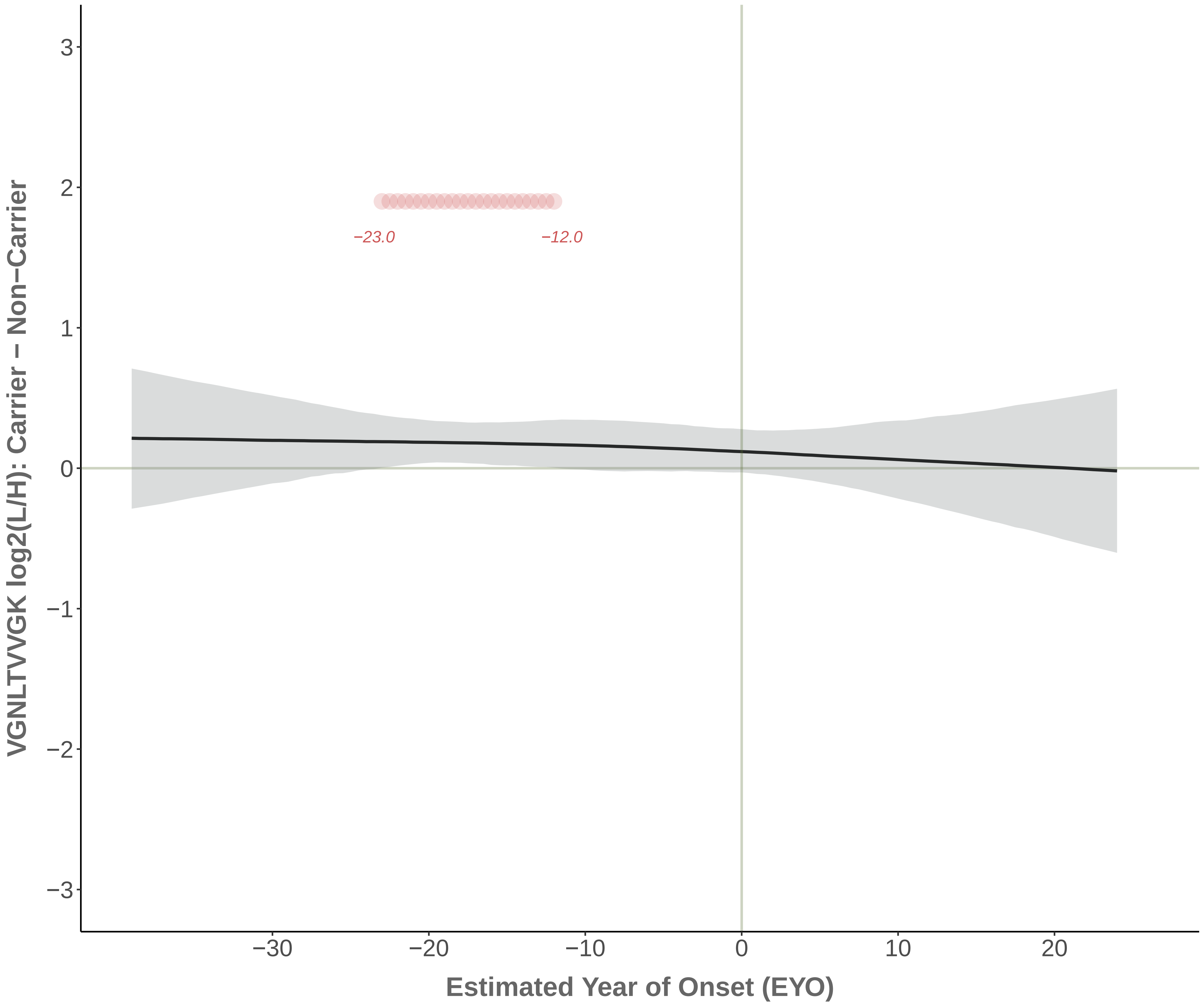

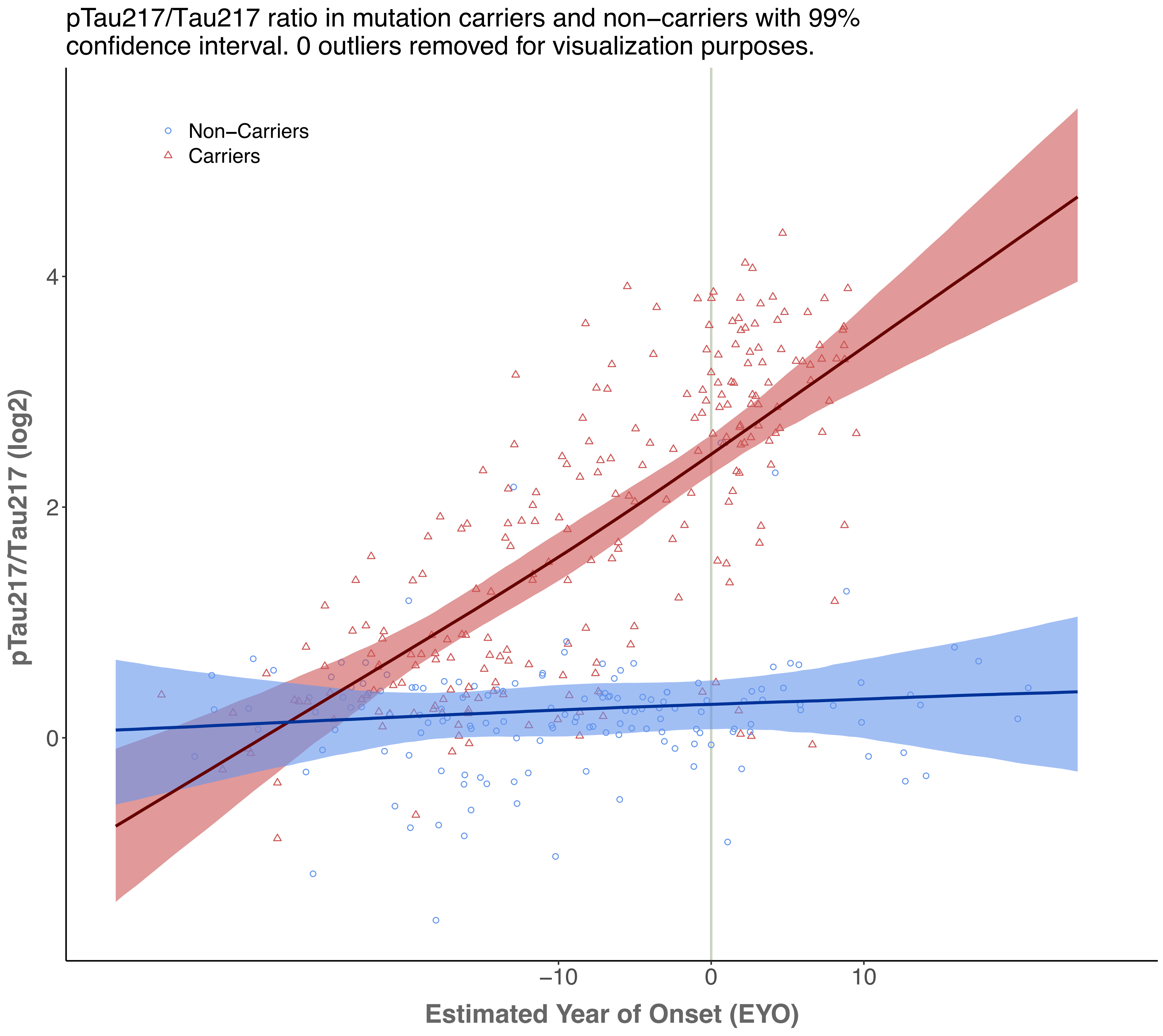
pTau217/Tau217 ratio in mutation carriers and non−carriers with 99% confidence interval. 0 outliers removed for visualization purposes.
Non−Carriers
Carriers
pTau217/Tau217 (log2)
Estimated Year of Onset (EYO)
−10
0
10
0
2
4

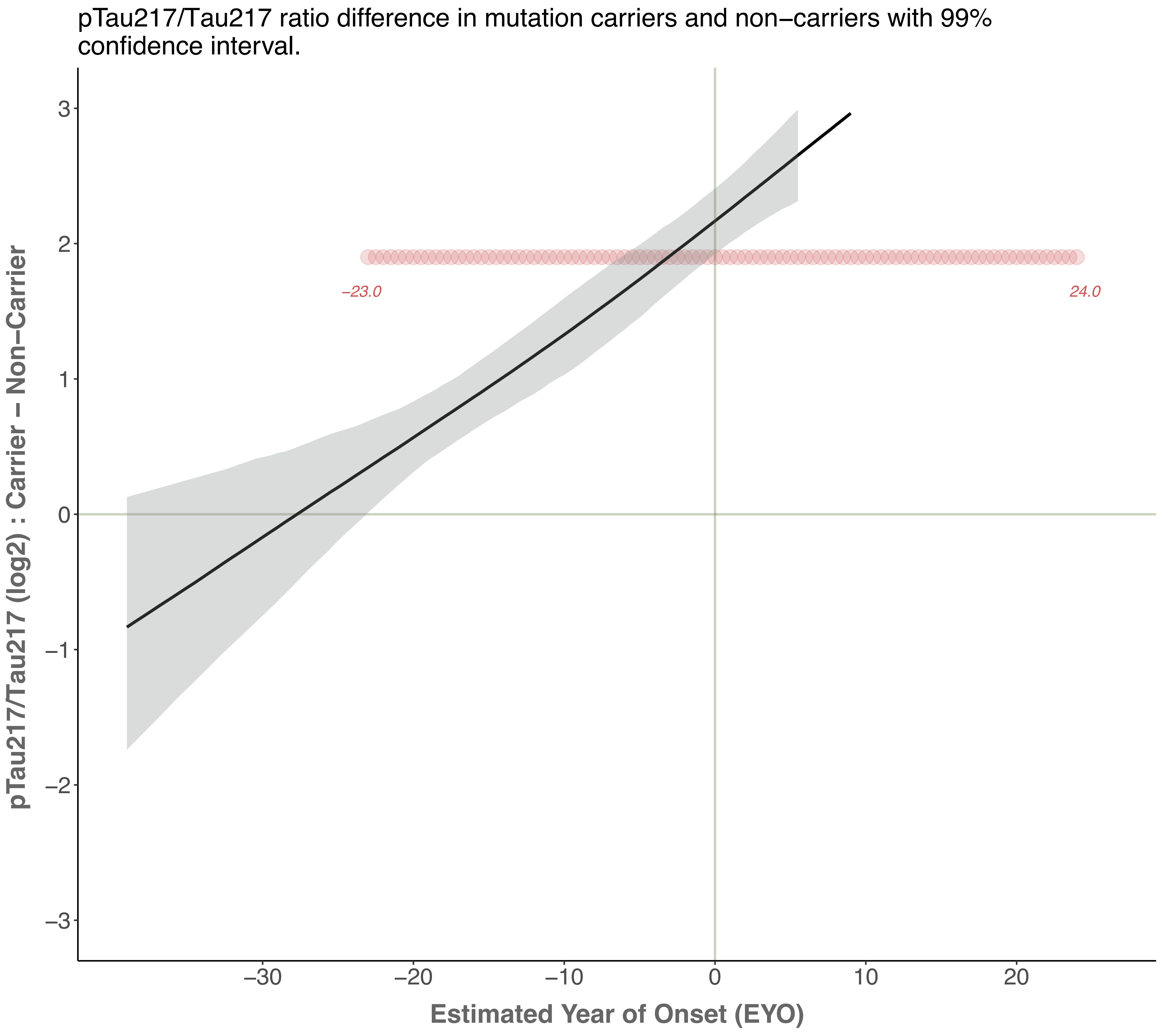
pTau217/Tau217 ratio difference in mutation carriers and non−carriers with 99% confidence interval.
pTau217/Tau217 (log2) : Carrier − Non−Carrier
Estimated Year of Onset (EYO)
−23.0
24.0
−30
−20
−10
0
10
20
3
2
1
0
−1
−2
−3

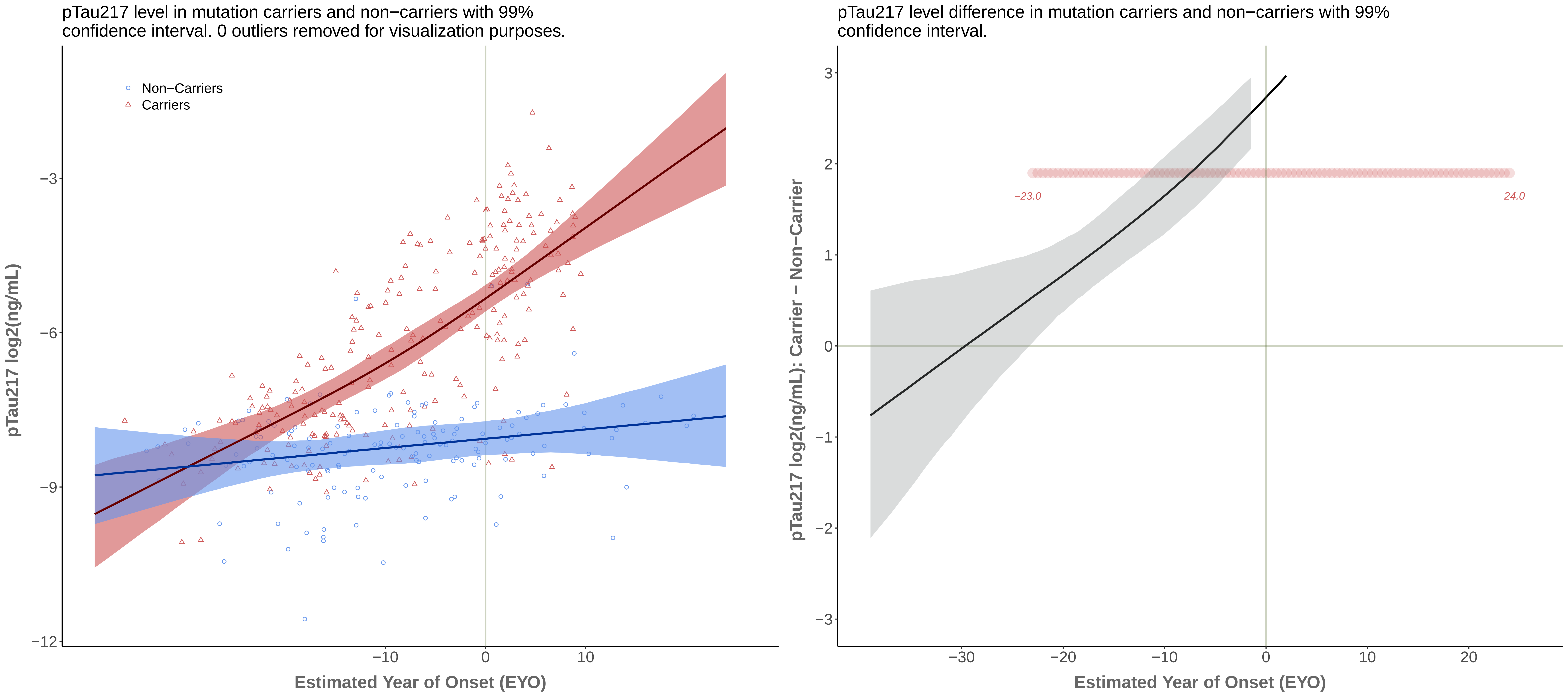
pTau217 level in mutation carriers and non−carriers with 99% confidence interval. 0 outliers removed for visualization purposes.
Non−Carriers
Carriers
pTau217 log2(ng/mL)
−3
−6
−9
−12
−10
0
10
Estimated Year of Onset (EYO)
pTau217 level difference in mutation carriers and non−carriers with 99% confidence interval.
pTau217 log2(ng/mL): Carrier − Non−Carrier
3
2
1
0
−1
−2
−3
−23.0
24.0
−30
−20
−10
0
10
20
Estimated Year of Onset (EYO)

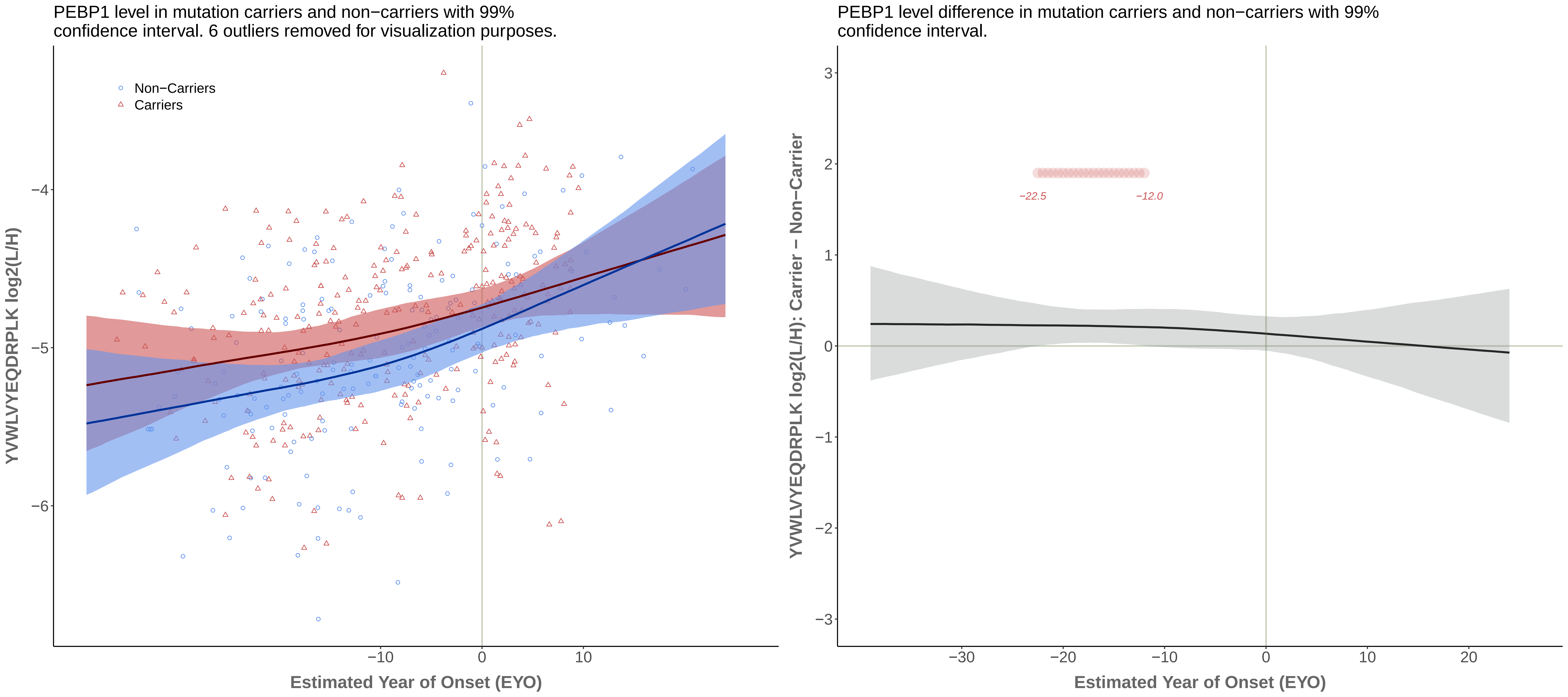

PEBP1 level in mutation carriers and non−carriers with 99% confidence interval. 6 outliers removed for visualization purposes.
Non−Carriers
Carriers
YVWLVYEQDRPLK log2(L/H)
−4
−5
−6
−10
0
10
Estimated Year of Onset (EYO)
PEBP1 level difference in mutation carriers and non−carriers with 99% confidence interval.
YVWLVYEQDRPLK log2(L/H): Carrier − Non−Carrier
3
2
1
0
−1
−2
−3
−22.5
−12.0
−30
−20
−10
0
10
20
Estimated Year of Onset (EYO)

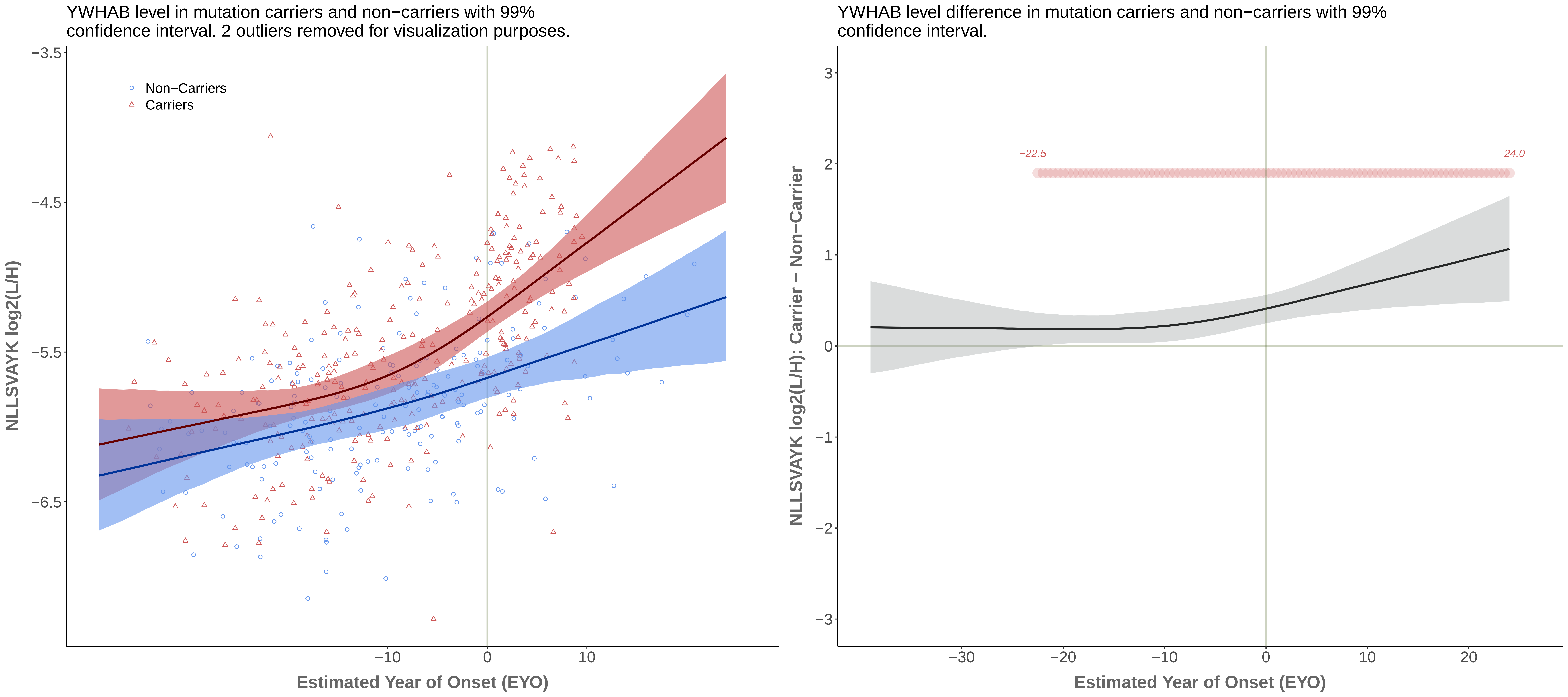

YWHAB level in mutation carriers and non−carriers with 99% confidence interval. 2 outliers removed for visualization purposes.
Non−Carriers
Carriers
−3.5
−4.5
−5.5
−6.5
NLLSVAYK log2(L/H)
−10
0
10
Estimated Year of Onset (EYO)
YWHAB level difference in mutation carriers and non−carriers with 99% confidence interval.
−22.5
24.0
3
2
1
0
−1
−2
−3
NLLSVAYK log2(L/H): Carrier − Non−Carrier
−30
−20
−10
0
10
20
Estimated Year of Onset (EYO)

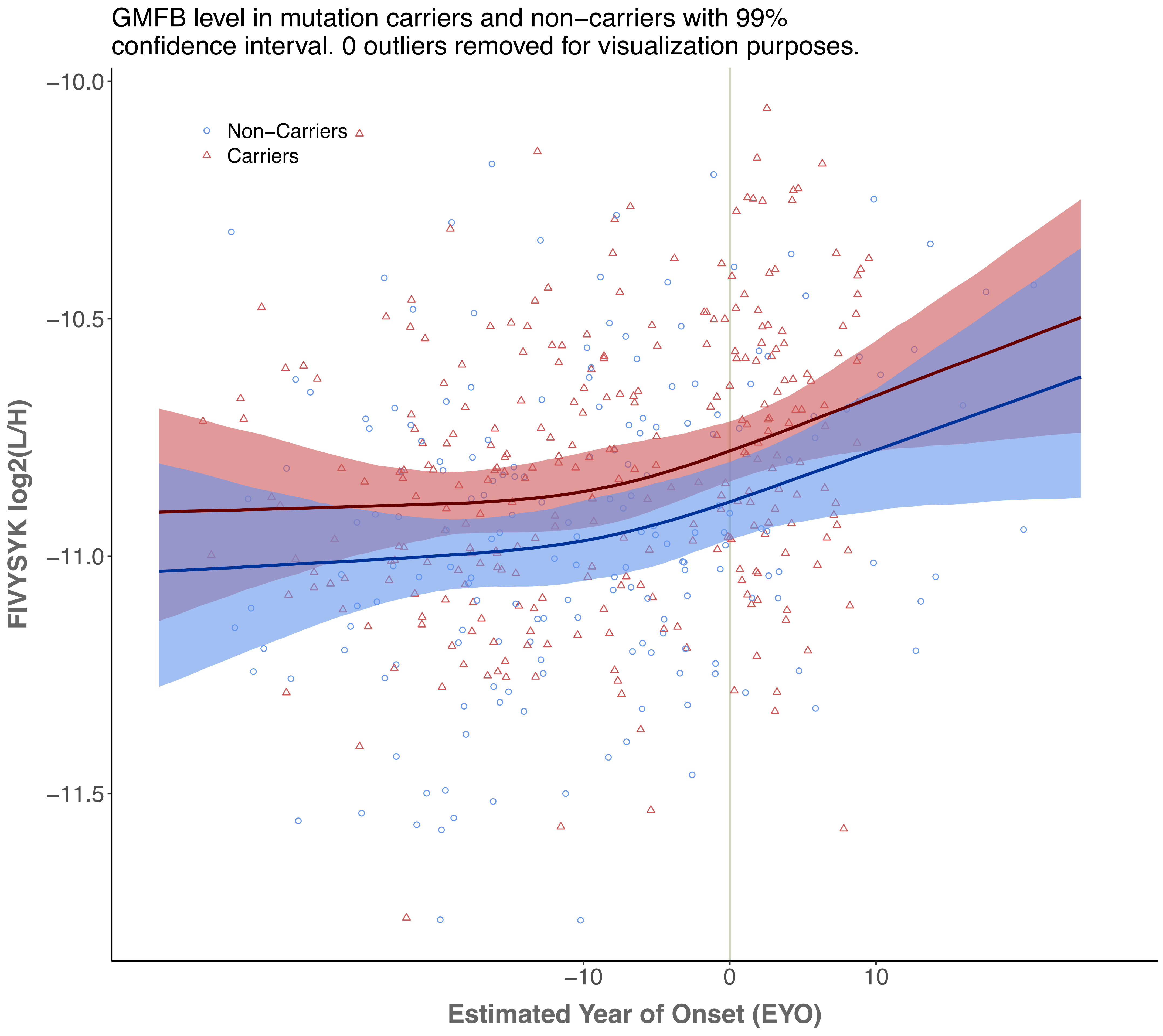
GMFB level in mutation carriers and non−carriers with 99%
confidence interval. 0 outliers removed for visualization purposes.
Non−Carriers
Carriers
FIVYSYK log2(L/H)
−10.0
−10.5
−11.0
−11.5
−10
0
10
Estimated Year of Onset (EYO)

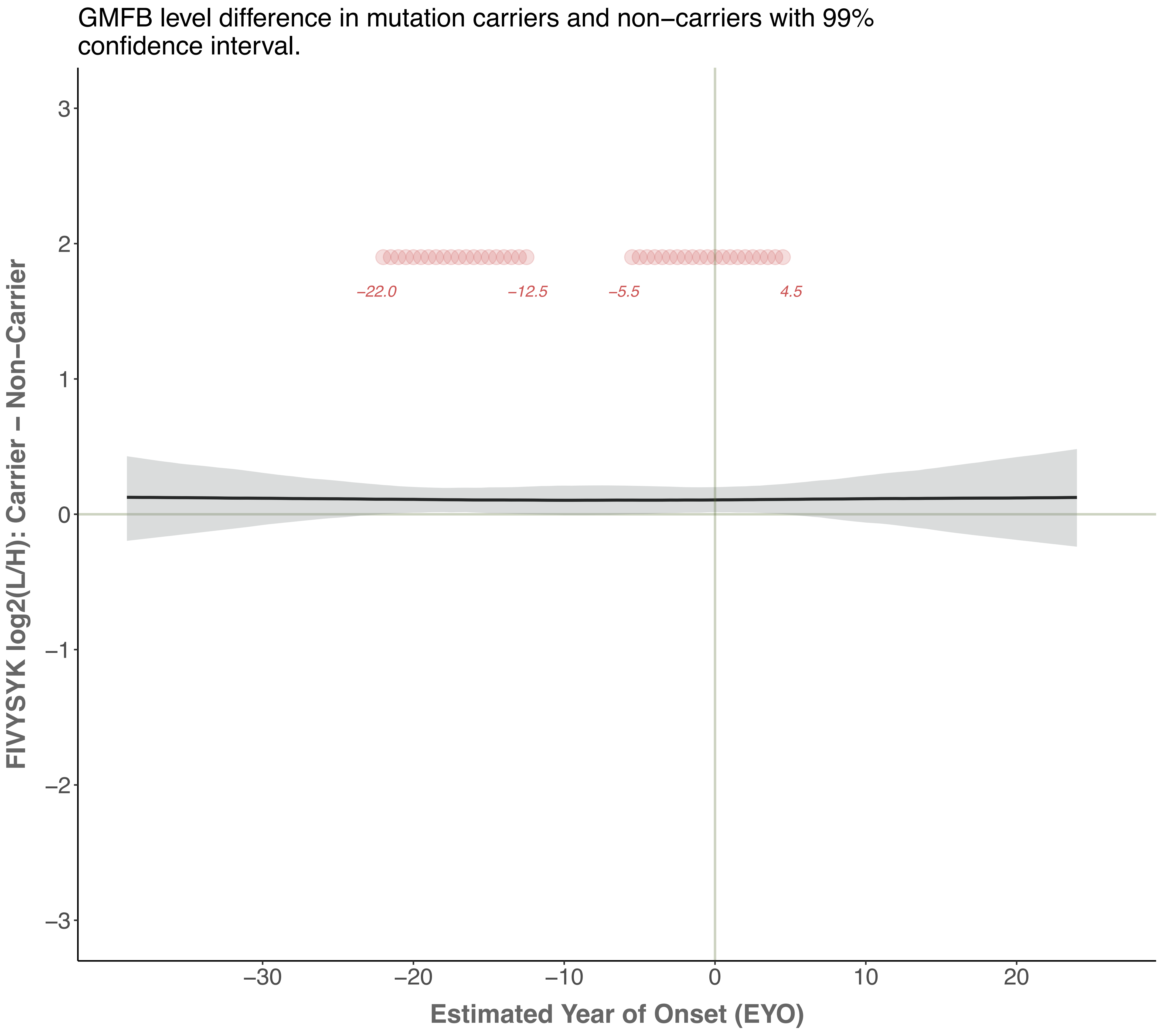
GMFB level difference in mutation carriers and non−carriers with 99%
confidence interval.
−22.0
−12.5
−5.5
4.5
FIVYSYK log2(L/H): Carrier − Non−Carrier
3
2
1
0
−1
−2
−3
−30
−20
−10
0
10
20
Estimated Year of Onset (EYO)

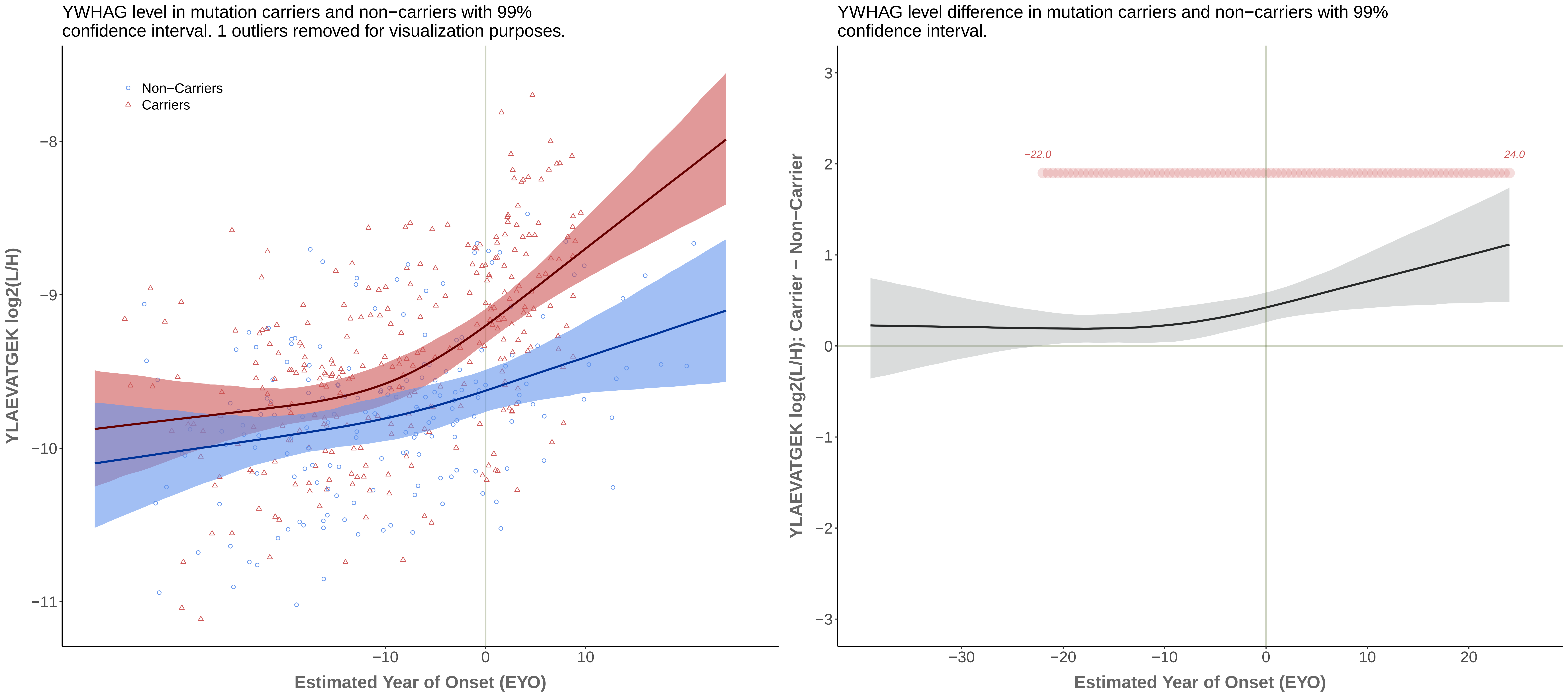
YWHAG level in mutation carriers and non−carriers with 99% confidence interval. 1 outliers removed for visualization purposes.
Non−Carriers
Carriers
YLAEVATGEK log2(L/H)
Estimated Year of Onset (EYO)
YWHAG level difference in mutation carriers and non−carriers with 99% confidence interval.
−22.0
24.0
YLAEVATGEK log2(L/H): Carrier − Non−Carrier
Estimated Year of Onset (EYO)

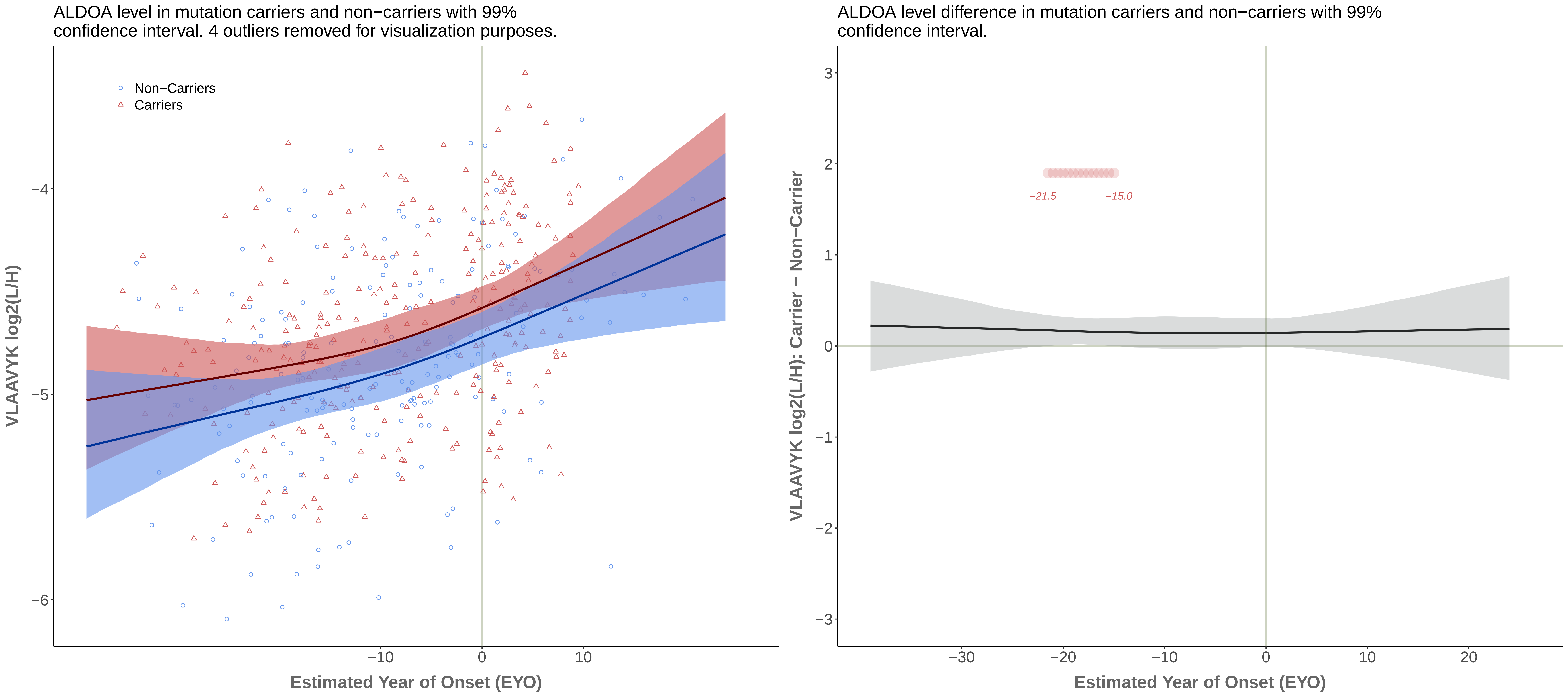
ALDOA level in mutation carriers and non−carriers with 99% confidence interval. 4 outliers removed for visualization purposes.
Non−Carriers
Carriers
−4
−5
−6
VLAAVYK log2(L/H)
−10
0
10
Estimated Year of Onset (EYO)
ALDOA level difference in mutation carriers and non−carriers with 99% confidence interval.
3
2
1
0
−1
−2
−3
−21.5
−15.0
VLAAVYK log2(L/H): Carrier − Non−Carrier
−30
−20
−10
0
10
20
Estimated Year of Onset (EYO)

PGAM1 level in mutation carriers and non−carriers with 99% confidence interval. 2 outliers removed for visualization purposes.

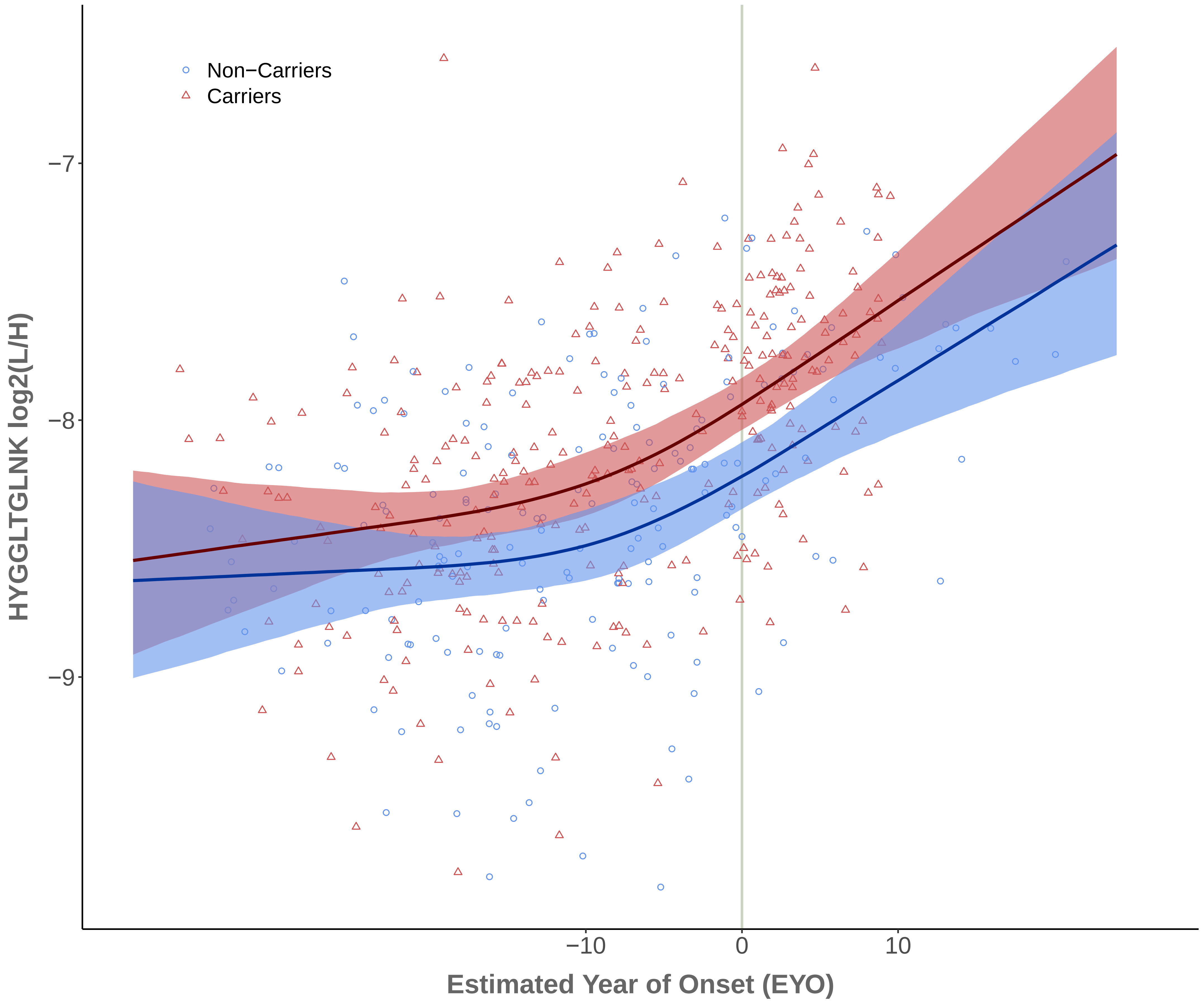

PGAM1 level difference in mutation carriers and non−carriers with 99% confidence interval.

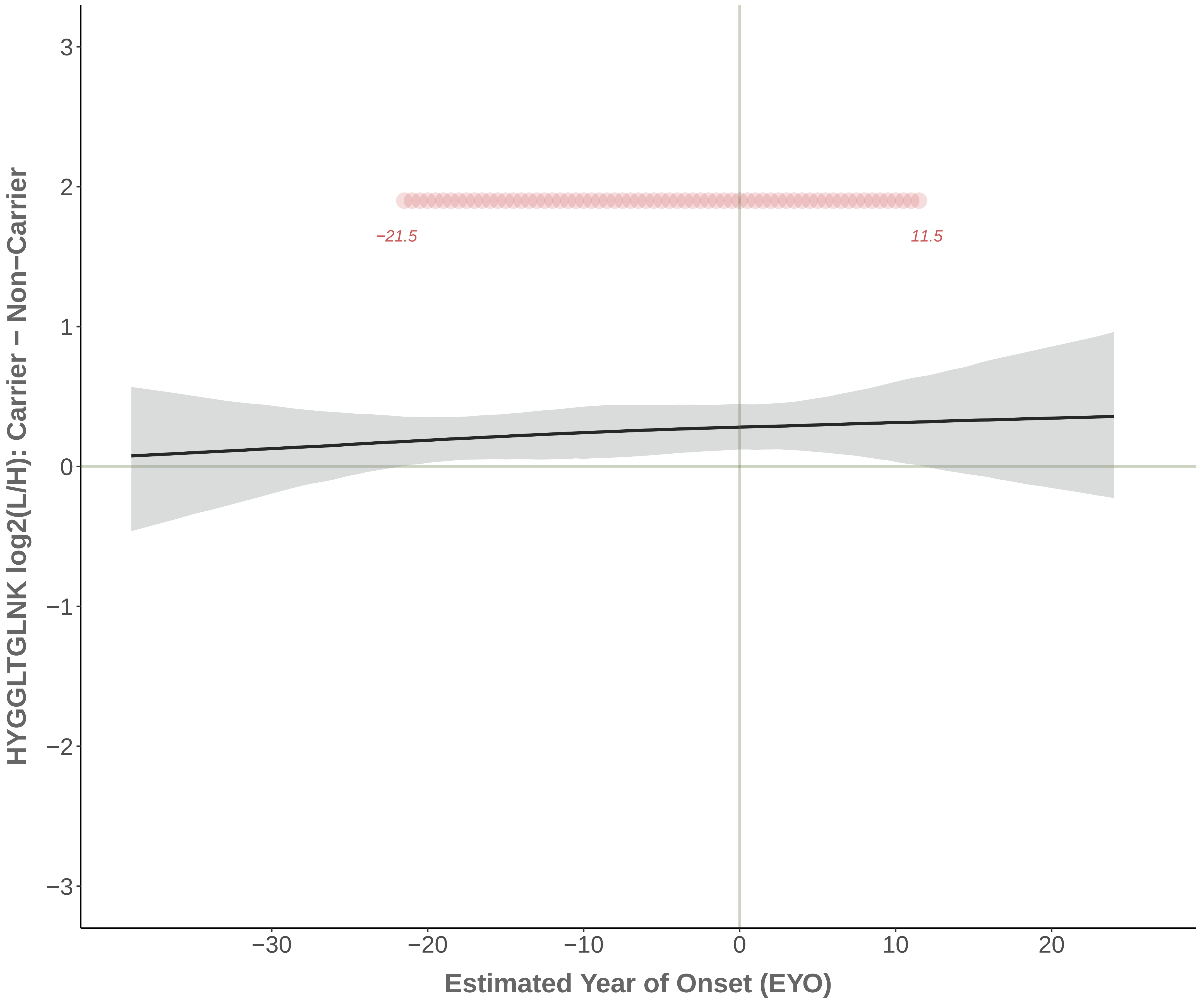

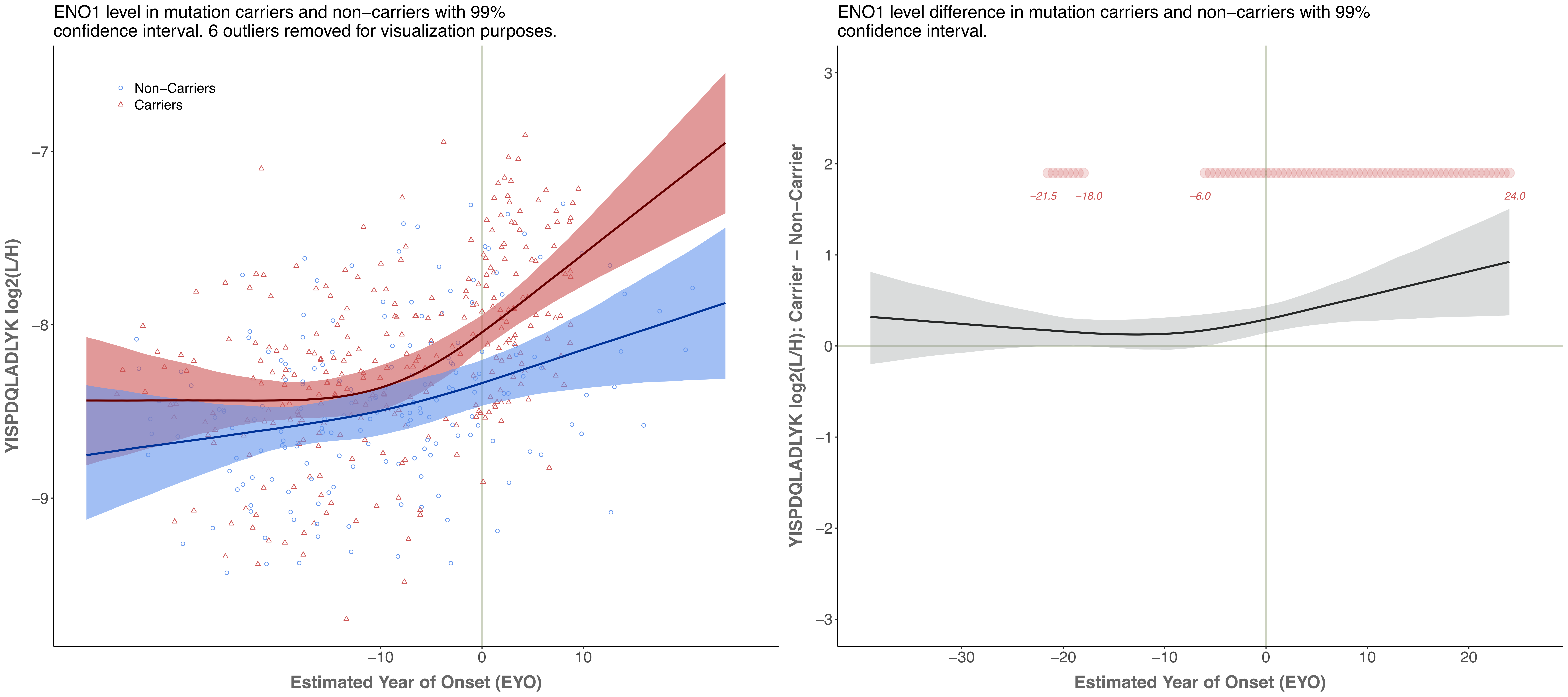

ENO1 level in mutation carriers and non−carriers with 99% confidence interval. 6 outliers removed for visualization purposes.
Non−Carriers
Carriers
−7
−8
−9
YISPDQLADLYK log2(L/H)
−10
0
10
Estimated Year of Onset (EYO)
ENO1 level difference in mutation carriers and non−carriers with 99% confidence interval.
3
2
1
0
−1
−2
−3
YISPDQLADLYK log2(L/H): Carrier − Non−Carrier
−21.5
−18.0
−6.0
24.0
−30
−20
−10
0
10
20
Estimated Year of Onset (EYO)

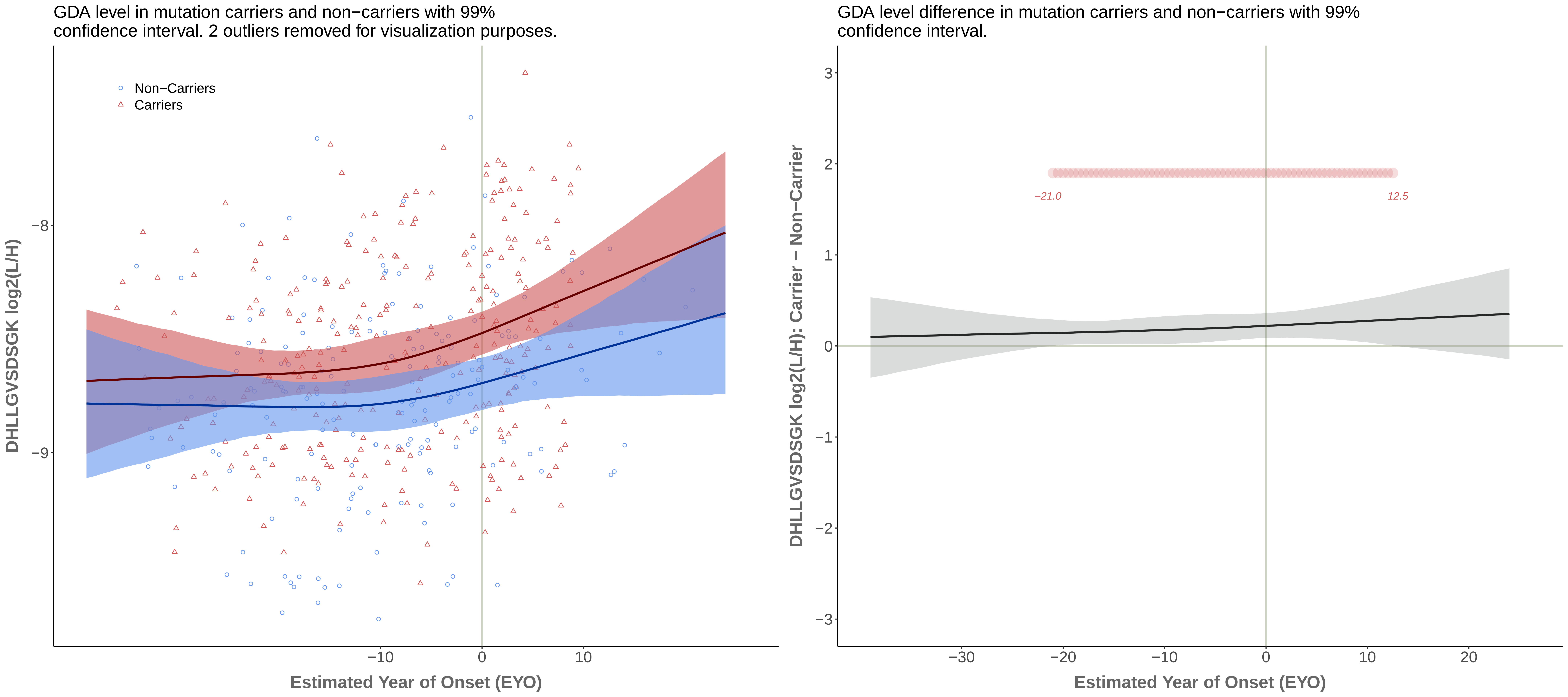

GDA level in mutation carriers and non−carriers with 99% confidence interval. 2 outliers removed for visualization purposes.
Non−Carriers
Carriers
DHLLGVSDSGK log2(L/H)
−8
−9
−10
0
10
Estimated Year of Onset (EYO)
GDA level difference in mutation carriers and non−carriers with 99% confidence interval.
DHLLGVSDSGK log2(L/H): Carrier − Non−Carrier
3
2
1
0
−1
−2
−3
−21.0
12.5
−30
−20
−10
0
10
20
Estimated Year of Onset (EYO)

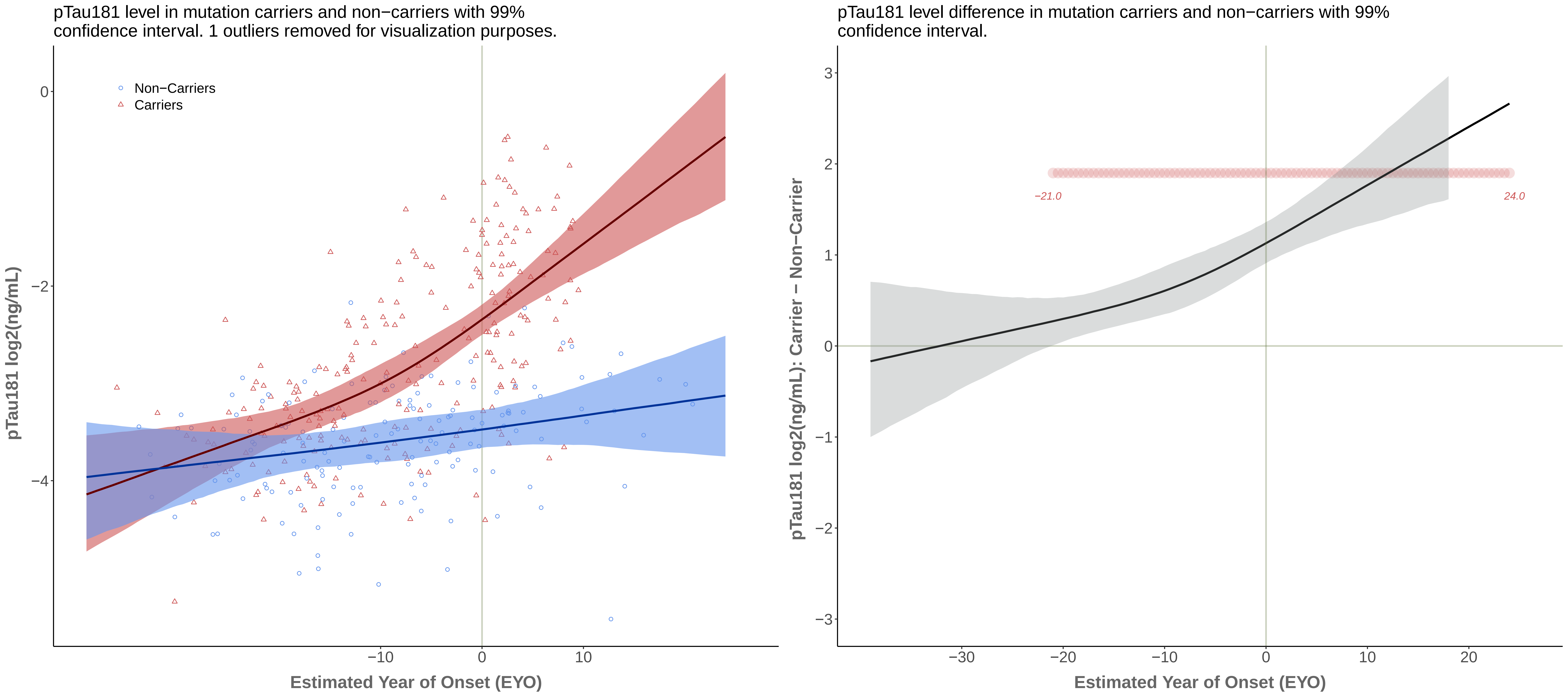

pTau181 level in mutation carriers and non−carriers with 99% confidence interval. 1 outliers removed for visualization purposes.
Non−Carriers
Carriers
pTau181 log2(ng/mL)
Estimated Year of Onset (EYO)
0
−2
−4
−10
0
10
pTau181 level difference in mutation carriers and non−carriers with 99% confidence interval.
pTau181 log2(ng/mL): Carrier − Non−Carrier
−21.0
24.0
3
2
1
0
−1
−2
−3
−30
−20
−10
0
10
20
Estimated Year of Onset (EYO)

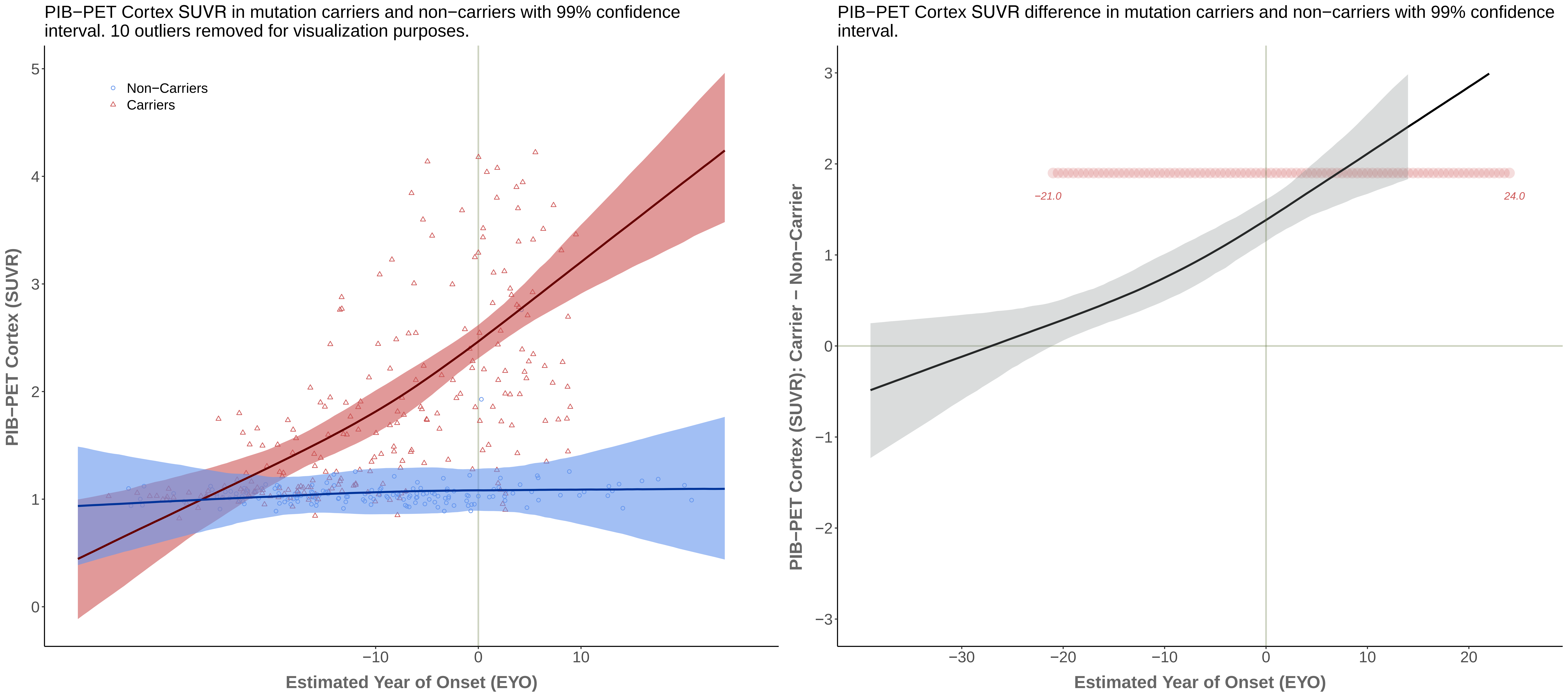
PIB−PET Cortex SUVR in mutation carriers and non−carriers with 99% confidence interval. 10 outliers removed for visualization purposes.
Non−Carriers
Carriers
PIB−PET Cortex (SUVR)
Estimated Year of Onset (EYO)
PIB−PET Cortex SUVR difference in mutation carriers and non−carriers with 99% confidence interval.
PIB−PET Cortex (SUVR): Carrier − Non−Carrier
−21.0
24.0
Estimated Year of Onset (EYO)

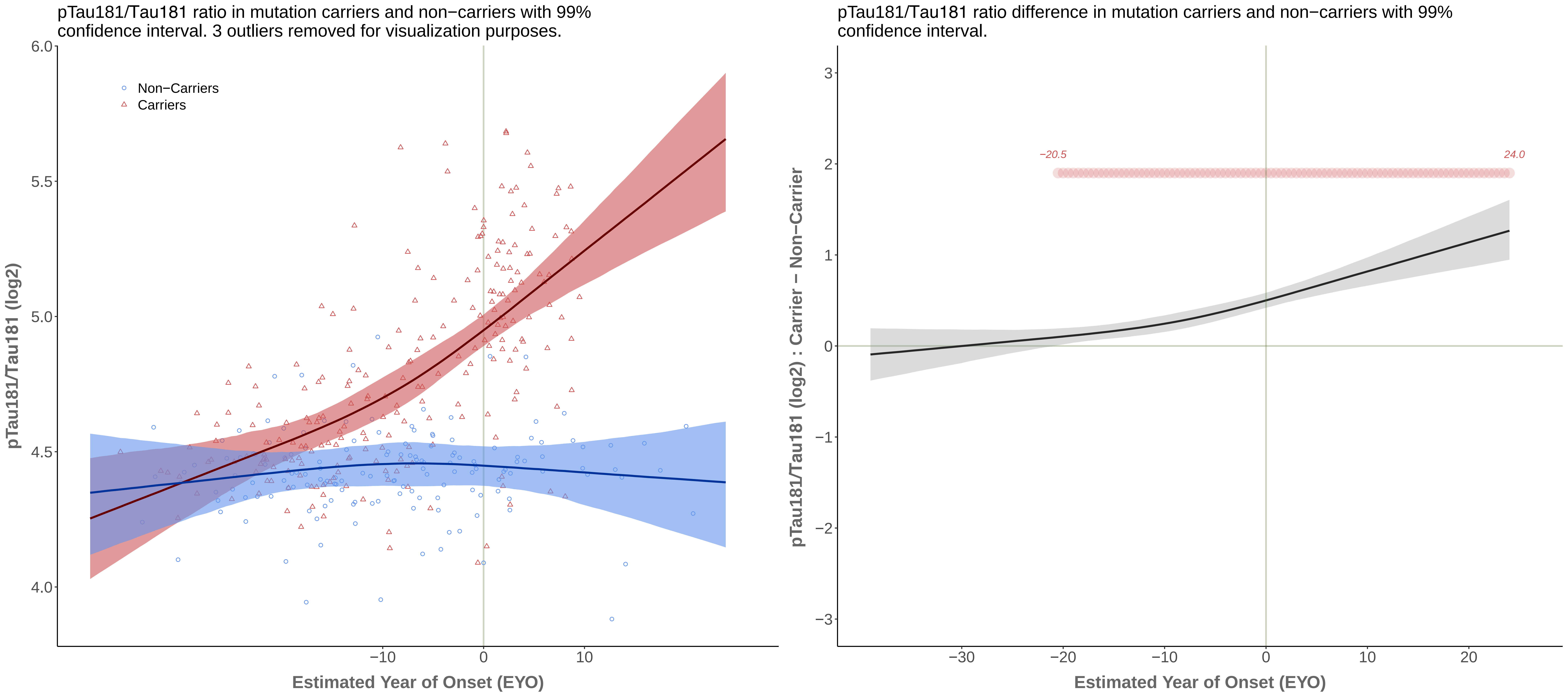

pTau181/Tau181 ratio in mutation carriers and non−carriers with 99% confidence interval. 3 outliers removed for visualization purposes.
Non−Carriers
Carriers
pTau181/Tau181 (log2)
Estimated Year of Onset (EYO)
pTau181/Tau181 ratio difference in mutation carriers and non−carriers with 99% confidence interval.
−20.5
24.0
pTau181/Tau181 (log2) : Carrier − Non−Carrier
Estimated Year of Onset (EYO)

Fujirebio pTau181 level in mutation carriers and non−carriers with 99% confidence interval. 0 outliers removed for visualization purposes.

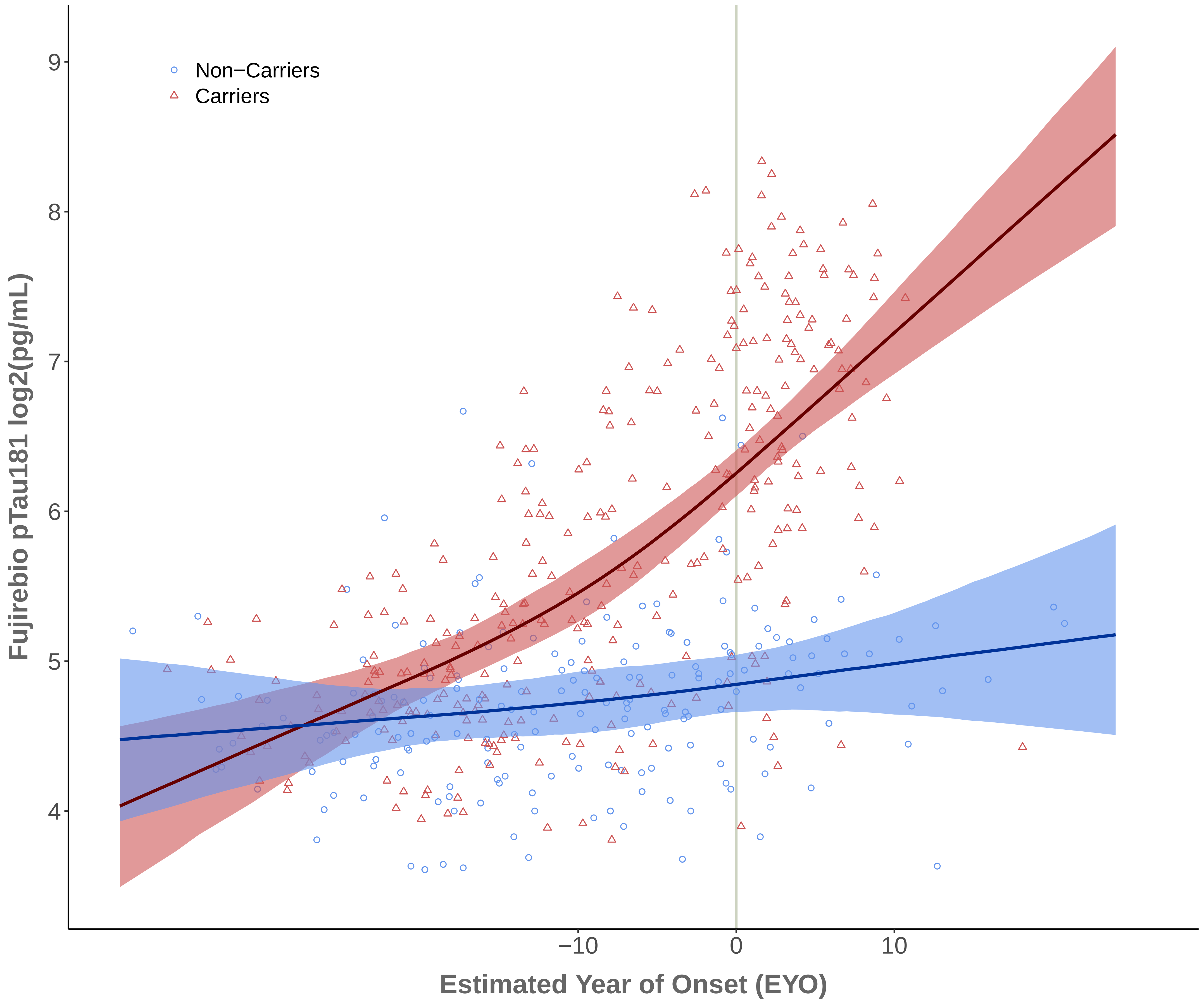

Fujirebio pTau181 level difference in mutation carriers and non−carriers with 99% confidence interval.

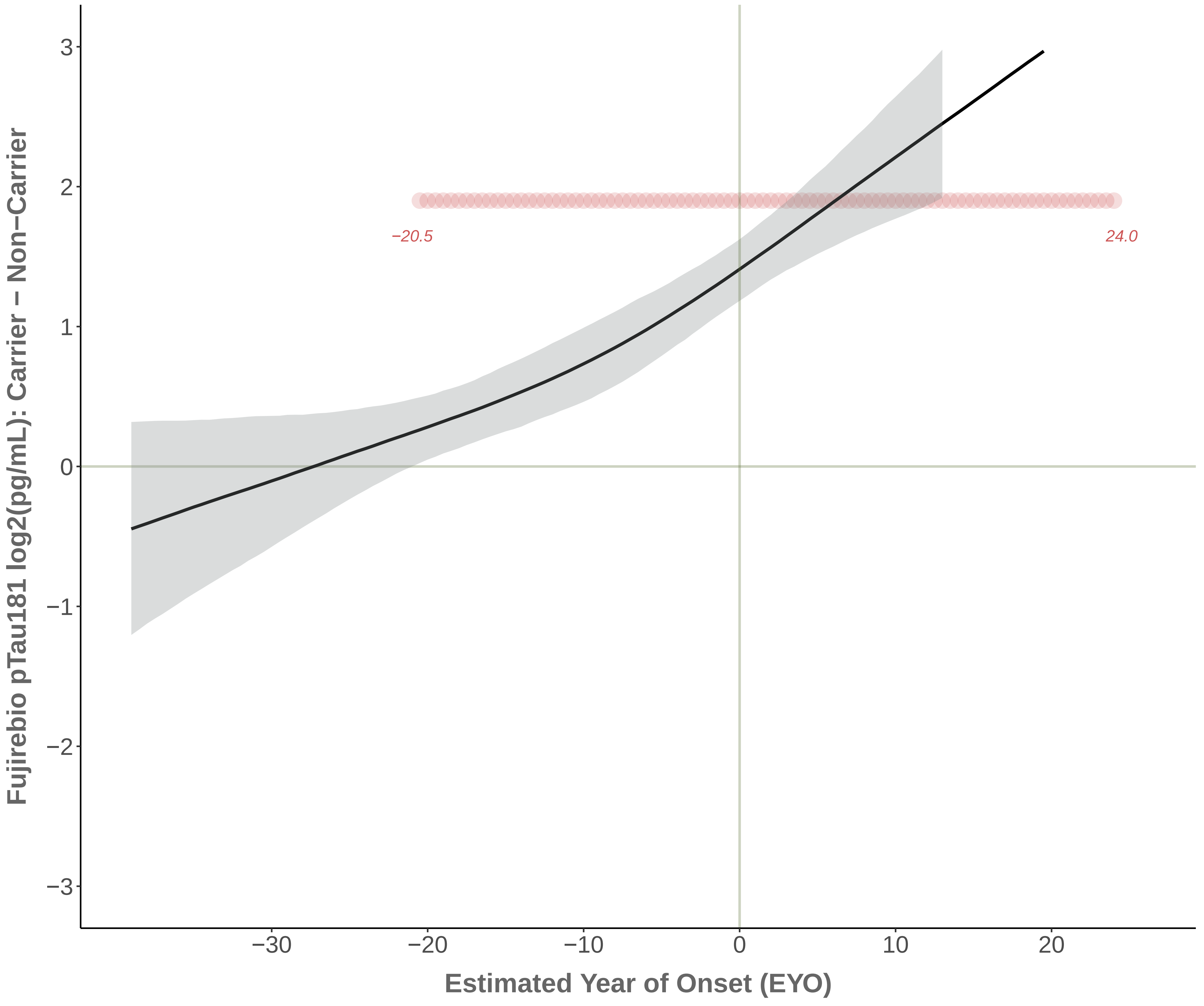

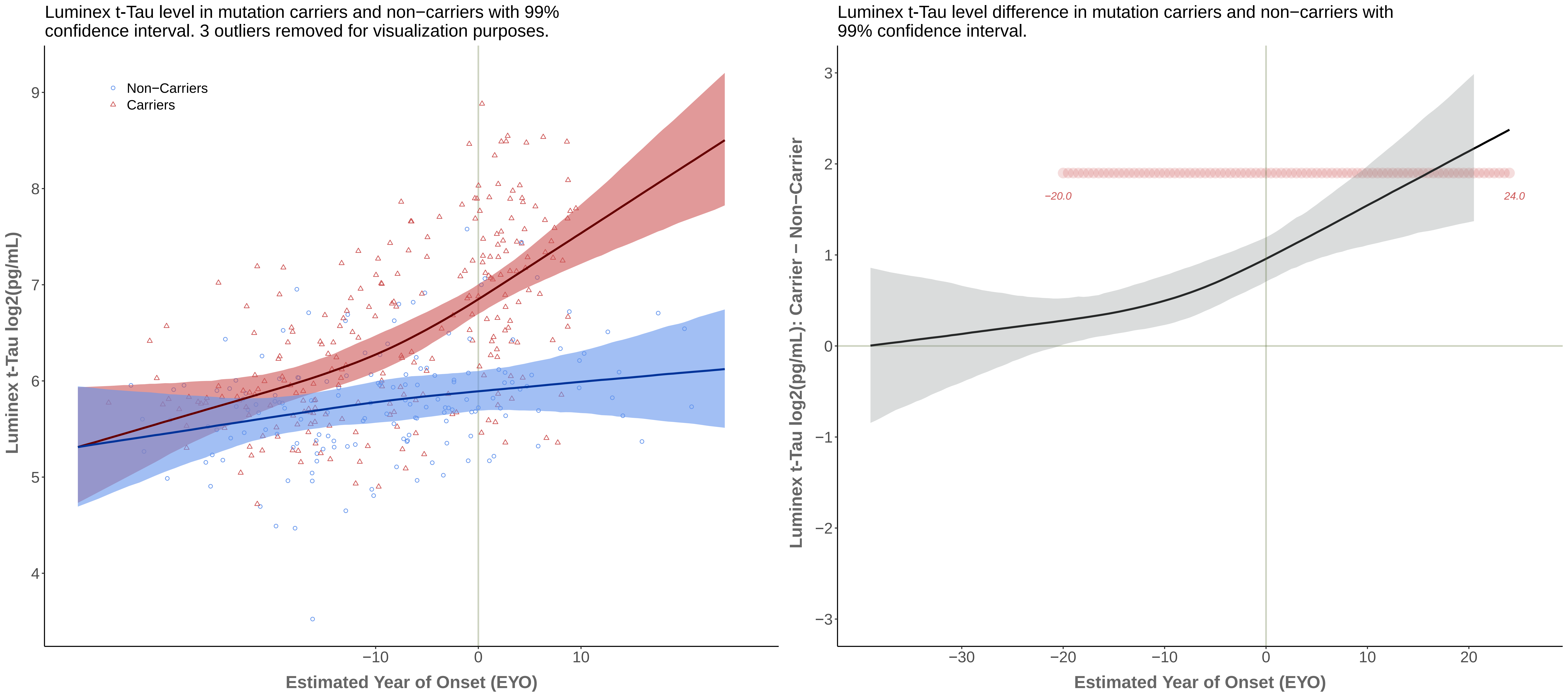

Luminex t-Tau level in mutation carriers and non−carriers with 99% confidence interval. 3 outliers removed for visualization purposes.
Non−Carriers
Carriers
Luminex t-Tau log2(pg/mL)
Estimated Year of Onset (EYO)
Luminex t-Tau level difference in mutation carriers and non−carriers with 99% confidence interval.
−20.0
24.0
Luminex t-Tau log2(pg/mL): Carrier − Non−Carrier
Estimated Year of Onset (EYO)

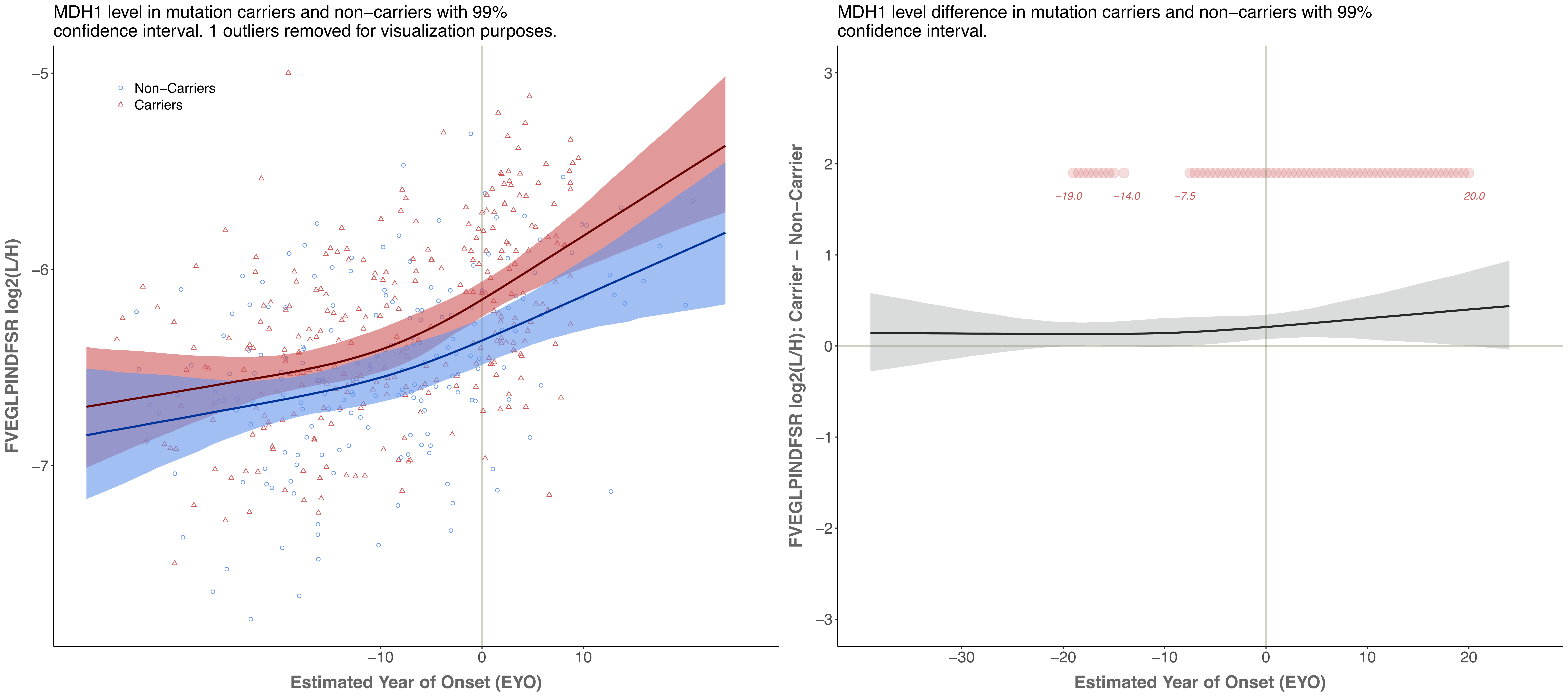

MDH1 level in mutation carriers and non−carriers with 99% confidence interval. 1 outliers removed for visualization purposes.
Non−Carriers
Carriers
FVEGLPINDFSR log2(L/H)
−5
−6
−7
−10
0
10
Estimated Year of Onset (EYO)
MDH1 level difference in mutation carriers and non−carriers with 99% confidence interval.
FVEGLPINDFSR log2(L/H): Carrier − Non−Carrier
3
2
1
0
−1
−2
−3
−19.0
−14.0
−7.5
20.0
−30
−20
−10
0
10
20
Estimated Year of Onset (EYO)

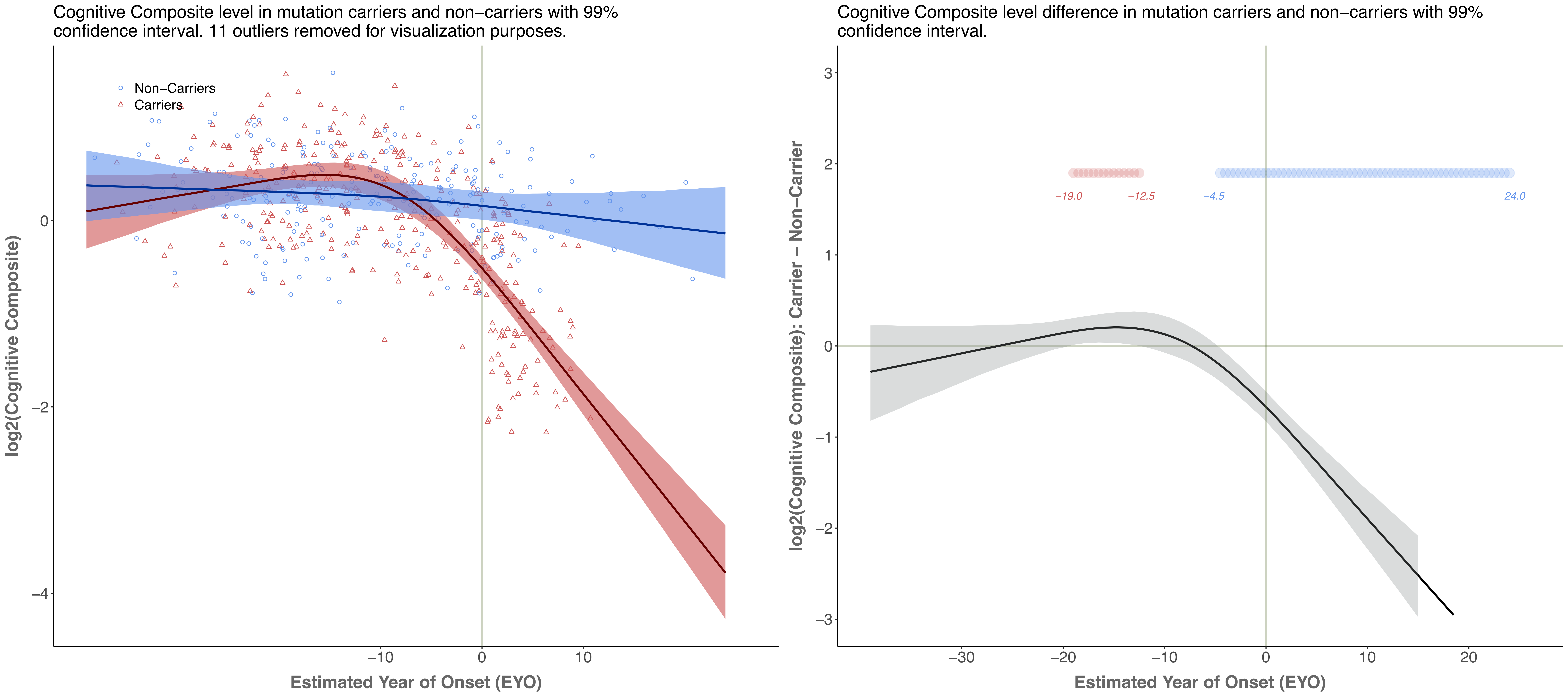
Cognitive Composite level in mutation carriers and non−carriers with 99% confidence interval. 11 outliers removed for visualization purposes.
Non−Carriers
Carriers
log2(Cognitive Composite)
Estimated Year of Onset (EYO)
0
−2
−4
−10
0
10
Cognitive Composite level difference in mutation carriers and non−carriers with 99% confidence interval.
log2(Cognitive Composite): Carrier − Non−Carrier
−19.0
−12.5
−4.5
24.0
3
2
1
0
−1
−2
−3
−30
−20
−10
0
10
20
Estimated Year of Onset (EYO)

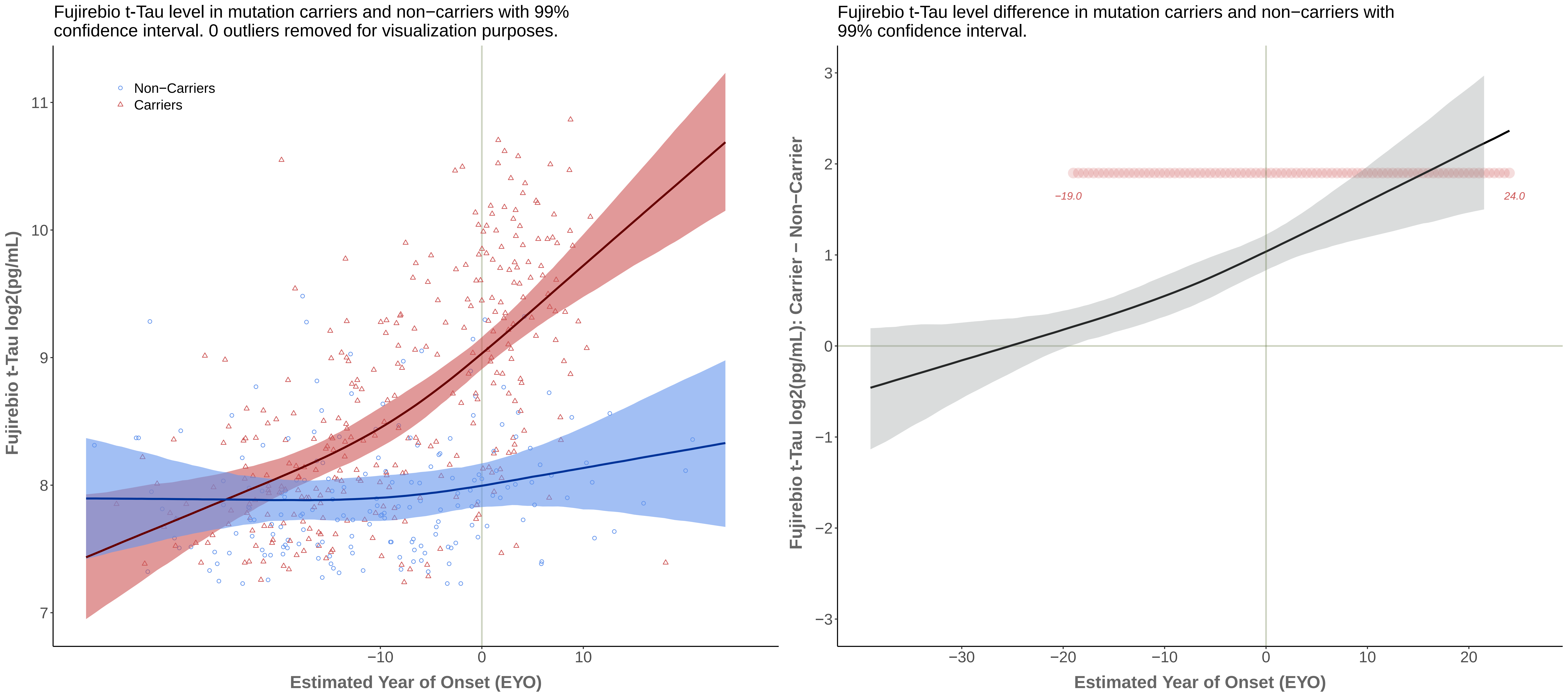
Fujirebio t-Tau level in mutation carriers and non−carriers with 99% confidence interval. 0 outliers removed for visualization purposes.
Non−Carriers
Carriers
Fujirebio t-Tau log2(pg/mL)
Estimated Year of Onset (EYO)
Fujirebio t-Tau level difference in mutation carriers and non−carriers with 99% confidence interval.
Fujirebio t-Tau log2(pg/mL): Carrier − Non−Carrier
−19.0
24.0
Estimated Year of Onset (EYO)

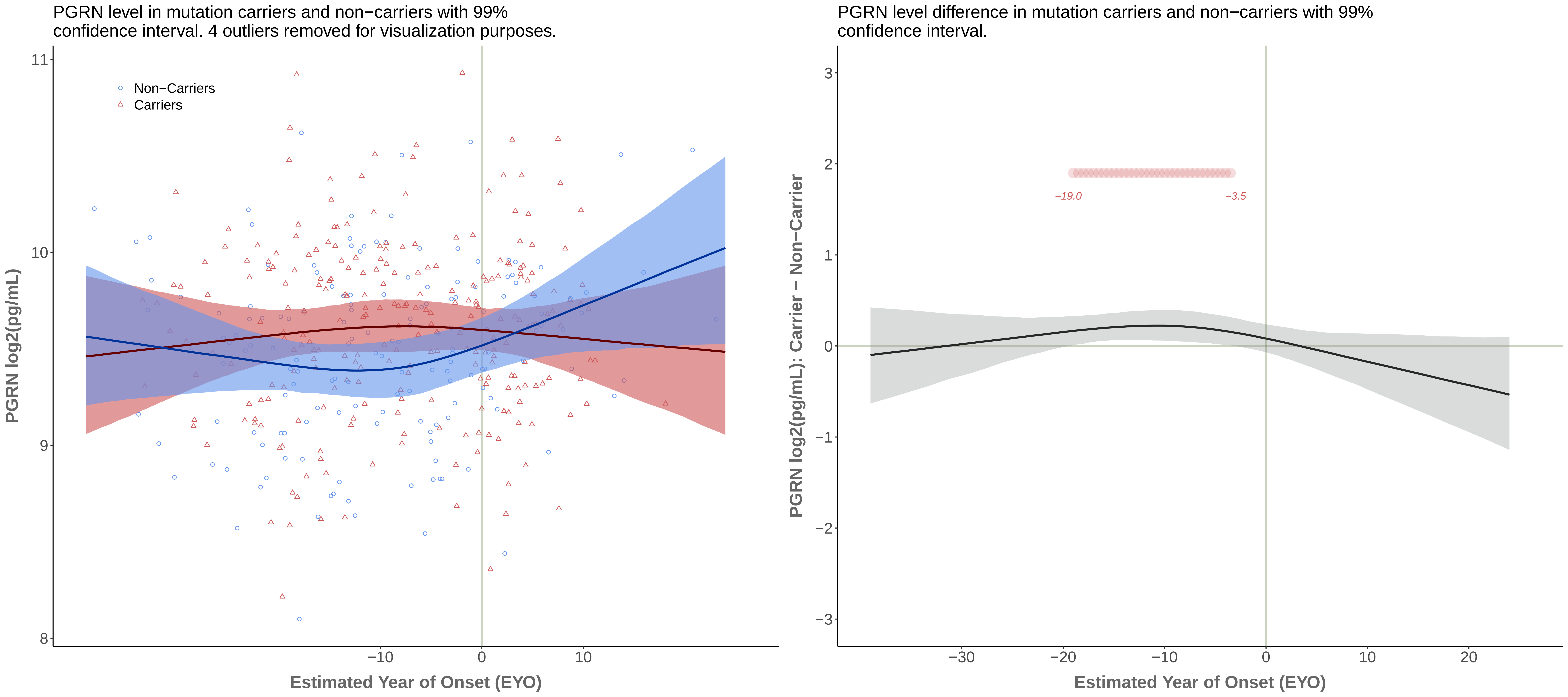
PGRN level in mutation carriers and non−carriers with 99% confidence interval. 4 outliers removed for visualization purposes.
Non−Carriers
Carriers
PGRN log2(pg/mL)
Estimated Year of Onset (EYO)
11
10
9
8
−10
0
10
PGRN level difference in mutation carriers and non−carriers with 99% confidence interval.
PGRN log2(pg/mL): Carrier − Non−Carrier
−19.0
−3.5
3
2
1
0
−1
−2
−3
−30
−20
−10
0
10
20
Estimated Year of Onset (EYO)

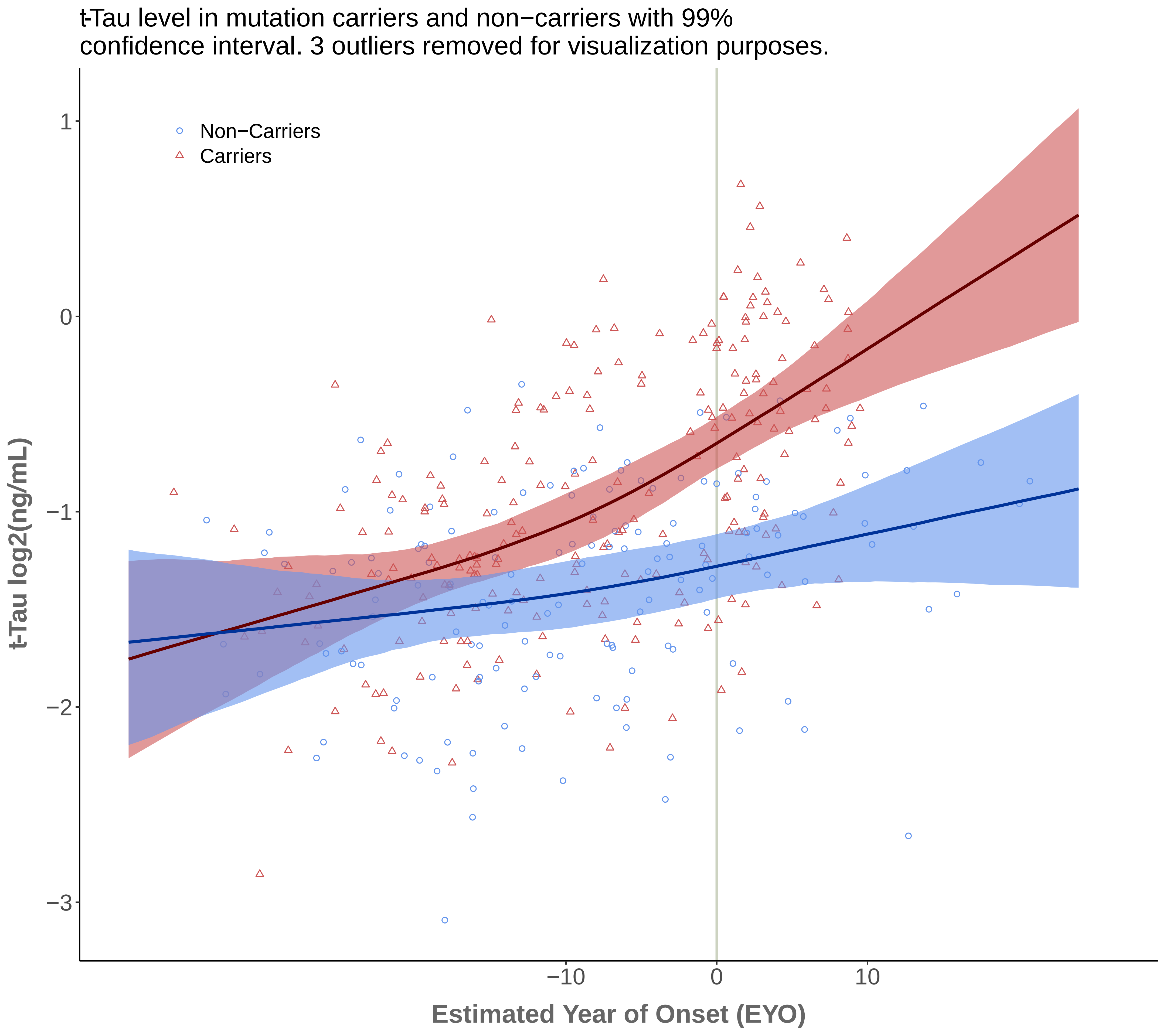

t-Tau level in mutation carriers and non−carriers with 99% confidence interval. 3 outliers removed for visualization purposes.
Non−Carriers
Carriers
t-Tau log2(ng/mL)
Estimated Year of Onset (EYO)
1
0
−1
−2
−3
−10
0
10

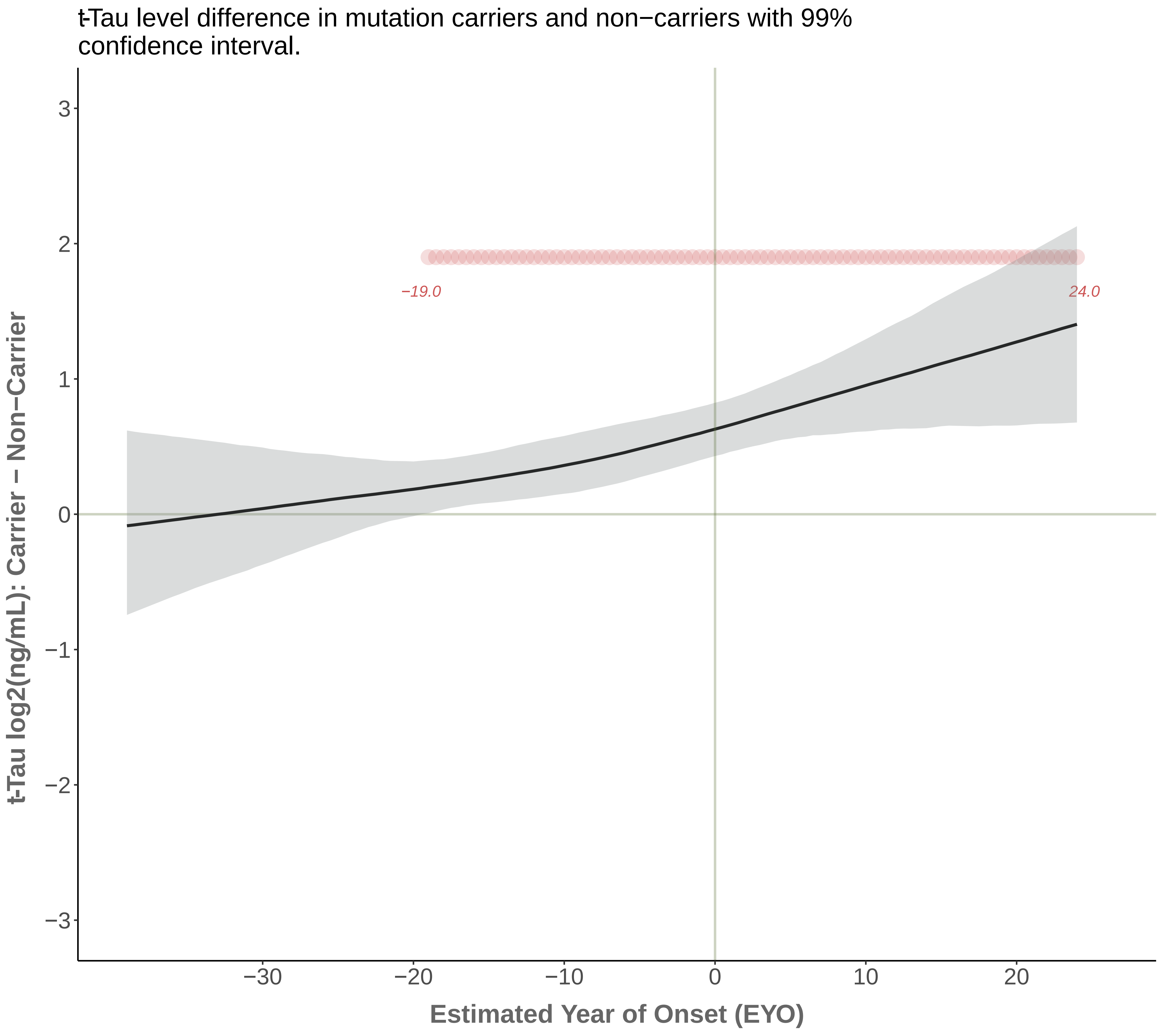

t-Tau level difference in mutation carriers and non−carriers with 99% confidence interval.
t-Tau log2(ng/mL): Carrier − Non−Carrier
Estimated Year of Onset (EYO)
−19.0
24.0
3
2
1
0
−1
−2
−3
−30
−20
−10
0
10
20

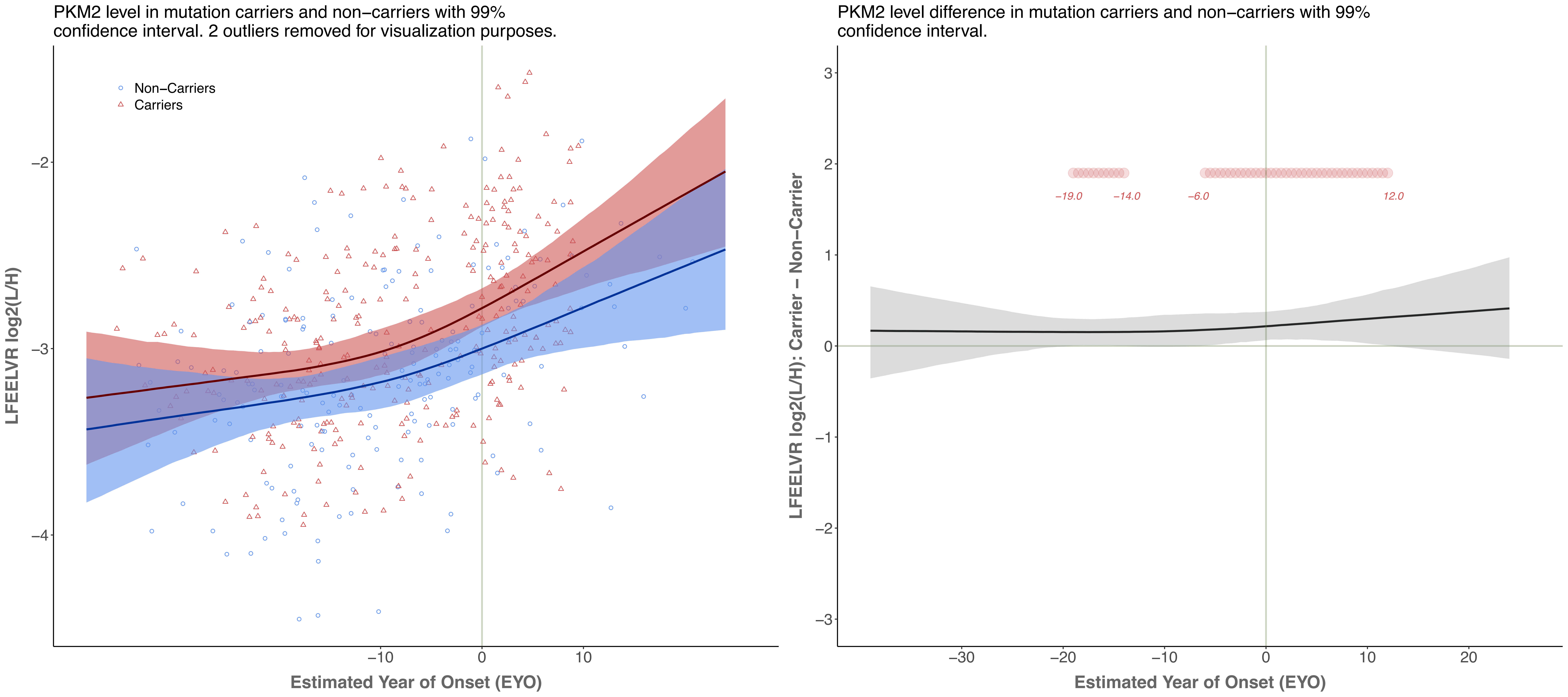

PKM2 level in mutation carriers and non−carriers with 99% confidence interval. 2 outliers removed for visualization purposes.
Non−Carriers
Carriers
LFEELVR log2(L/H)
−2
−3
−4
−10
0
10
Estimated Year of Onset (EYO)
PKM2 level difference in mutation carriers and non−carriers with 99% confidence interval.
LFEELVR log2(L/H): Carrier − Non−Carrier
3
2
1
0
−1
−2
−3
−19.0
−14.0
−6.0
12.0
−30
−20
−10
0
10
20
Estimated Year of Onset (EYO)

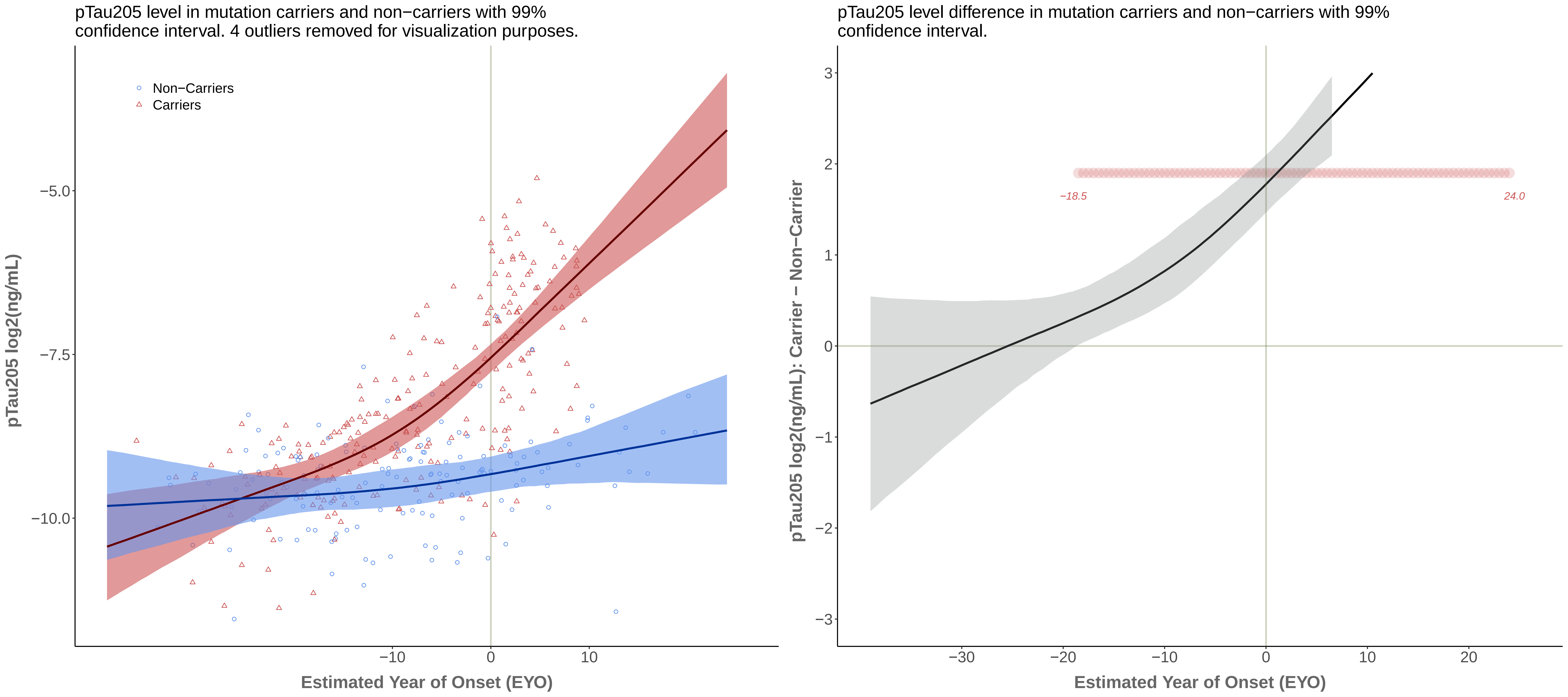
pTau205 level in mutation carriers and non−carriers with 99% confidence interval. 4 outliers removed for visualization purposes.
Non−Carriers
Carriers
−5.0
−7.5
−10.0
−10
0
10
pTau205 log2(ng/mL)
Estimated Year of Onset (EYO)
pTau205 level difference in mutation carriers and non−carriers with 99% confidence interval.
3
2
1
0
−1
−2
−3
−18.5
24.0
−30
−20
−10
0
10
20
pTau205 log2(ng/mL): Carrier − Non−Carrier
Estimated Year of Onset (EYO)

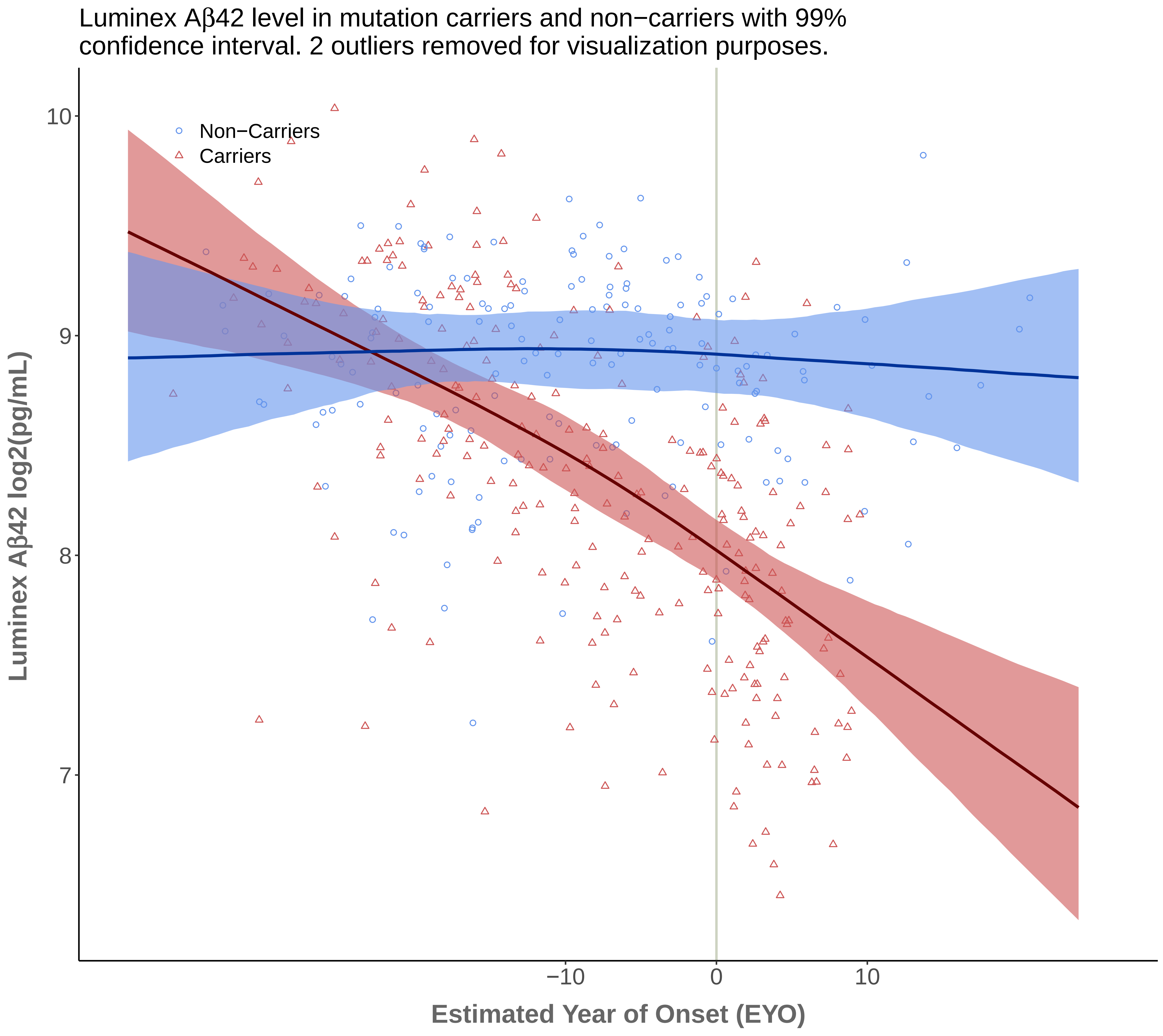
Luminex Aβ42 level in mutation carriers and non−carriers with 99% confidence interval. 2 outliers removed for visualization purposes.
Non−Carriers
Carriers
Luminex Aβ42 log2(pg/mL)
Estimated Year of Onset (EYO)
10
9
8
7
−10
0
10

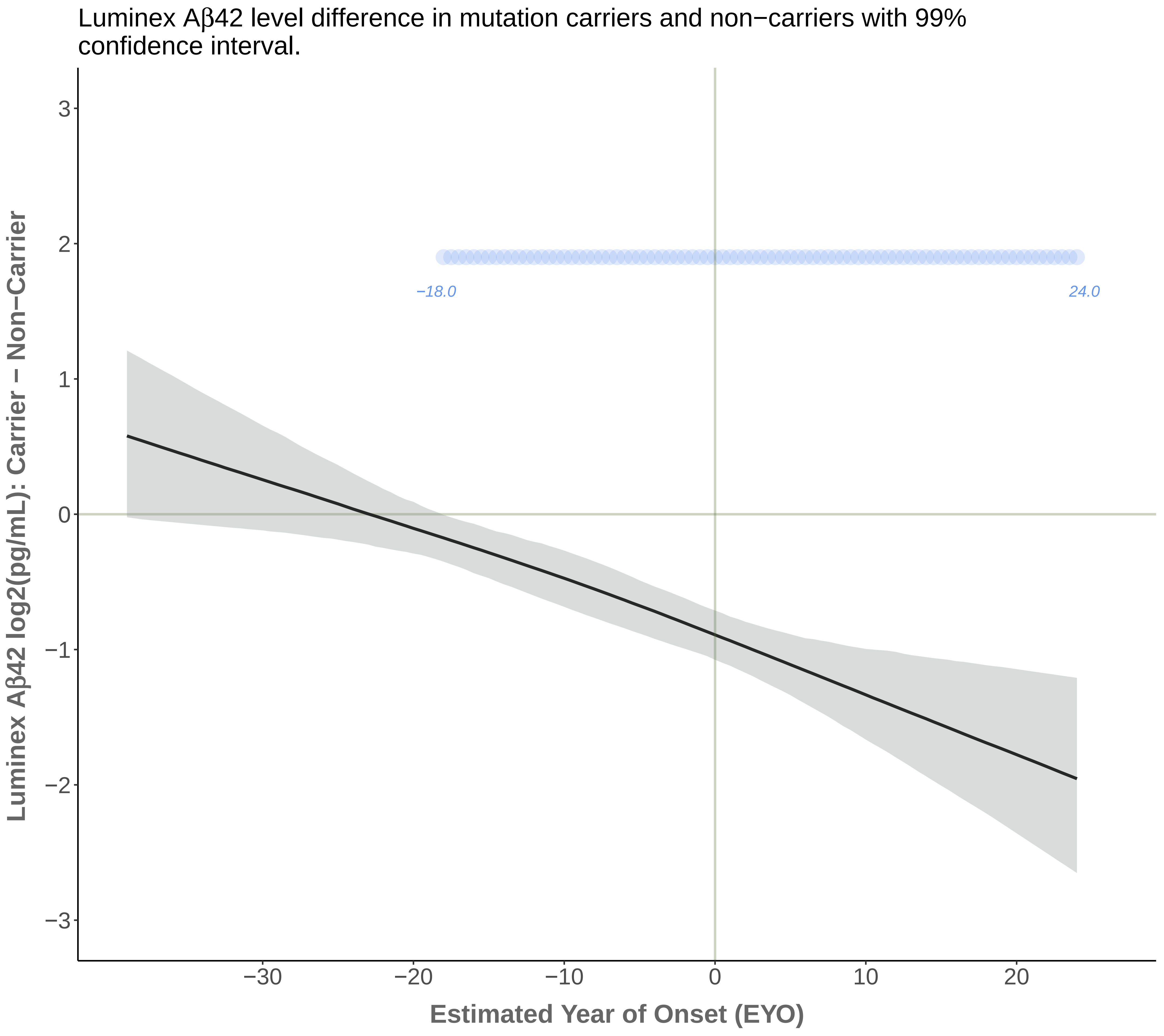
Luminex Aβ42 level difference in mutation carriers and non−carriers with 99% confidence interval.
−18.0
24.0
Luminex Aβ42 log2(pg/mL): Carrier − Non−Carrier
Estimated Year of Onset (EYO)
3
2
1
0
−1
−2
−3
−30
−20
−10
0
10
20

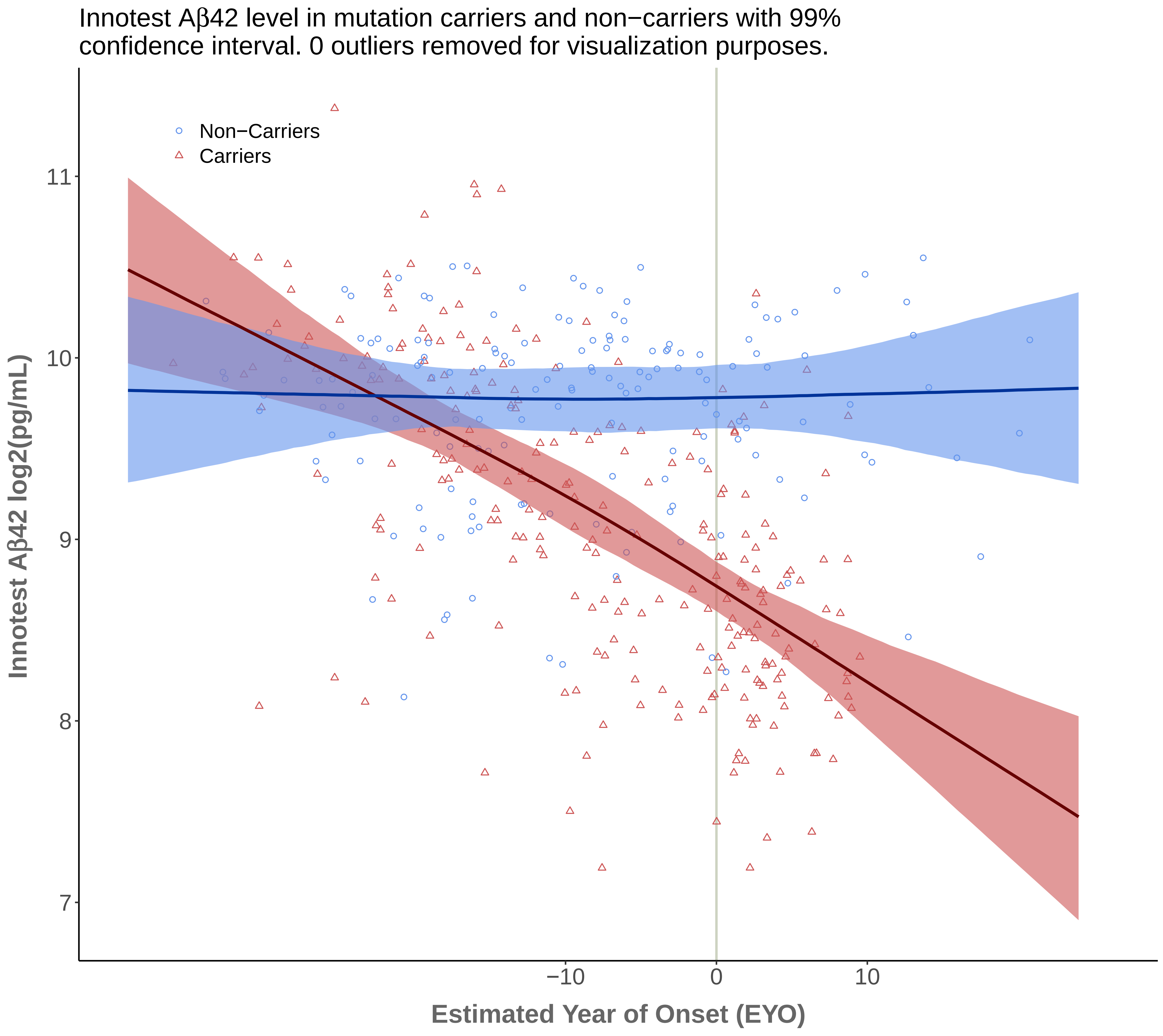

Innotest Aβ42 level in mutation carriers and non−carriers with 99% confidence interval. 0 outliers removed for visualization purposes.
Non−Carriers
Carriers
Innotest Aβ42 log2(pg/mL)
11
10
9
8
7
−10
0
10
Estimated Year of Onset (EYO)

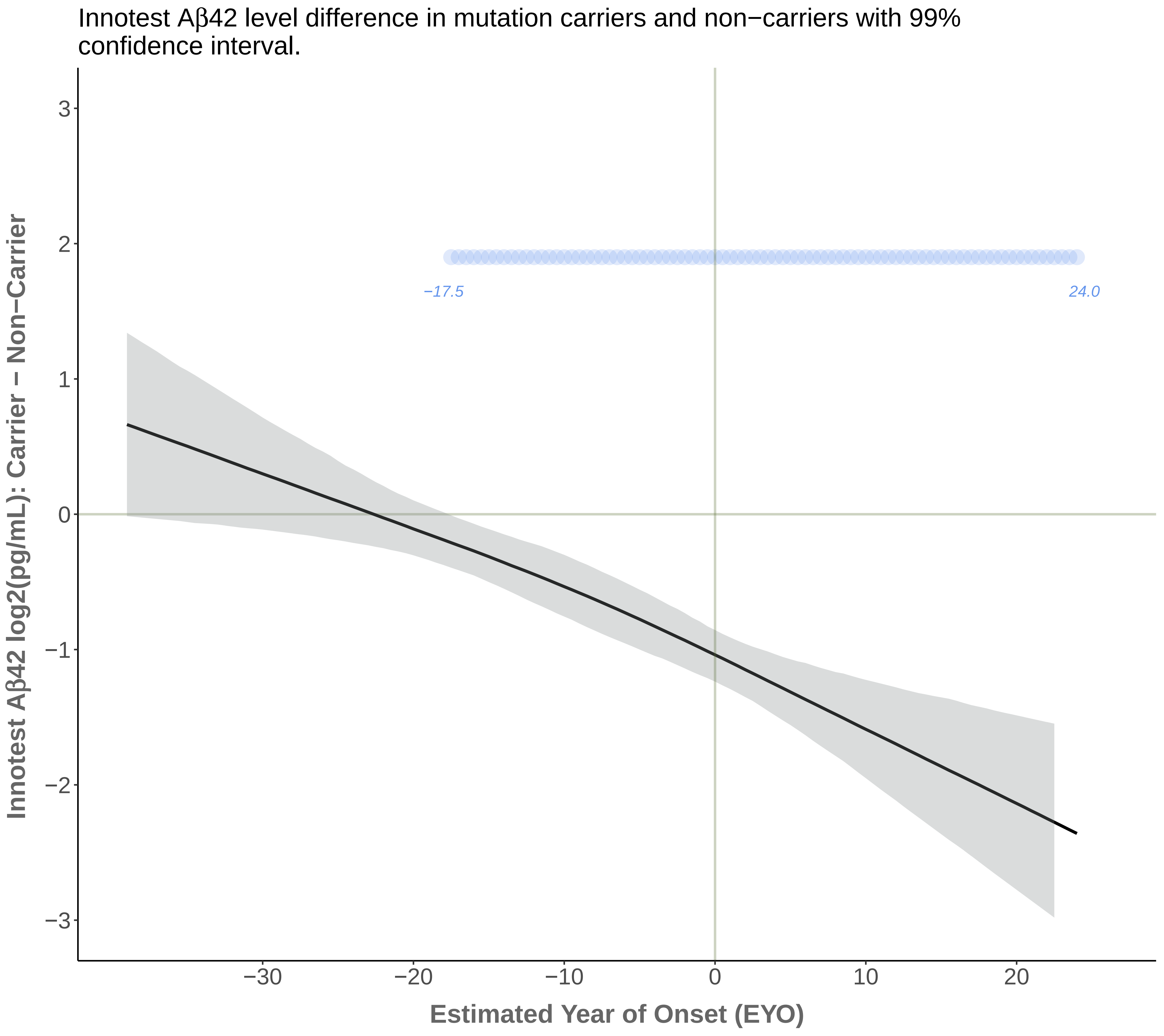

Innotest Aβ42 level difference in mutation carriers and non−carriers with 99% confidence interval.
−17.5
24.0
Innotest Aβ42 log2(pg/mL): Carrier − Non−Carrier
3
2
1
0
−1
−2
−3
−30
−20
−10
0
10
20
Estimated Year of Onset (EYO)

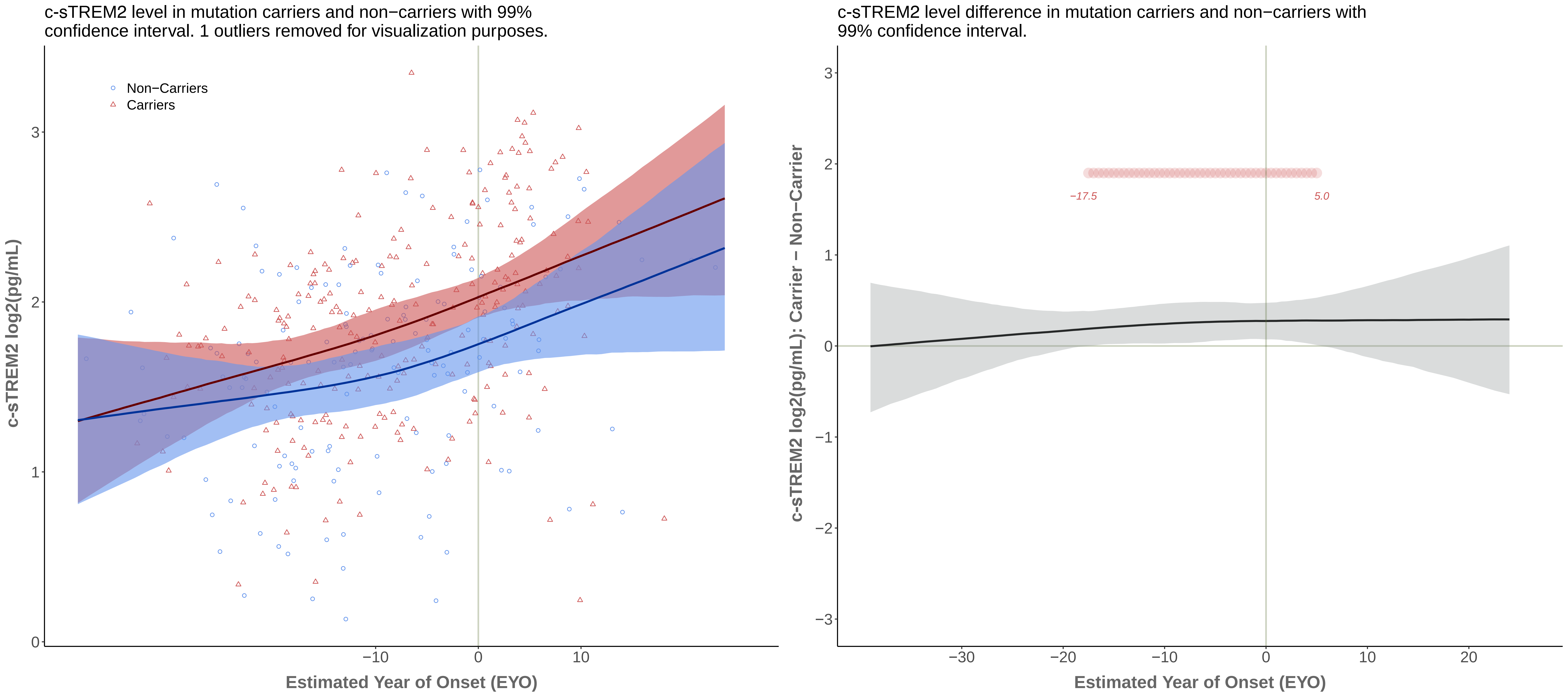
c-sTREM2 level in mutation carriers and non−carriers with 99% confidence interval. 1 outliers removed for visualization purposes.
Non−Carriers
Carriers
c-sTREM2 log2(pg/mL)
Estimated Year of Onset (EYO)
c-sTREM2 level difference in mutation carriers and non−carriers with 99% confidence interval.
−17.5
5.0
c-sTREM2 log2(pg/mL): Carrier − Non−Carrier
Estimated Year of Onset (EYO)

pTau202/Tau202 ratio in mutation carriers and non−carriers with 99% confidence interval. 1 outliers removed for visualization purposes.

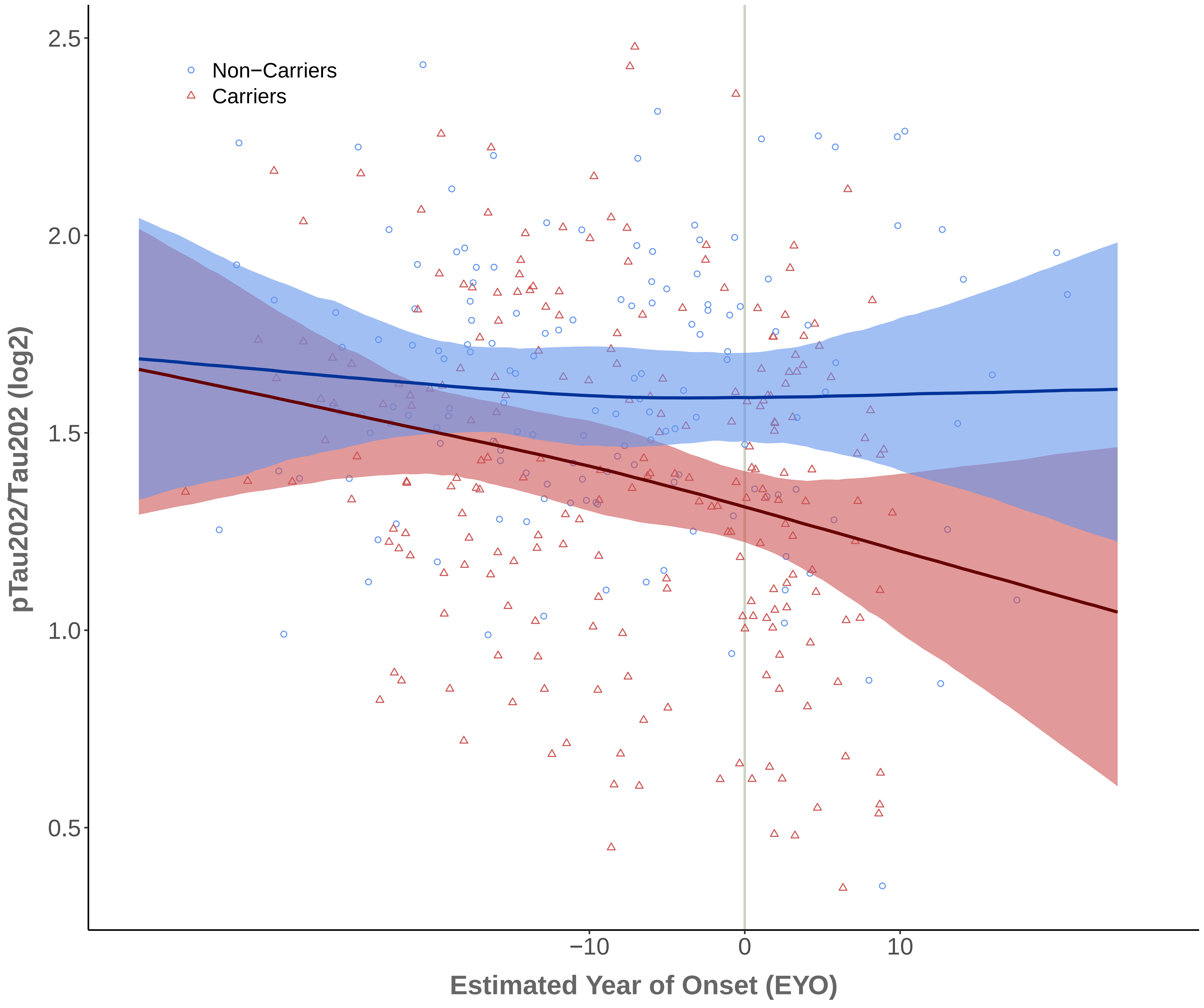

pTau202/Tau202 ratio difference in mutation carriers and non−carriers with 99% confidence interval.

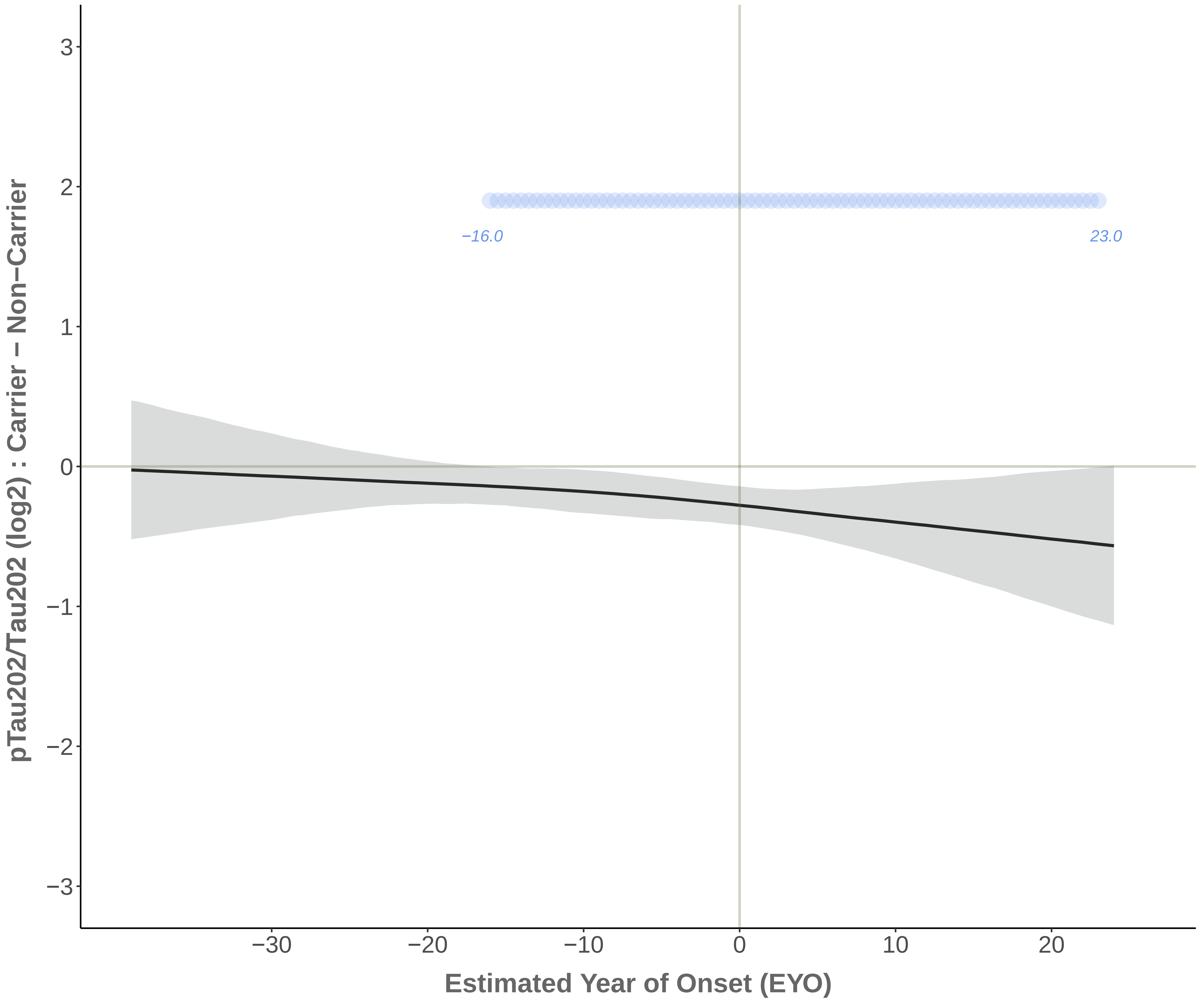

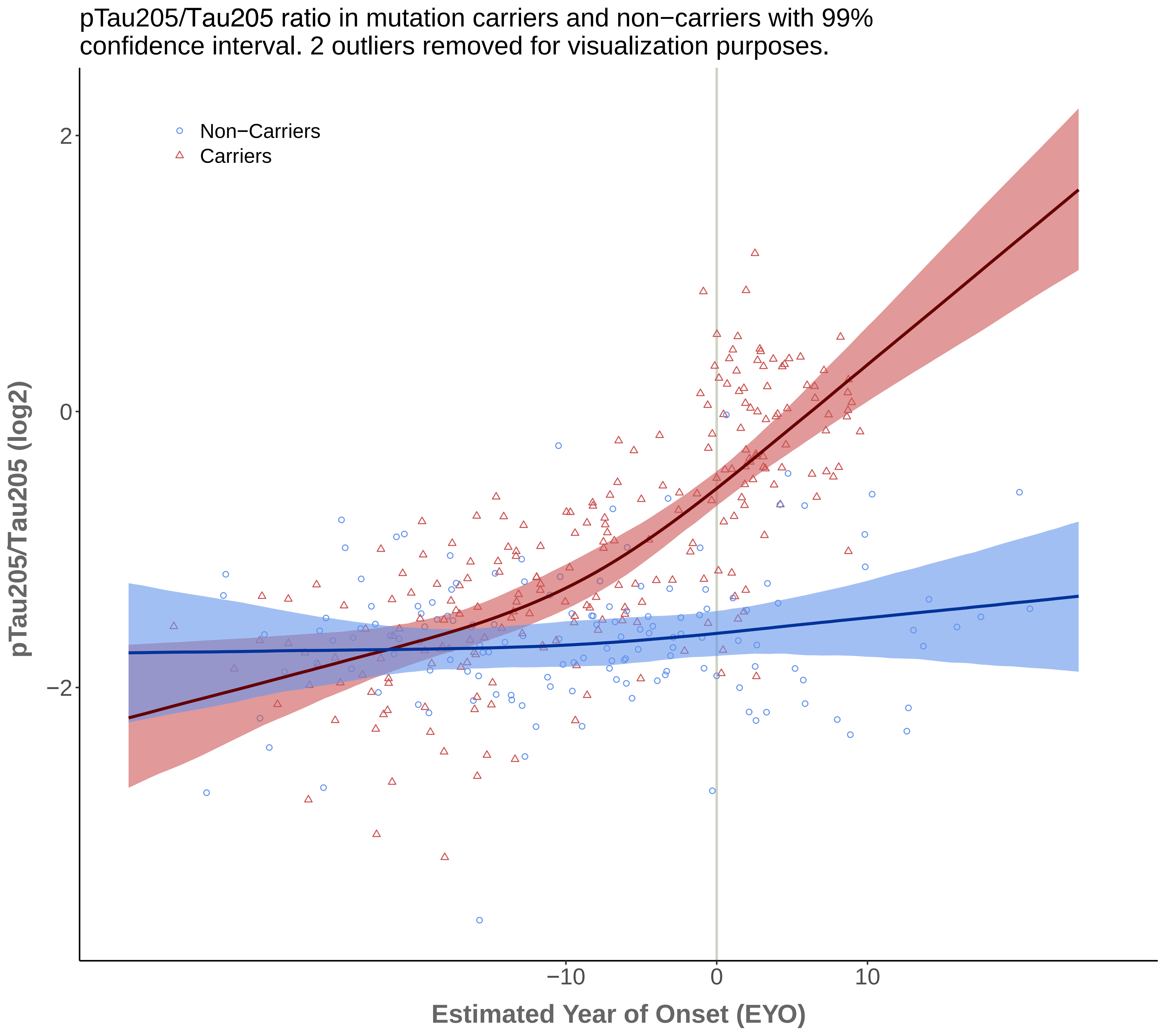
pTau205/Tau205 ratio in mutation carriers and non−carriers with 99% confidence interval. 2 outliers removed for visualization purposes.
Non−Carriers
Carriers
2
0
−2
−10
0
10
pTau205/Tau205 (log2)
Estimated Year of Onset (EYO)

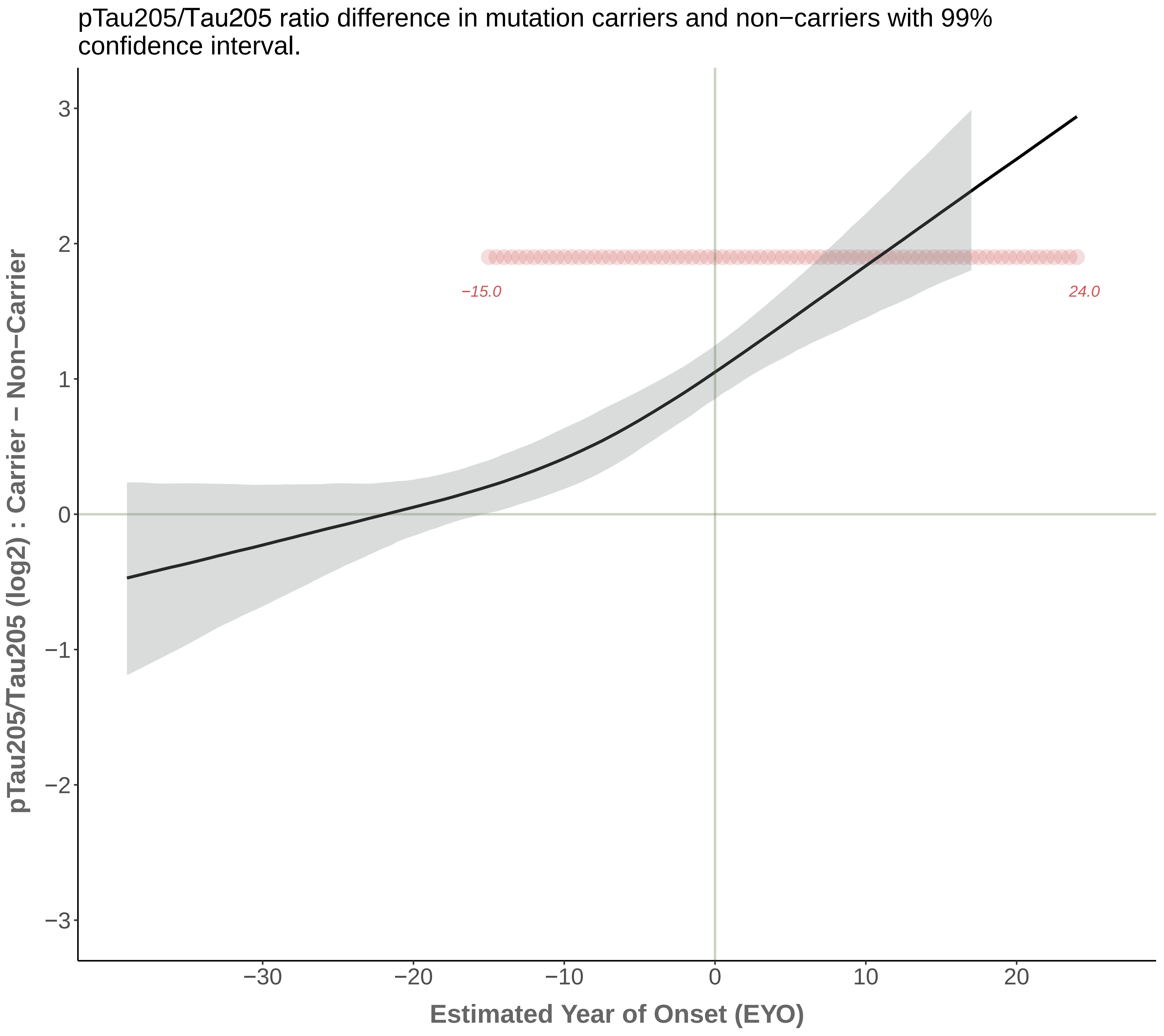
pTau205/Tau205 ratio difference in mutation carriers and non−carriers with 99% confidence interval.
3
2
1
0
−1
−2
−3
−15.0
24.0
−30
−20
−10
0
10
20
pTau205/Tau205 (log2) : Carrier − Non−Carrier
Estimated Year of Onset (EYO)

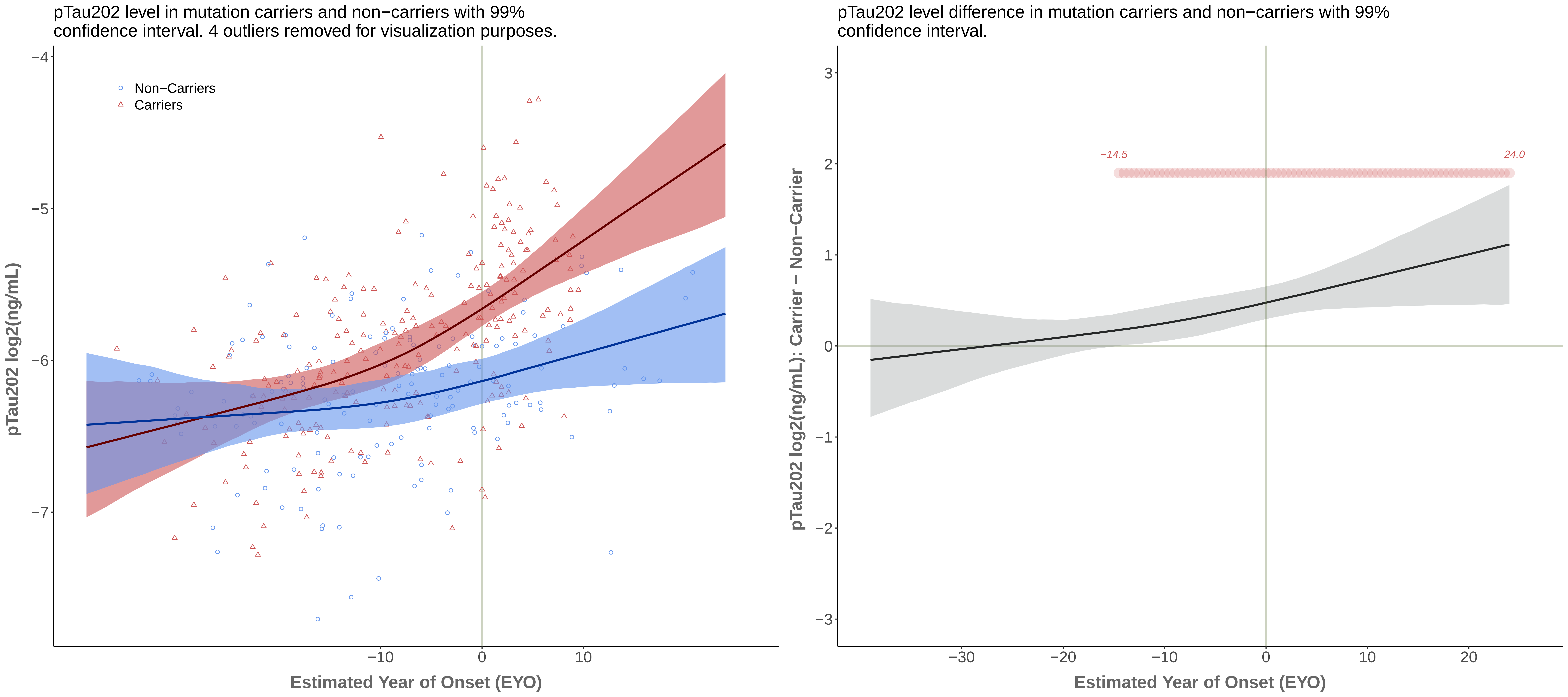
pTau202 level in mutation carriers and non−carriers with 99% confidence interval. 4 outliers removed for visualization purposes.
Non−Carriers
Carriers
pTau202 log2(ng/mL)
−4
−5
−6
−7
−10
0
10
Estimated Year of Onset (EYO)
pTau202 level difference in mutation carriers and non−carriers with 99% confidence interval.
pTau202 log2(ng/mL): Carrier − Non−Carrier
3
2
1
0
−1
−2
−3
−14.5
24.0
−30
−20
−10
0
10
20
Estimated Year of Onset (EYO)

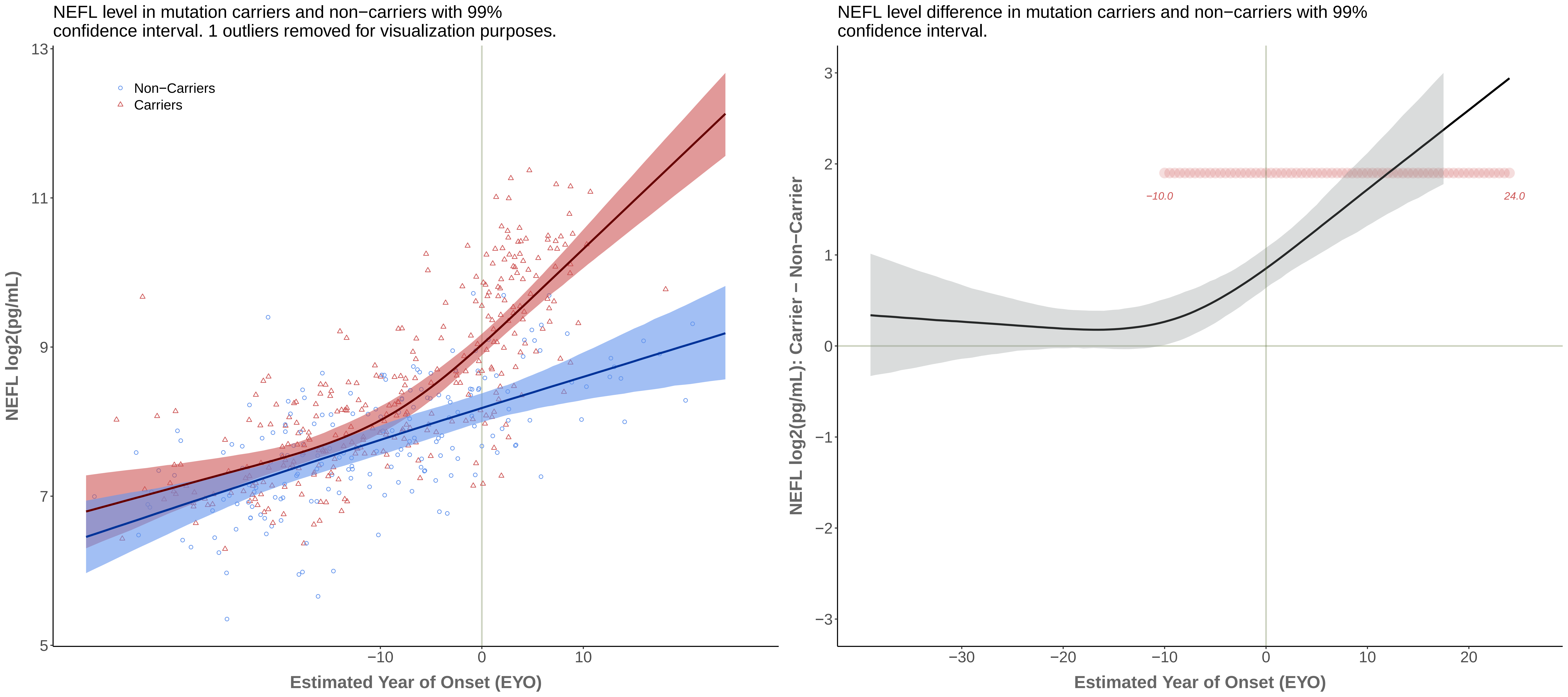
NEFL level in mutation carriers and non−carriers with 99% confidence interval. 1 outliers removed for visualization purposes.
Non−Carriers
Carriers
NEFL log2(pg/mL)
Estimated Year of Onset (EYO)
13
11
9
7
5
−10
0
10
NEFL level difference in mutation carriers and non−carriers with 99% confidence interval.
NEFL log2(pg/mL): Carrier − Non−Carrier
−10.0
24.0
3
2
1
0
−1
−2
−3
−30
−20
−10
0
10
20
Estimated Year of Onset (EYO)

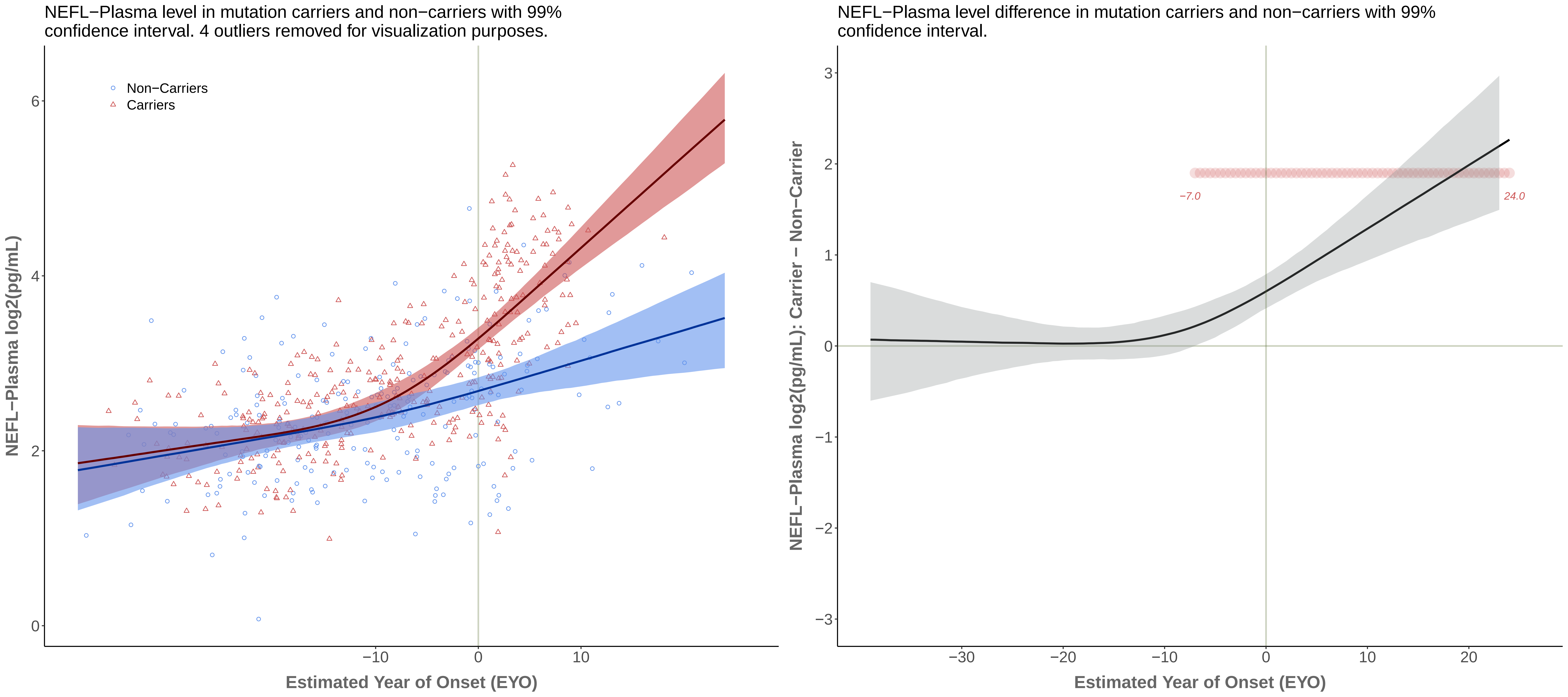

NEFL−Plasma level in mutation carriers and non−carriers with 99% confidence interval. 4 outliers removed for visualization purposes.
Non−Carriers
Carriers
NEFL−Plasma log2(pg/mL)
0
2
4
6
−10
0
10
Estimated Year of Onset (EYO)
NEFL−Plasma level difference in mutation carriers and non−carriers with 99% confidence interval.
NEFL−Plasma log2(pg/mL): Carrier − Non−Carrier
−3
−2
−1
0
1
2
3
−7.0
24.0
−30
−20
−10
0
10
20
Estimated Year of Onset (EYO)

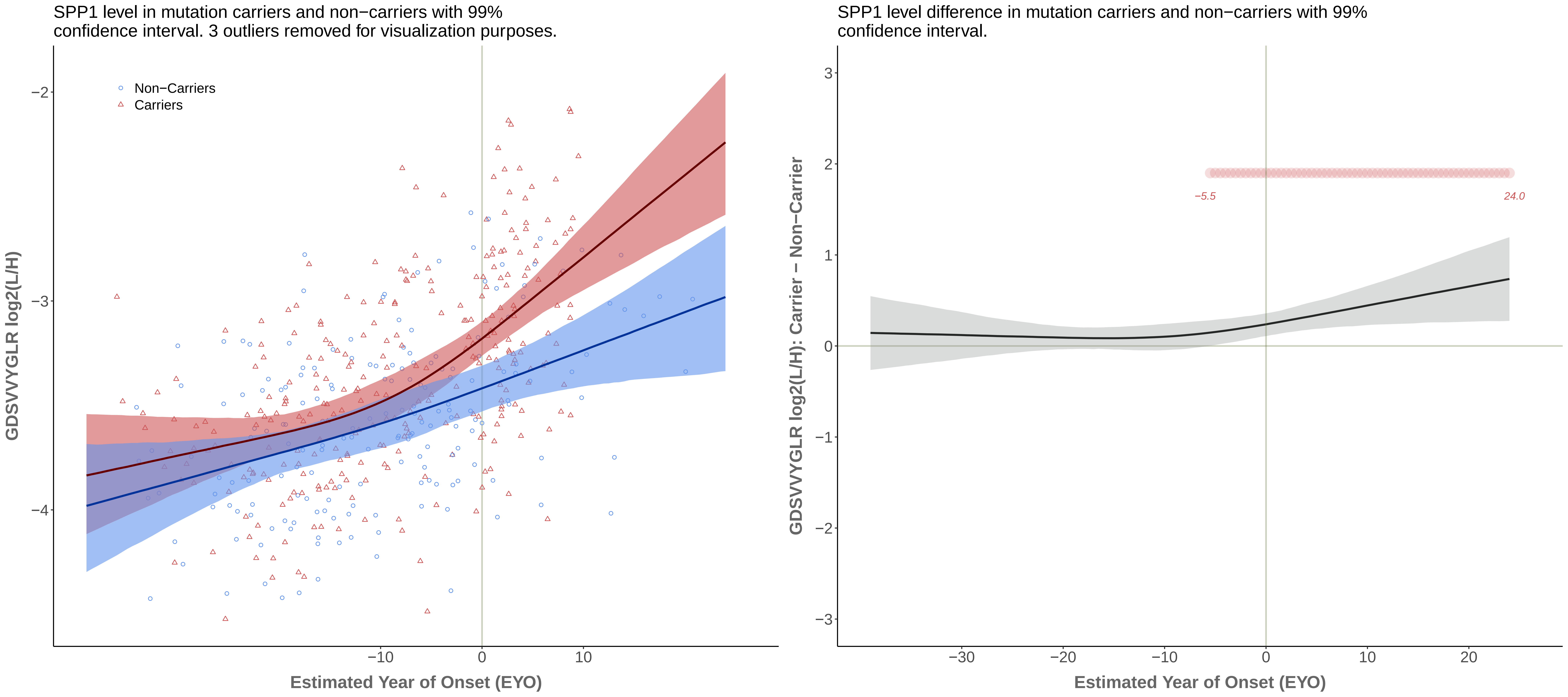

SPP1 level in mutation carriers and non−carriers with 99% confidence interval. 3 outliers removed for visualization purposes.
Non−Carriers
Carriers
GDSVVYGLR log2(L/H)
Estimated Year of Onset (EYO)
SPP1 level difference in mutation carriers and non−carriers with 99% confidence interval.
−5.5
24.0
GDSVVYGLR log2(L/H): Carrier − Non−Carrier
Estimated Year of Onset (EYO)

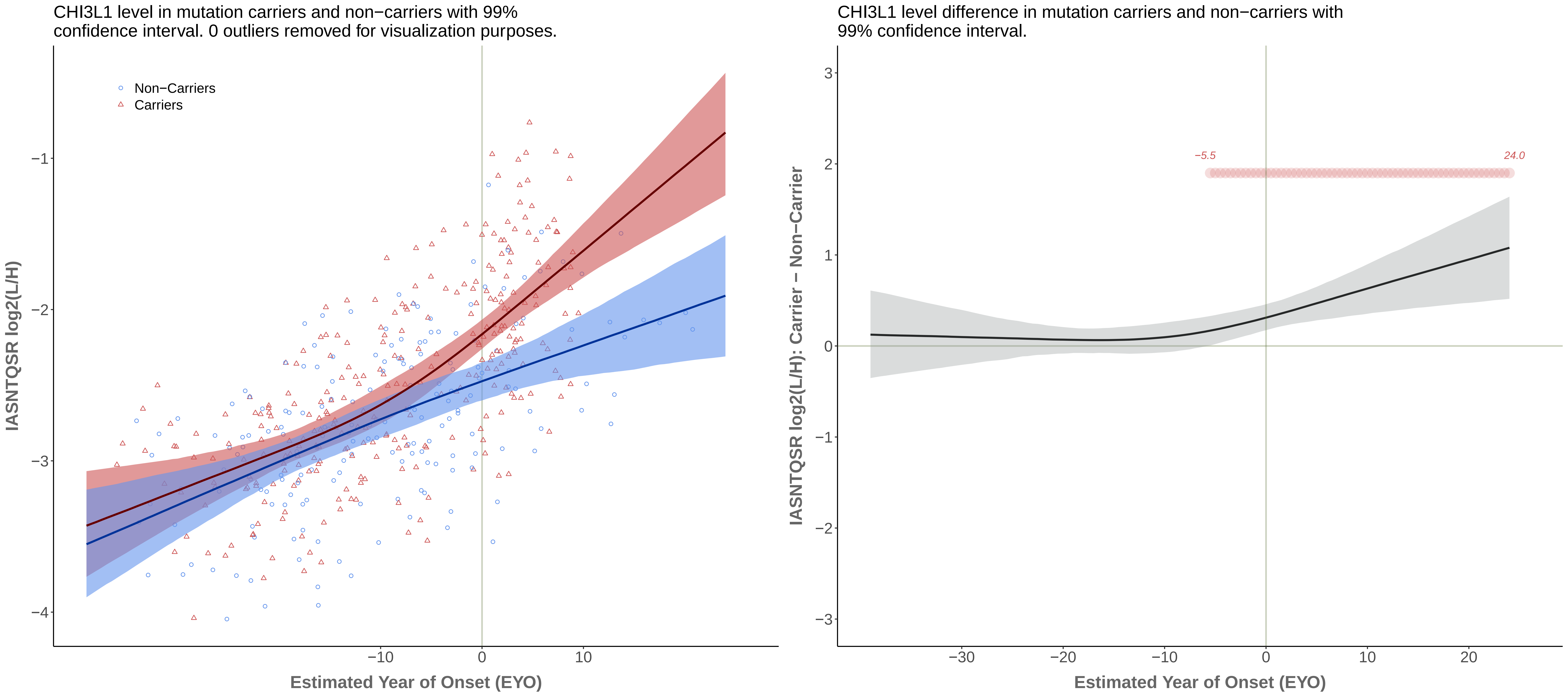
CHI3L1 level in mutation carriers and non−carriers with 99% confidence interval. 0 outliers removed for visualization purposes.
Non−Carriers
Carriers
IASNTQSR log2(L/H)
−1
−2
−3
−4
−10
0
10
Estimated Year of Onset (EYO)
CHI3L1 level difference in mutation carriers and non−carriers with 99% confidence interval.
IASNTQSR log2(L/H): Carrier − Non−Carrier
3
2
1
0
−1
−2
−3
−5.5
24.0
−30
−20
−10
0
10
20
Estimated Year of Onset (EYO)

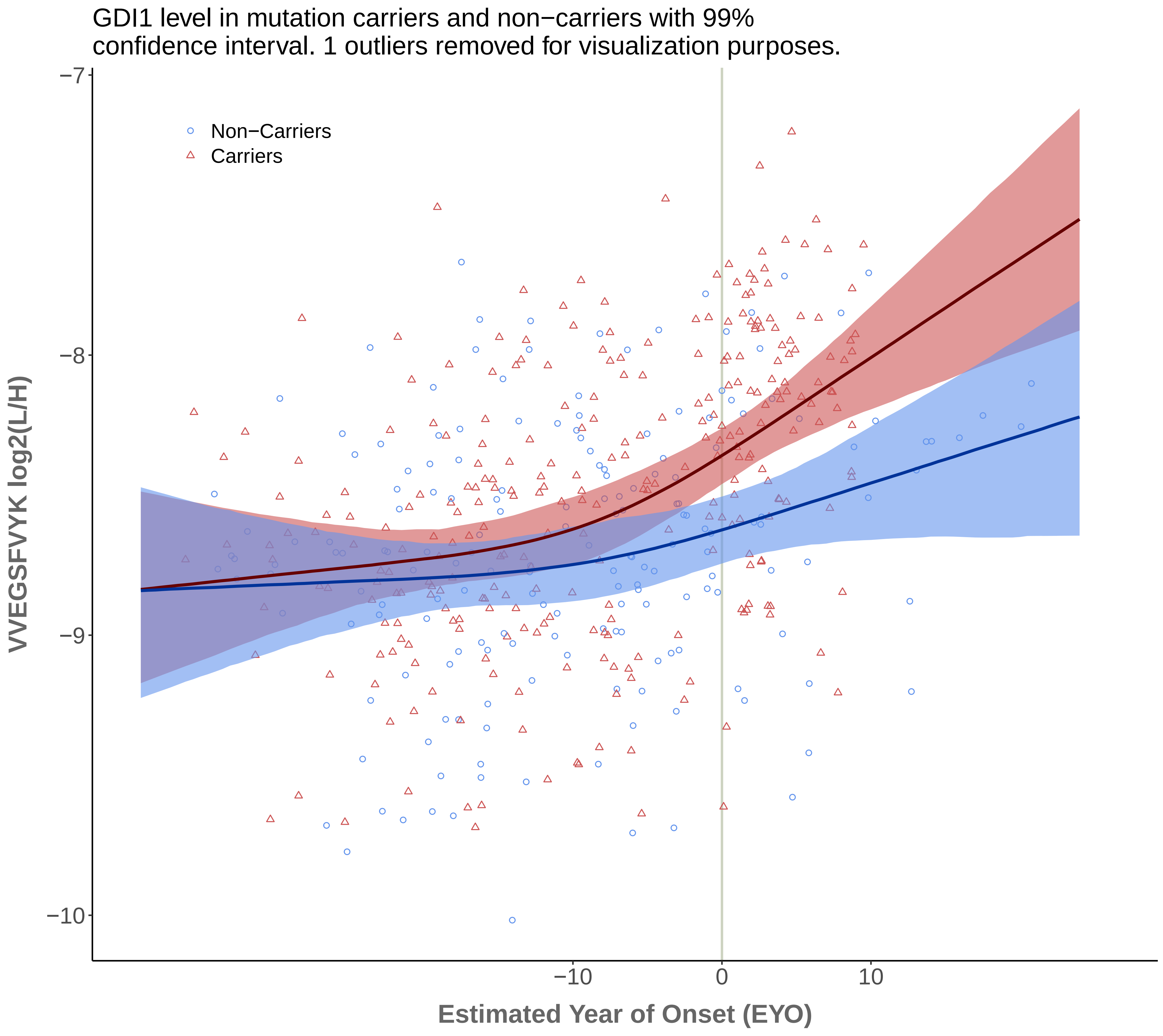
GDI1 level in mutation carriers and non−carriers with 99%
confidence interval. 1 outliers removed for visualization purposes.
Non−Carriers
Carriers
VVEGSFVYK log2(L/H)
−7
−8
−9
−10
−10
0
10
Estimated Year of Onset (EYO)

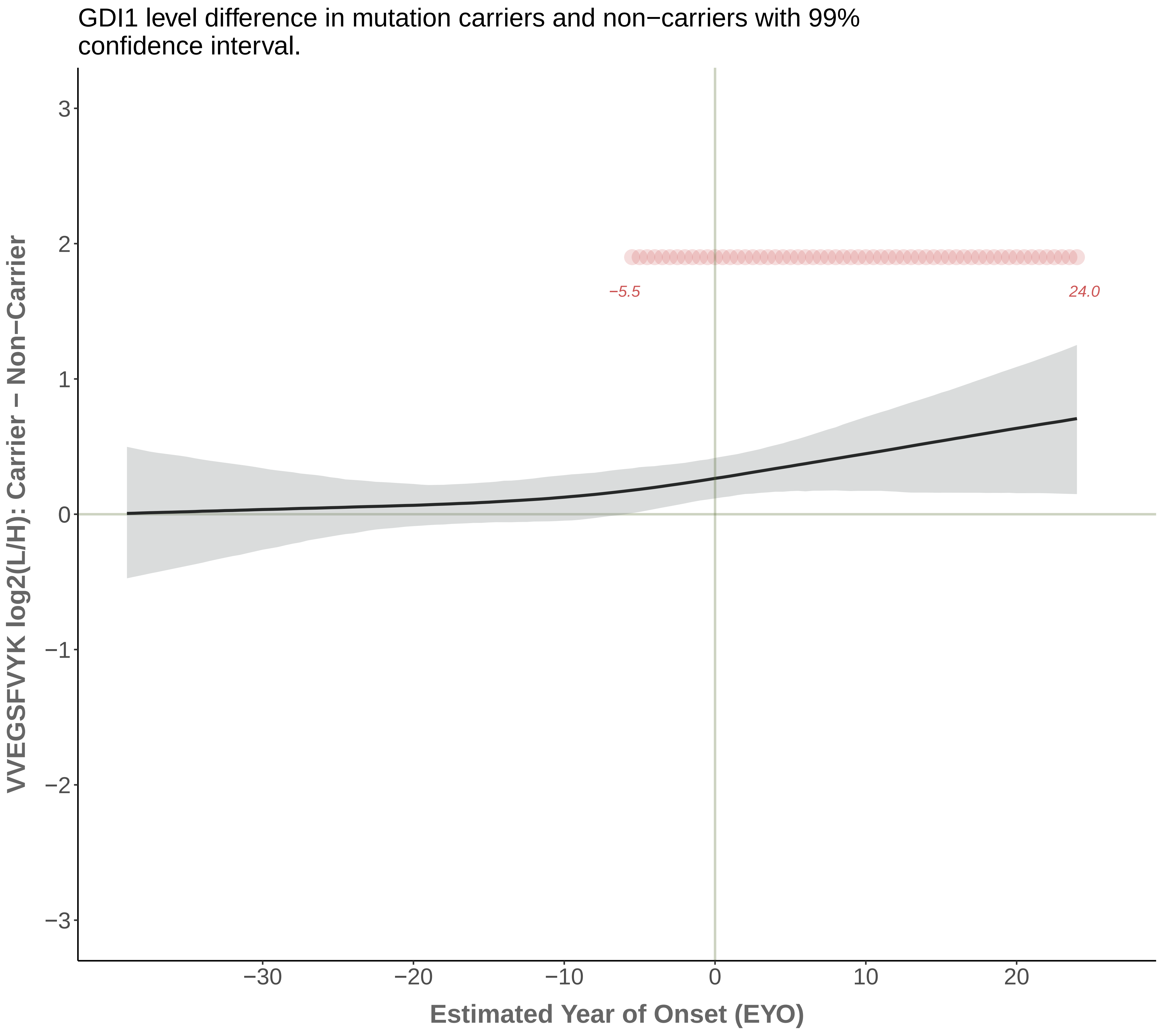
GDI1 level difference in mutation carriers and non−carriers with 99%
confidence interval.
VVEGSFVYK log2(L/H): Carrier − Non−Carrier
3
2
1
0
−1
−2
−3
−5.5
24.0
−30
−20
−10
0
10
20
Estimated Year of Onset (EYO)

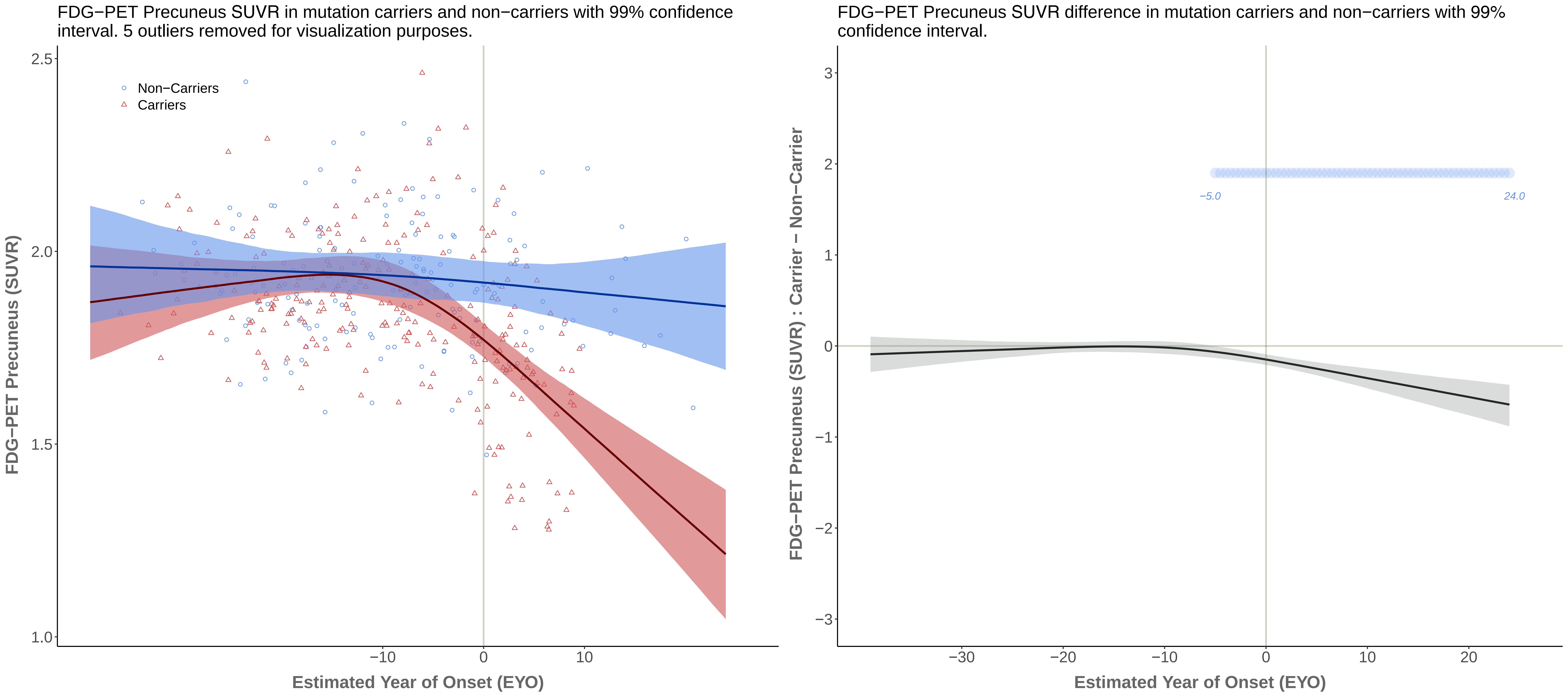
FDG−PET Precuneus SUVR in mutation carriers and non−carriers with 99% confidence interval. 5 outliers removed for visualization purposes.
Non−Carriers
Carriers
FDG−PET Precuneus (SUVR)
2.5
2.0
1.5
1.0
−10
0
10
Estimated Year of Onset (EYO)
FDG−PET Precuneus SUVR difference in mutation carriers and non−carriers with 99% confidence interval.
FDG−PET Precuneus (SUVR) : Carrier − Non−Carrier
3
2
1
0
−1
−2
−3
−5.0
24.0
−30
−20
−10
0
10
20
Estimated Year of Onset (EYO)

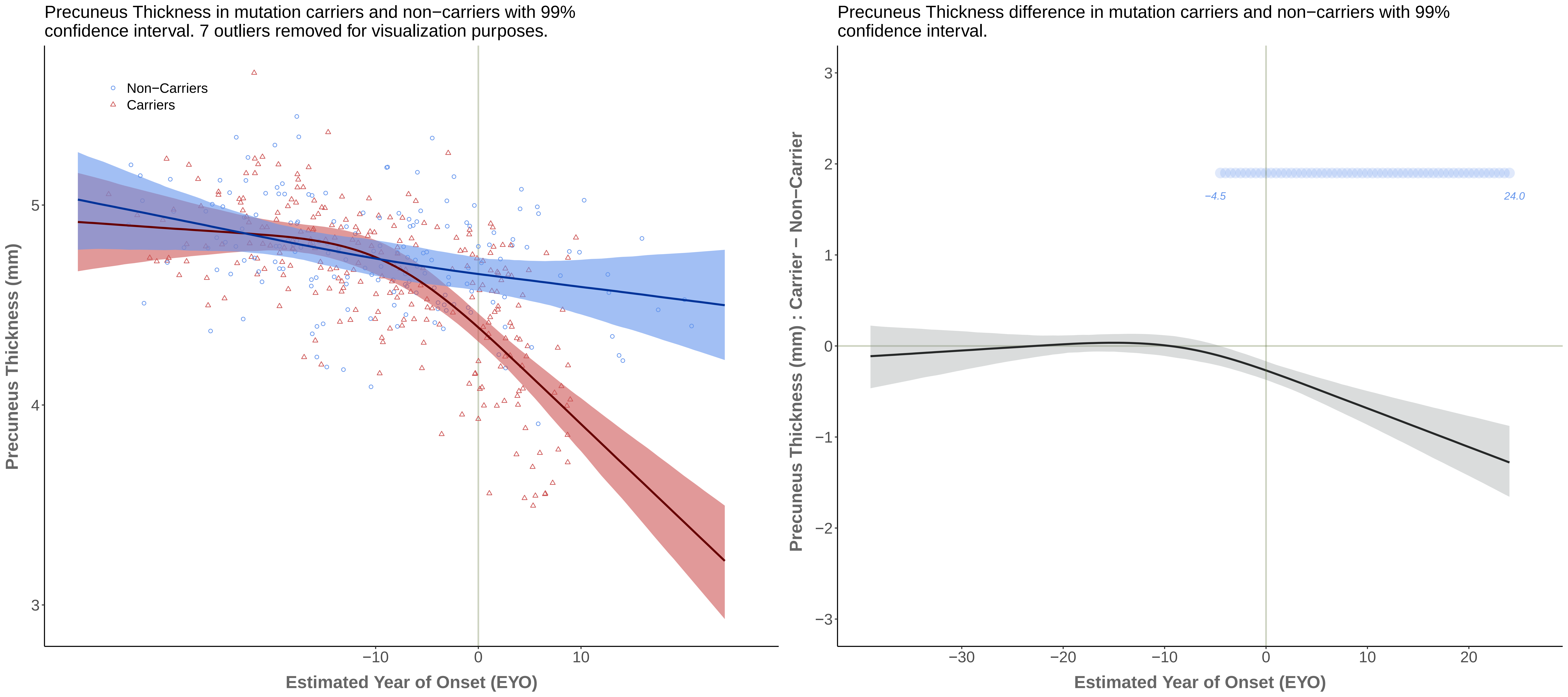
Precuneus Thickness in mutation carriers and non−carriers with 99% confidence interval. 7 outliers removed for visualization purposes.
Non−Carriers
Carriers
Precuneus Thickness (mm)
Estimated Year of Onset (EYO)
3
4
5
−10
0
10
Precuneus Thickness difference in mutation carriers and non−carriers with 99% confidence interval.
Precuneus Thickness (mm) : Carrier − Non−Carrier
Estimated Year of Onset (EYO)
−4.5
24.0
−3
−2
−1
0
1
2
3
−30
−20
−10
0
10
20

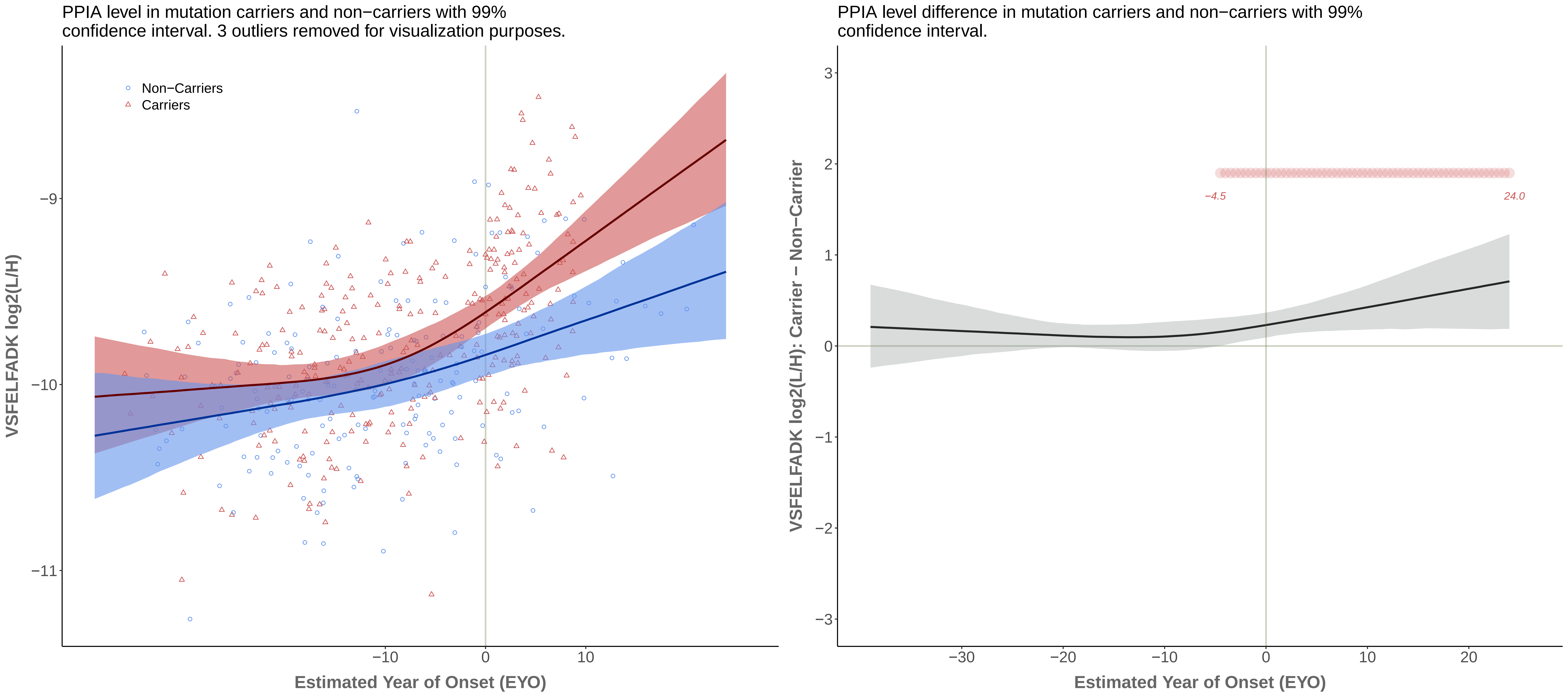
PPIA level in mutation carriers and non−carriers with 99% confidence interval. 3 outliers removed for visualization purposes.
Non−Carriers
Carriers
−9
−10
−11
VSFELFADK log2(L/H)
−10
0
10
Estimated Year of Onset (EYO)
PPIA level difference in mutation carriers and non−carriers with 99% confidence interval.
3
2
1
0
−1
−2
−3
−4.5
24.0
VSFELFADK log2(L/H): Carrier − Non−Carrier
−30
−20
−10
0
10
20
Estimated Year of Onset (EYO)

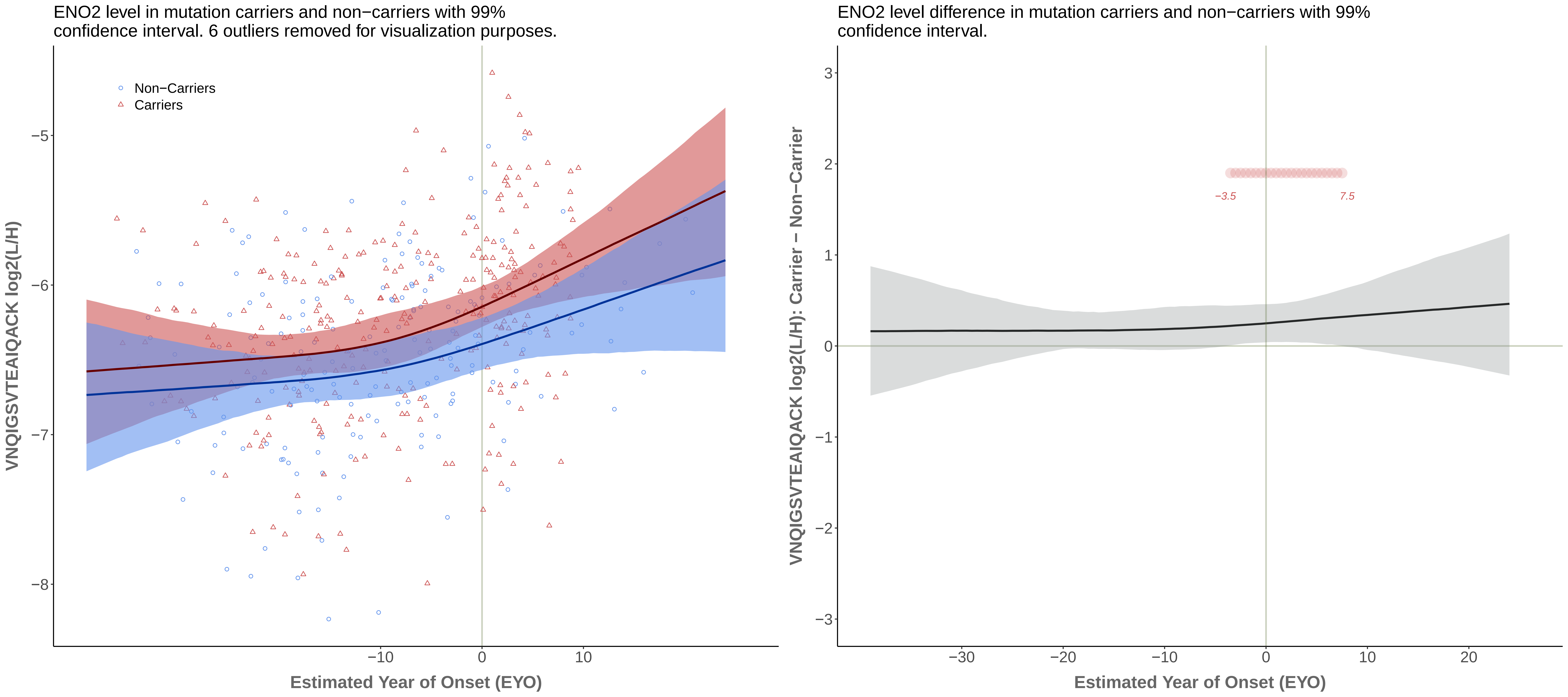

ENO2 level in mutation carriers and non−carriers with 99% confidence interval. 6 outliers removed for visualization purposes.
Non−Carriers
Carriers
−5
−6
−7
−8
VNQIGSVTEAIQACK log2(L/H)
−10
0
10
Estimated Year of Onset (EYO)
ENO2 level difference in mutation carriers and non−carriers with 99% confidence interval.
3
2
1
0
−1
−2
−3
−3.5
7.5
VNQIGSVTEAIQACK log2(L/H): Carrier − Non−Carrier
−30
−20
−10
0
10
20
Estimated Year of Onset (EYO)

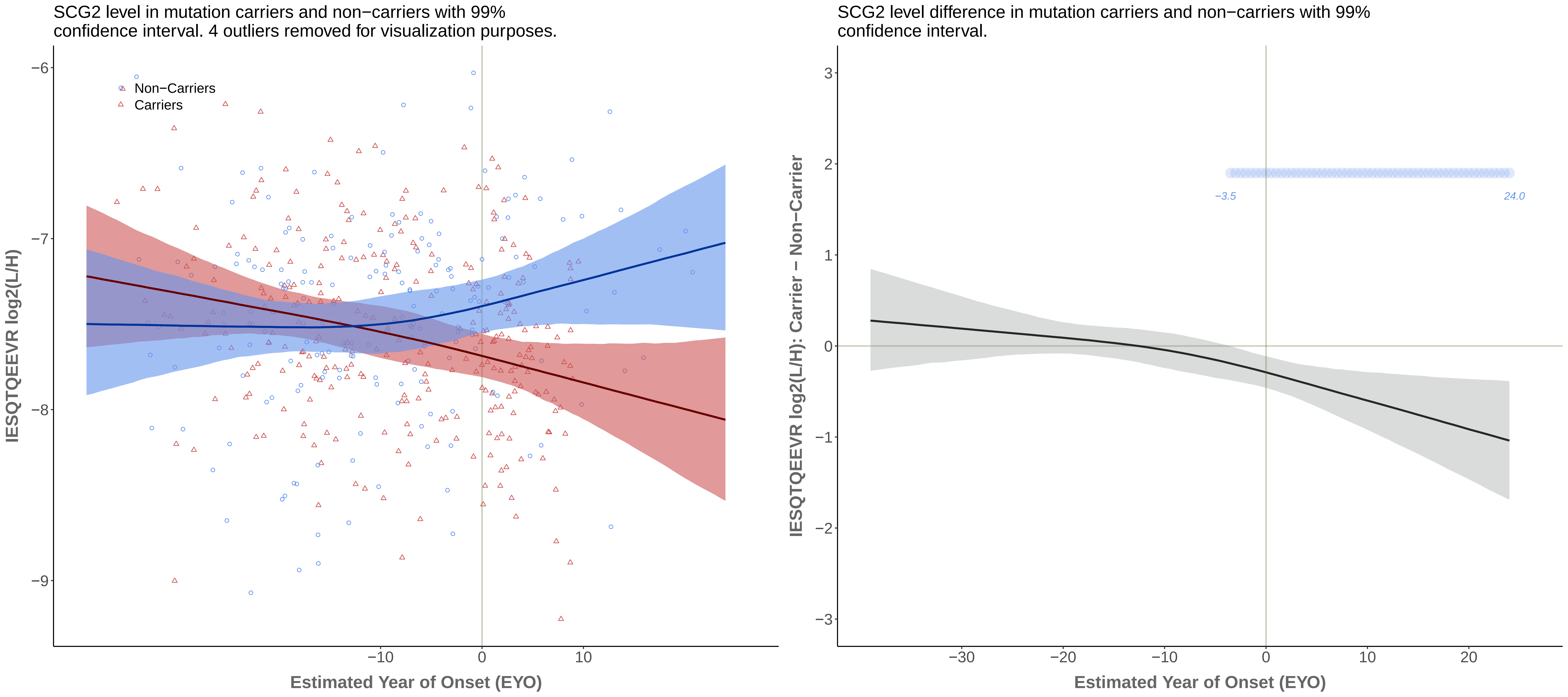

SCG2 level in mutation carriers and non−carriers with 99% confidence interval. 4 outliers removed for visualization purposes.
Non−Carriers
Carriers
IESQTQEEVR log2(L/H)
Estimated Year of Onset (EYO)
SCG2 level difference in mutation carriers and non−carriers with 99% confidence interval.
IESQTQEEVR log2(L/H): Carrier − Non−Carrier
−3.5
24.0
Estimated Year of Onset (EYO)

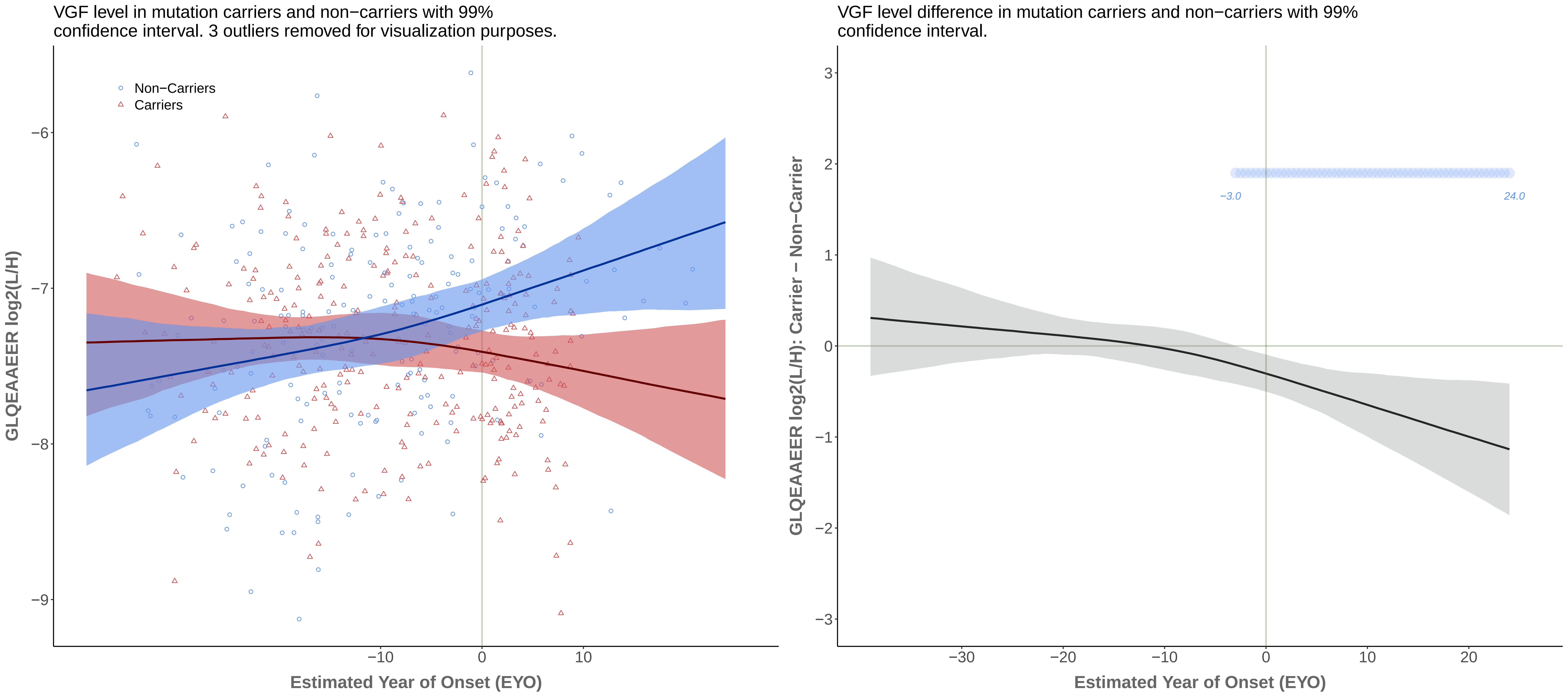
VGF level in mutation carriers and non−carriers with 99% confidence interval. 3 outliers removed for visualization purposes.
Non−Carriers
Carriers
GLQEAAEER log2(L/H)
−6
−7
−8
−9
−10
0
10
Estimated Year of Onset (EYO)
VGF level difference in mutation carriers and non−carriers with 99% confidence interval.
GLQEAAEER log2(L/H): Carrier − Non−Carrier
3
2
1
0
−1
−2
−3
−3.0
24.0
−30
−20
−10
0
10
20
Estimated Year of Onset (EYO)

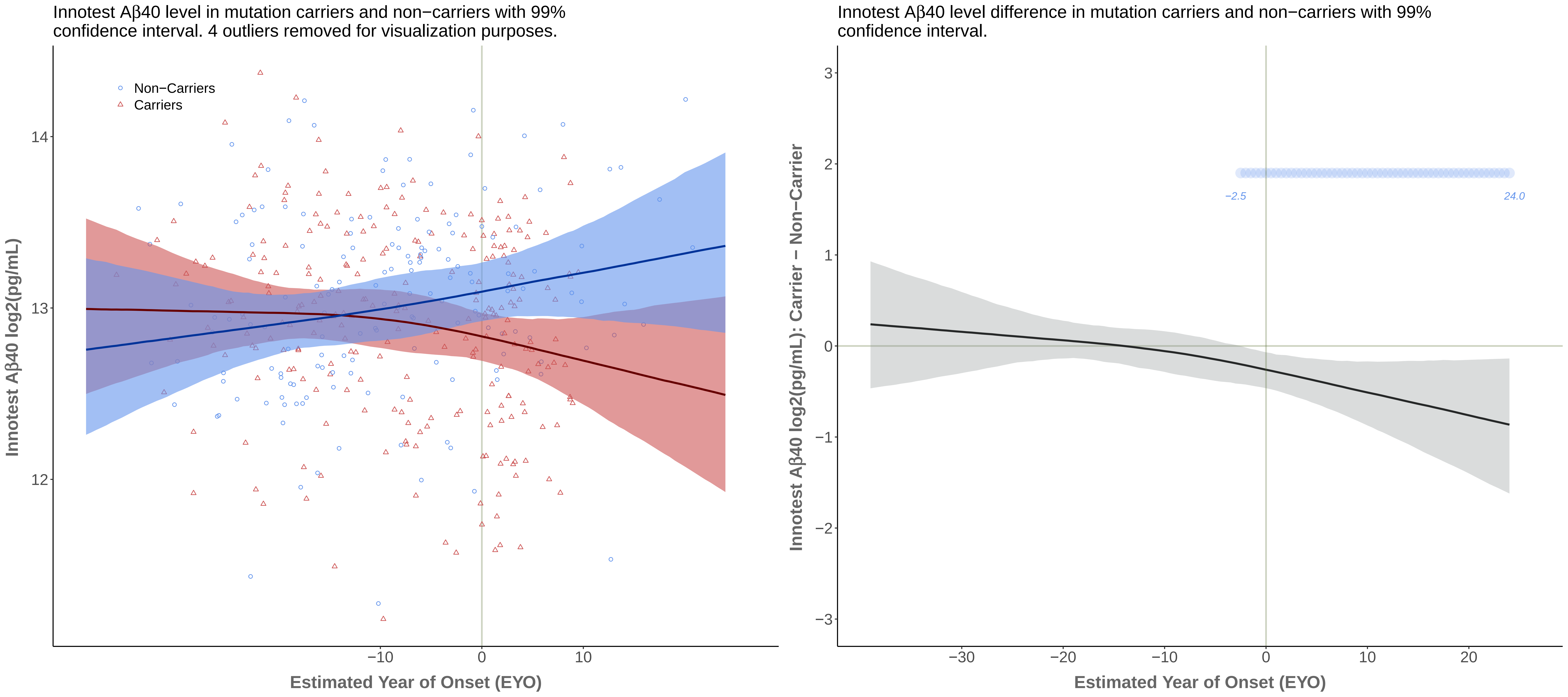
Innotest Aβ40 level in mutation carriers and non−carriers with 99% confidence interval. 4 outliers removed for visualization purposes.
Non−Carriers
Carriers
Innotest Aβ40 log2(pg/mL)
14
13
12
−10
0
10
Estimated Year of Onset (EYO)
Innotest Aβ40 level difference in mutation carriers and non−carriers with 99% confidence interval.
Innotest Aβ40 log2(pg/mL): Carrier − Non−Carrier
3
2
1
0
−1
−2
−3
−2.5
24.0
−30
−20
−10
0
10
20
Estimated Year of Onset (EYO)

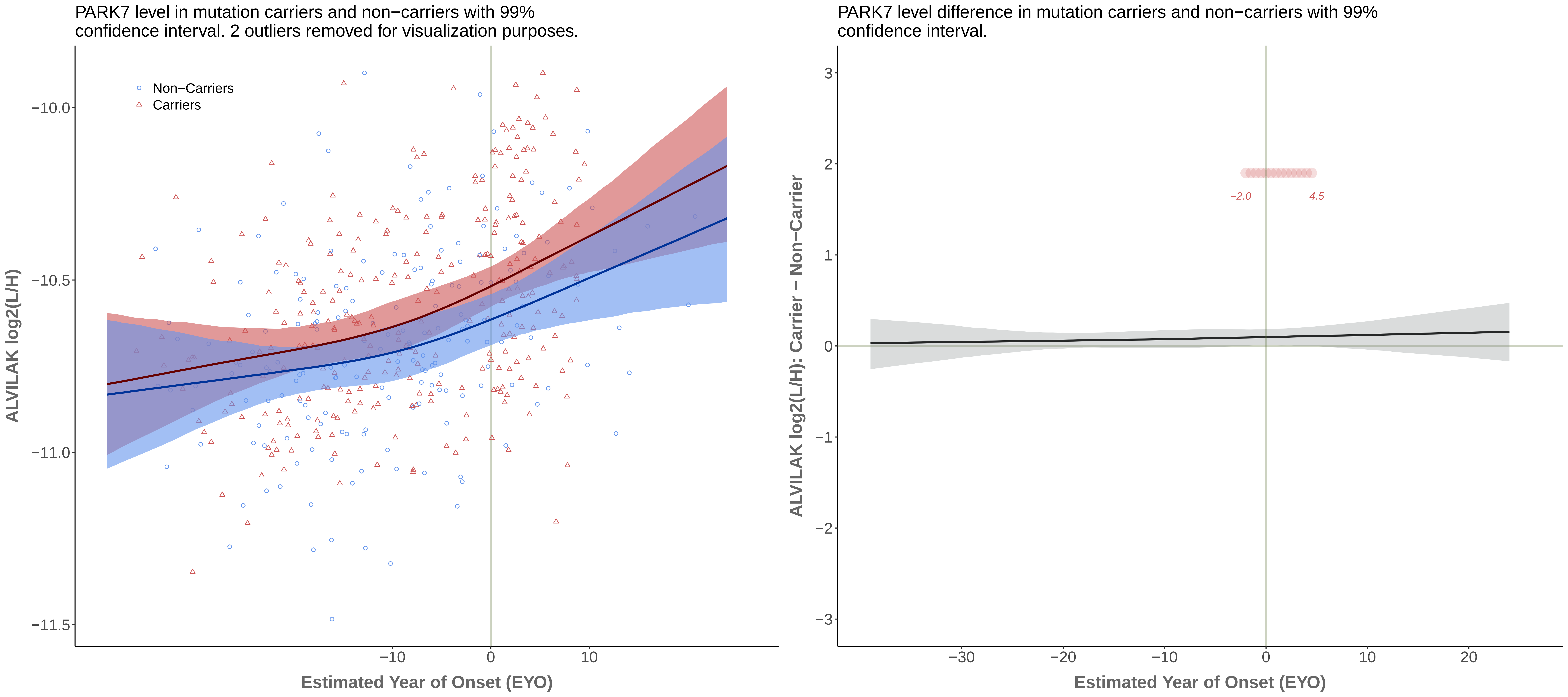
PARK7 level in mutation carriers and non−carriers with 99% confidence interval. 2 outliers removed for visualization purposes.
Non−Carriers
Carriers
−10.0
−10.5
−11.0
−11.5
ALVILAK log2(L/H)
−10
0
10
Estimated Year of Onset (EYO)
PARK7 level difference in mutation carriers and non−carriers with 99% confidence interval.
3
2
1
0
−1
−2
−3
ALVILAK log2(L/H): Carrier − Non−Carrier
−2.0
4.5
−30
−20
−10
0
10
20
Estimated Year of Onset (EYO)

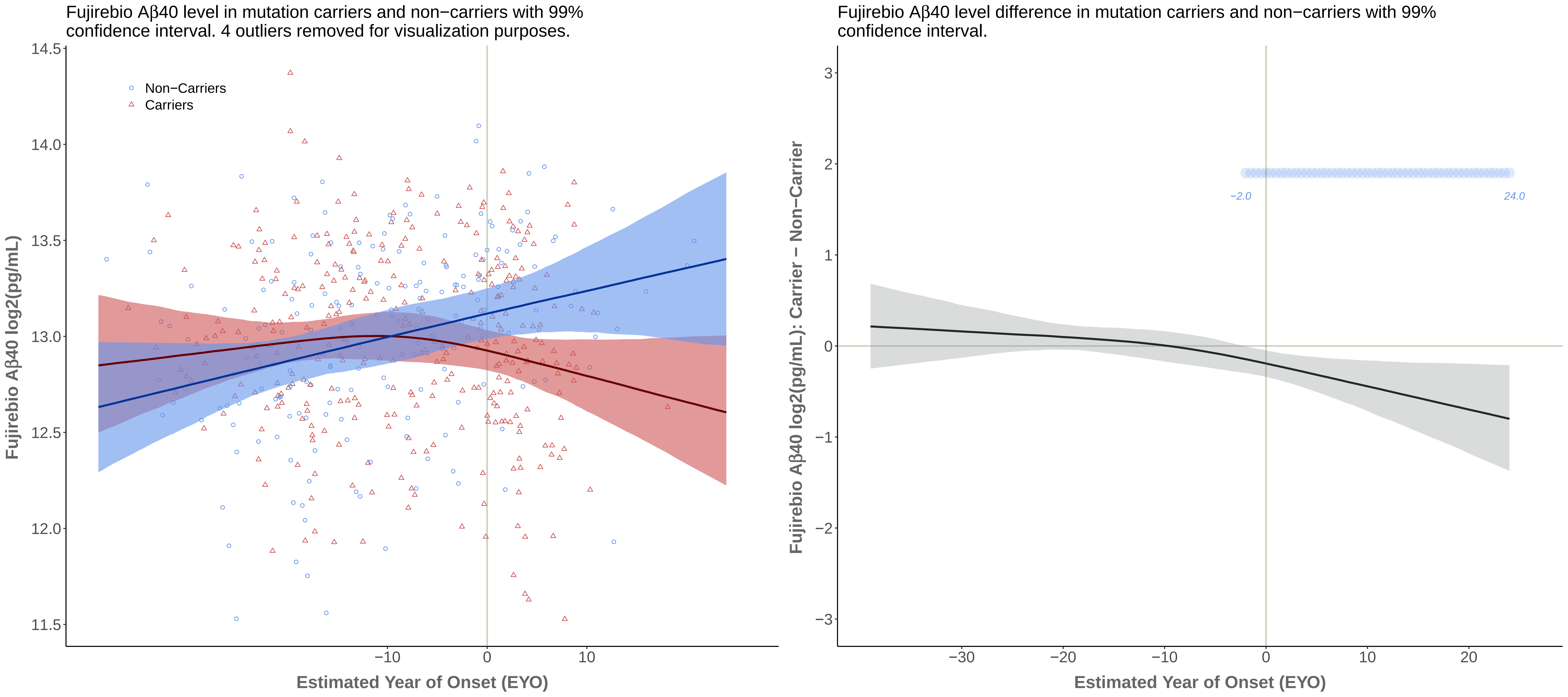

Fujirebio Aβ40 level in mutation carriers and non−carriers with 99% confidence interval. 4 outliers removed for visualization purposes.
Non−Carriers
Carriers
Fujirebio Aβ40 log2(pg/mL)
14.5
14.0
13.5
13.0
12.5
12.0
11.5
−10
0
10
Estimated Year of Onset (EYO)
Fujirebio Aβ40 level difference in mutation carriers and non−carriers with 99% confidence interval.
Fujirebio Aβ40 log2(pg/mL): Carrier − Non−Carrier
3
2
1
0
−1
−2
−3
−2.0
24.0
−30
−20
−10
0
10
20
Estimated Year of Onset (EYO)

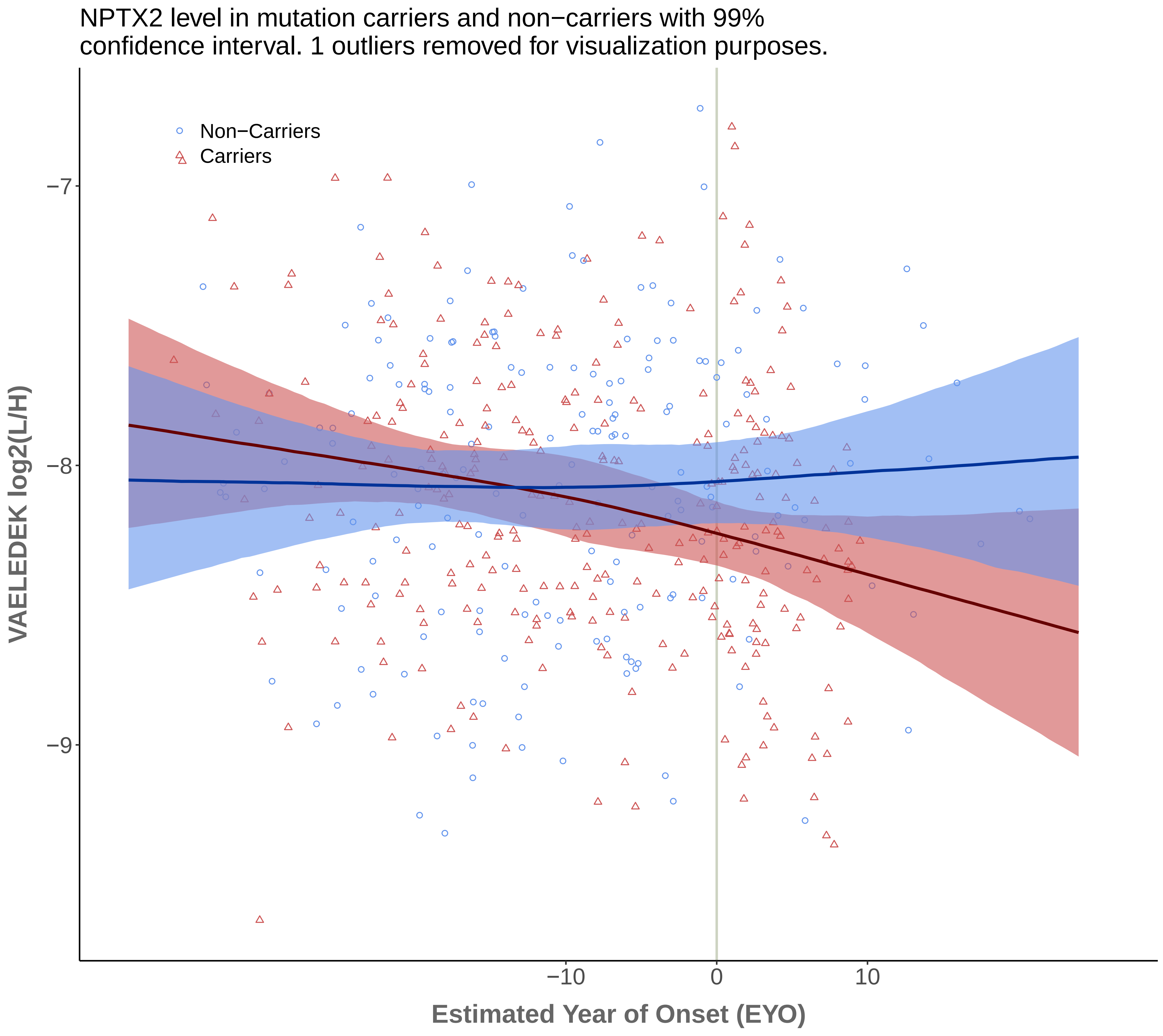
NPTX2 level in mutation carriers and non−carriers with 99%
confidence interval. 1 outliers removed for visualization purposes.
Non−Carriers
Carriers
−7
−8
−9
VAELEDEK log2(L/H)
−10
0
10
Estimated Year of Onset (EYO)

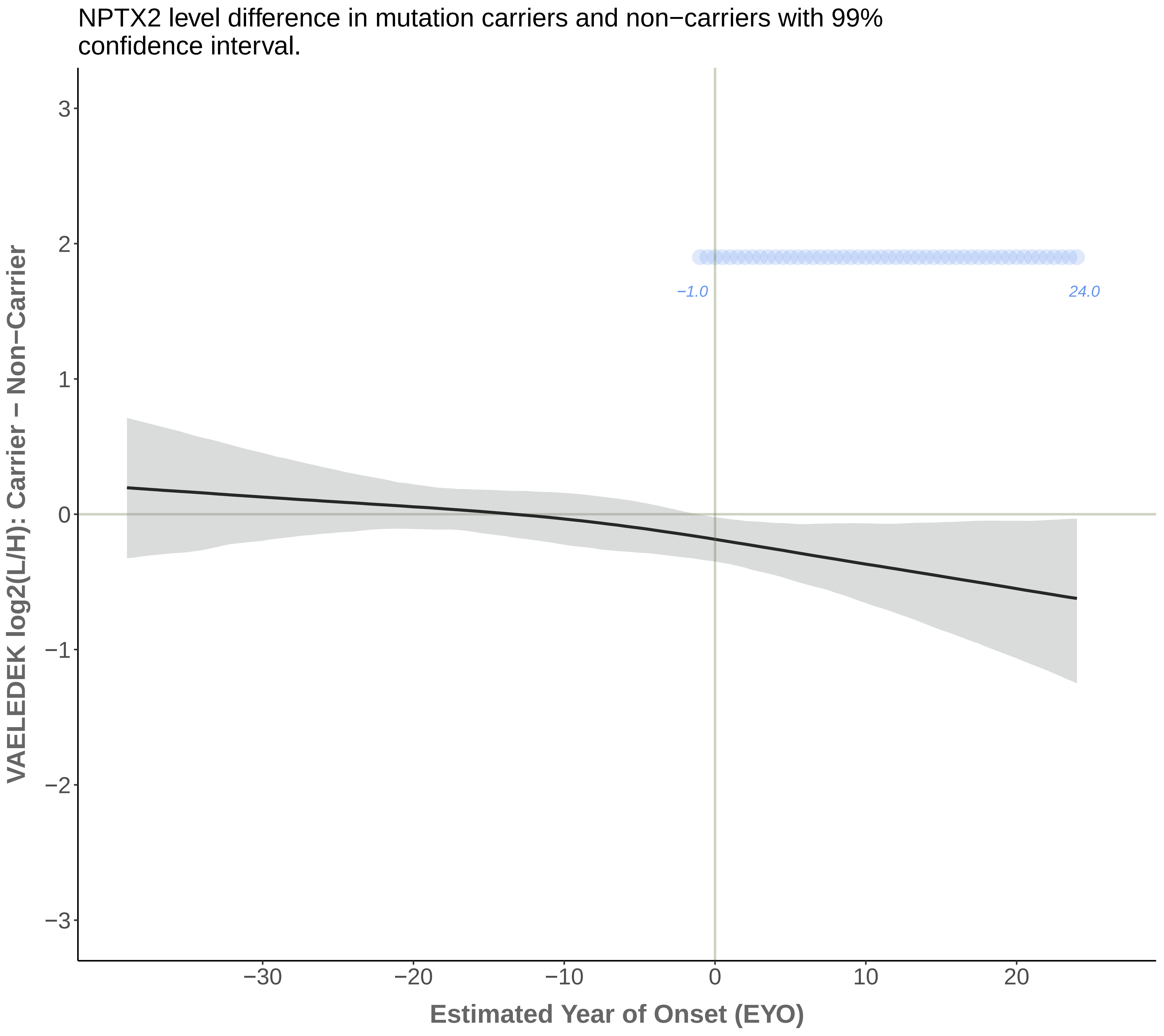
NPTX2 level difference in mutation carriers and non−carriers with 99%
confidence interval.
3
2
1
0
−1
−2
−3
−1.0
24.0
VAELEDEK log2(L/H): Carrier − Non−Carrier
−30
−20
−10
0
10
20
Estimated Year of Onset (EYO)

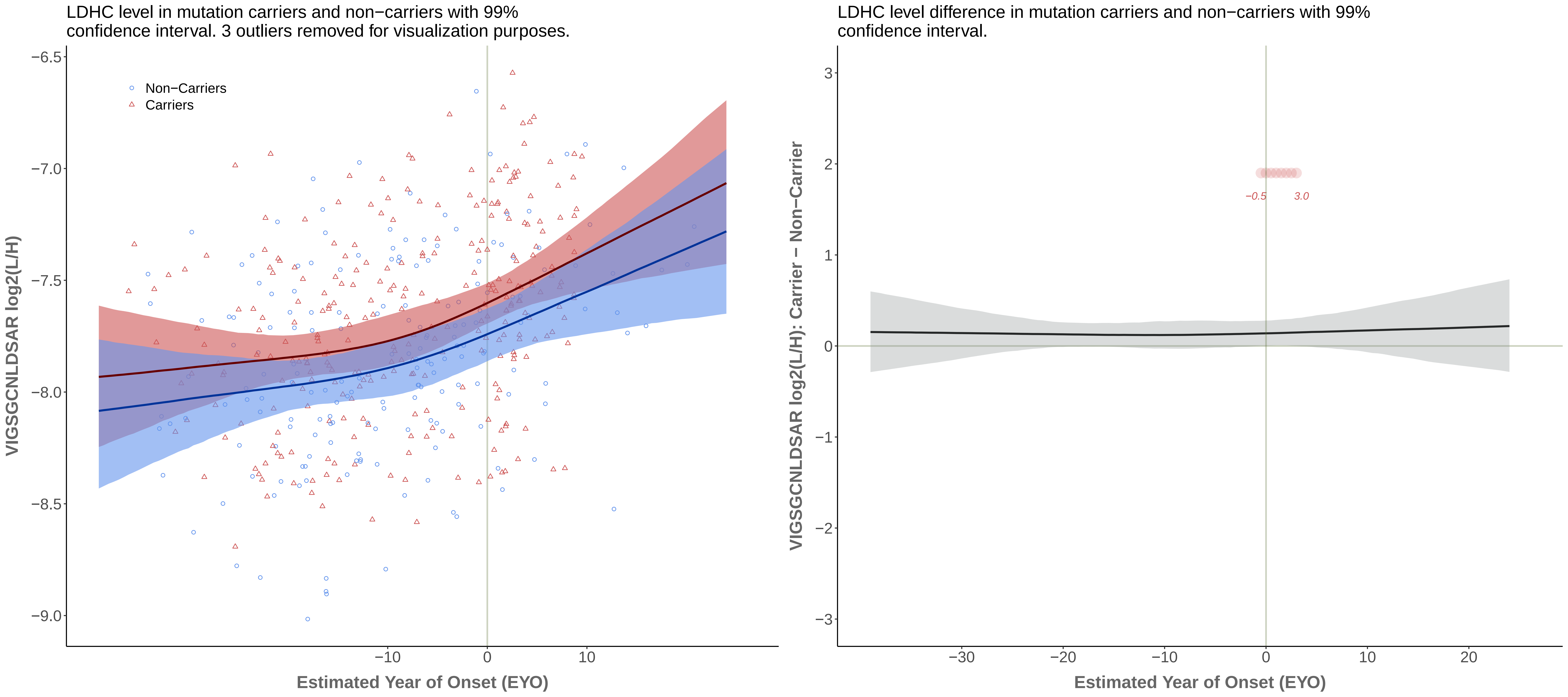
LDHC level in mutation carriers and non−carriers with 99% confidence interval. 3 outliers removed for visualization purposes.
Non−Carriers
Carriers
VIGSGCNLDSAR log2(L/H)
Estimated Year of Onset (EYO)
−6.5
−7.0
−7.5
−8.0
−8.5
−9.0
−10
0
10
LDHC level difference in mutation carriers and non−carriers with 99% confidence interval.
VIGSGCNLDSAR log2(L/H): Carrier − Non−Carrier
−0.5
3.0
3
2
1
0
−1
−2
−3
−30
−20
−10
0
10
20
Estimated Year of Onset (EYO)

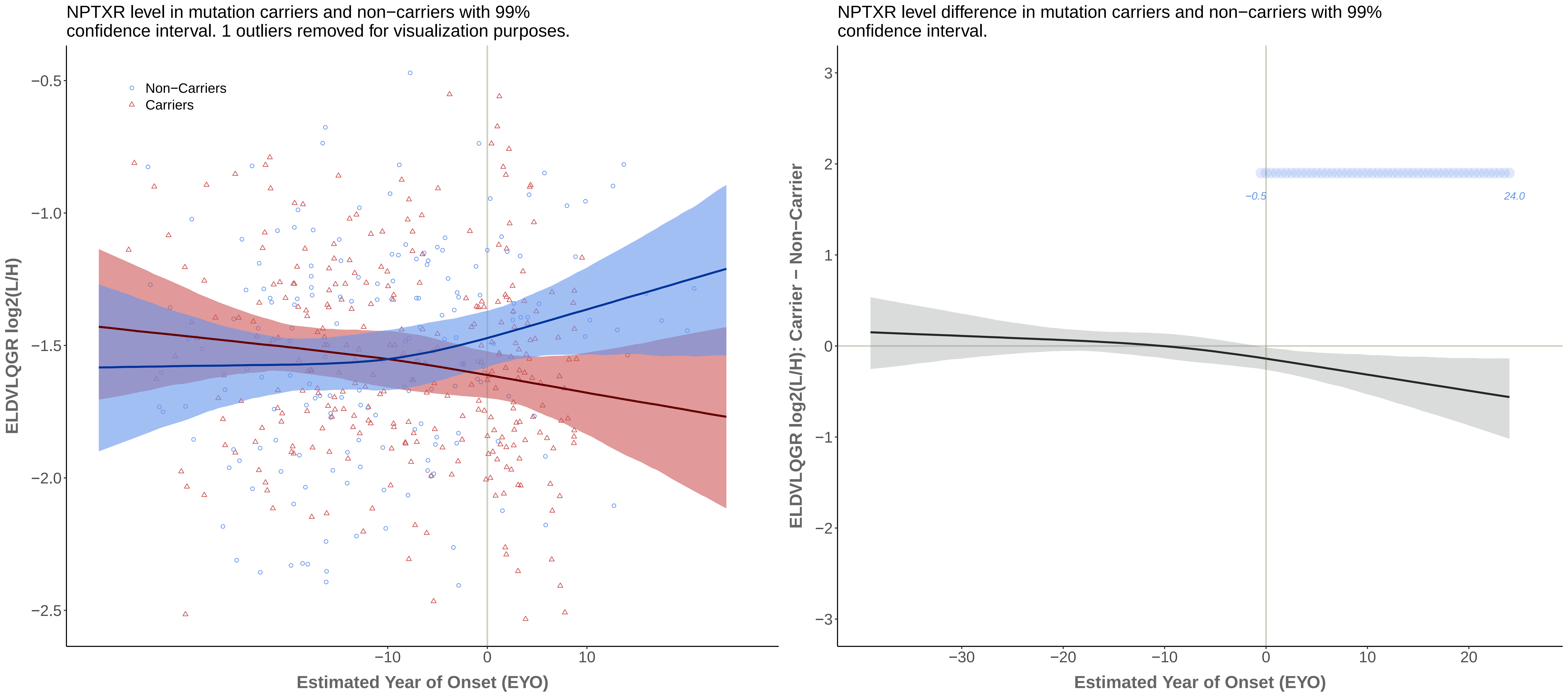
NPTXR level in mutation carriers and non−carriers with 99% confidence interval. 1 outliers removed for visualization purposes.
Non−Carriers
Carriers
ELDVLQGR log2(L/H)
Estimated Year of Onset (EYO)
NPTXR level difference in mutation carriers and non−carriers with 99% confidence interval.
ELDVLQGR log2(L/H): Carrier − Non−Carrier
−0.5
24.0
Estimated Year of Onset (EYO)

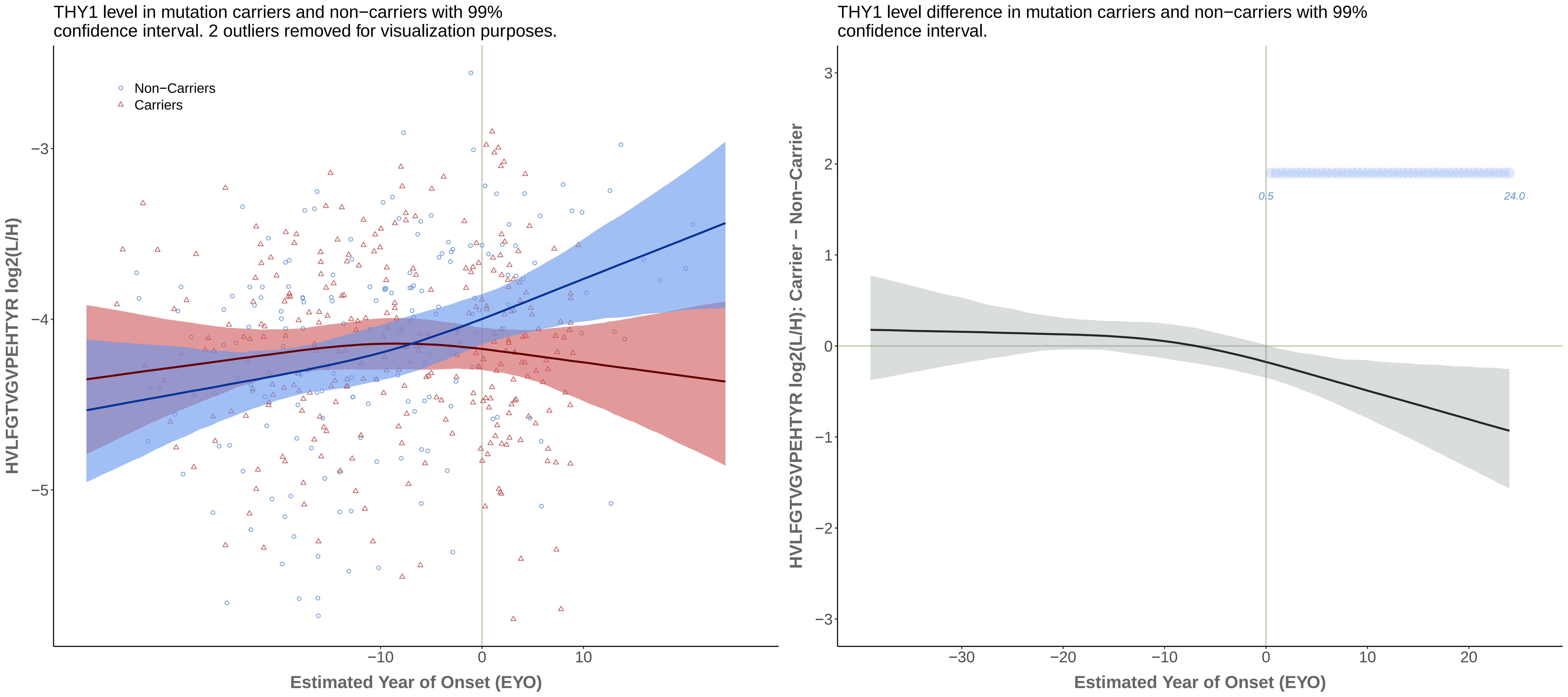
THY1 level in mutation carriers and non−carriers with 99% confidence interval. 2 outliers removed for visualization purposes.
Non−Carriers
Carriers
HVLFGTVGVPEHTYR log2(L/H)
−3
−4
−5
−10
0
10
Estimated Year of Onset (EYO)
THY1 level difference in mutation carriers and non−carriers with 99% confidence interval.
HVLFGTVGVPEHTYR log2(L/H): Carrier − Non−Carrier
3
2
1
0
−1
−2
−3
0.5
24.0
−30
−20
−10
0
10
20
Estimated Year of Onset (EYO)

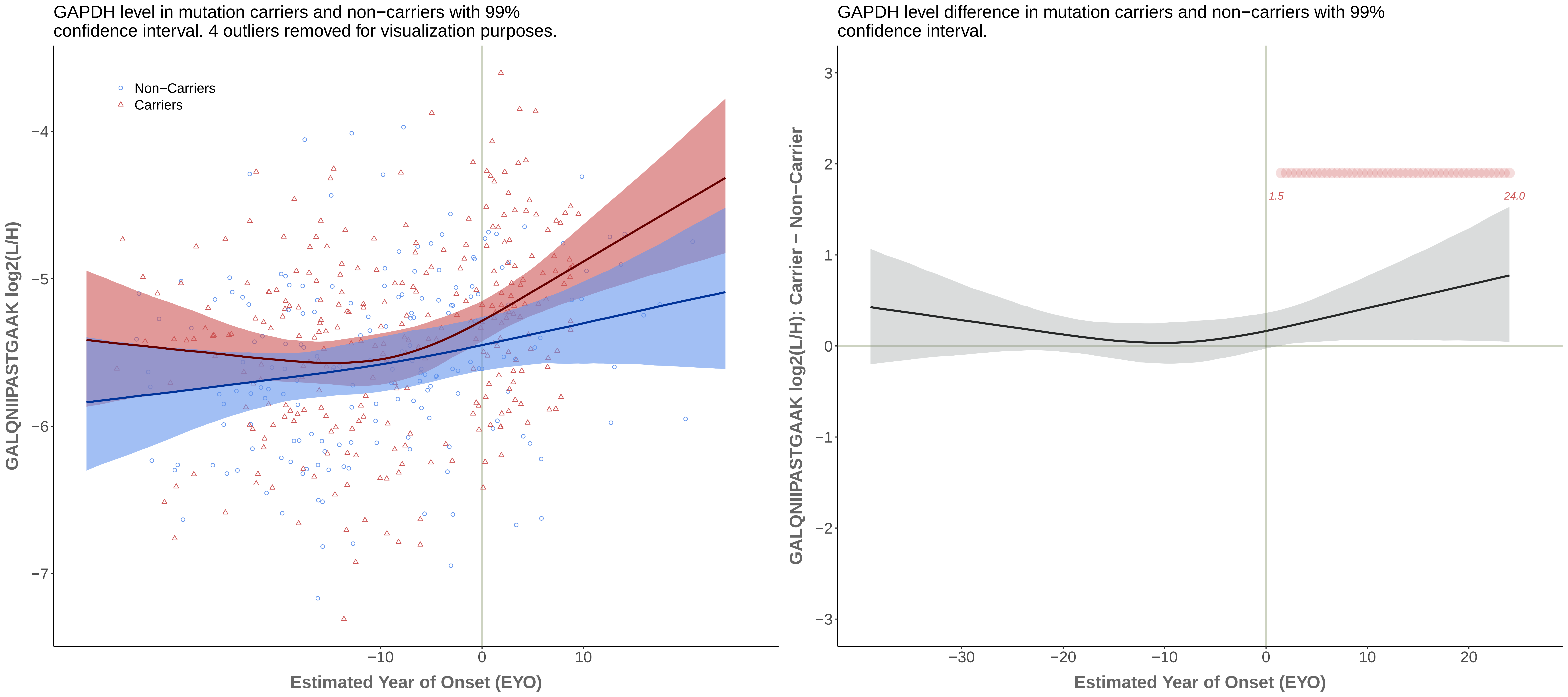

GAPDH level in mutation carriers and non−carriers with 99% confidence interval. 4 outliers removed for visualization purposes.
Non−Carriers
Carriers
GALQNIIPASTGAAK log2(L/H)
Estimated Year of Onset (EYO)
−4
−5
−6
−7
−10
0
10
GAPDH level difference in mutation carriers and non−carriers with 99% confidence interval.
GALQNIIPASTGAAK log2(L/H): Carrier − Non−Carrier
3
2
1
0
−1
−2
−3
1.5
24.0
−30
−20
−10
0
10
20
Estimated Year of Onset (EYO)

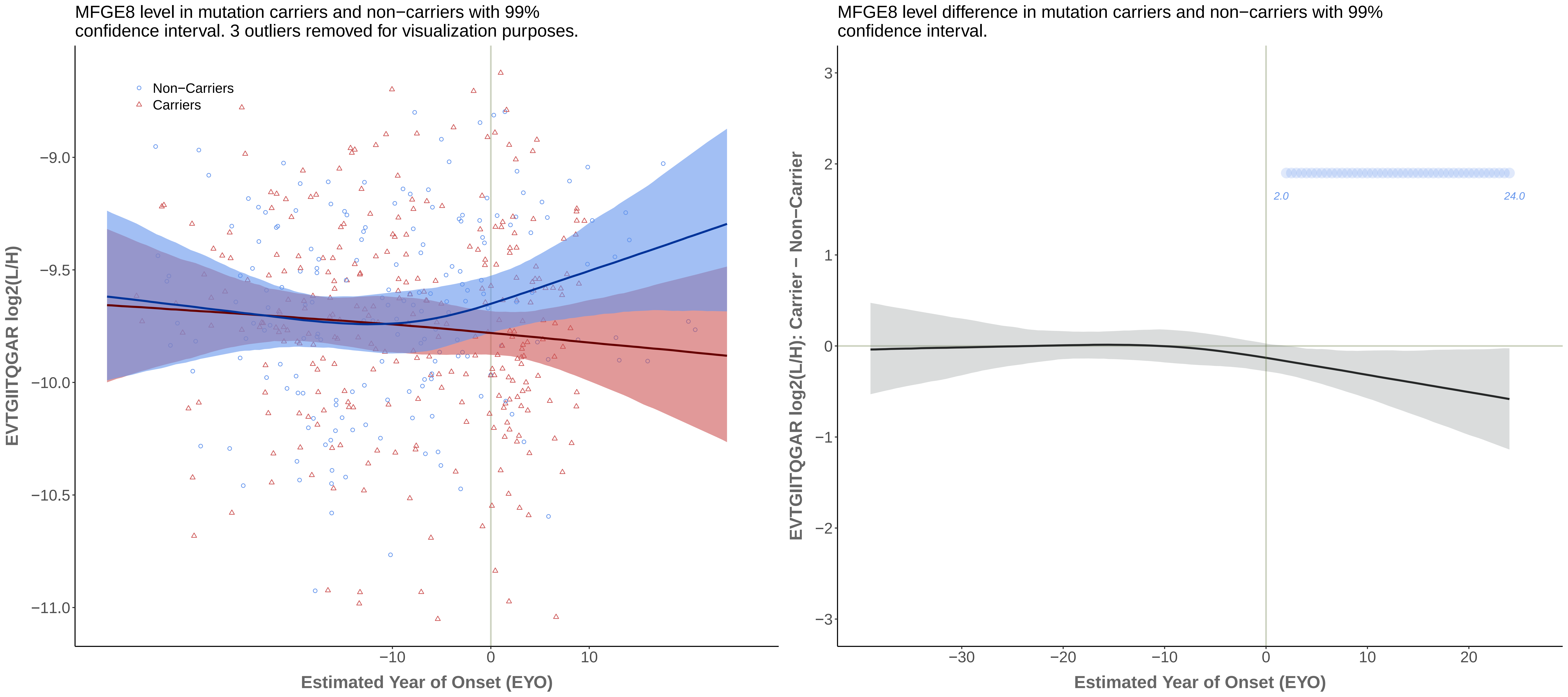

MFGE8 level in mutation carriers and non−carriers with 99% confidence interval. 3 outliers removed for visualization purposes.
Non−Carriers
Carriers
−9.0
−9.5
−10.0
−10.5
−11.0
EVTGIITQGAR log2(L/H)
−10
0
10
Estimated Year of Onset (EYO)
MFGE8 level difference in mutation carriers and non−carriers with 99% confidence interval.
3
2
1
0
−1
−2
−3
EVTGIITQGAR log2(L/H): Carrier − Non−Carrier
2.0
24.0
−30
−20
−10
0
10
20
Estimated Year of Onset (EYO)

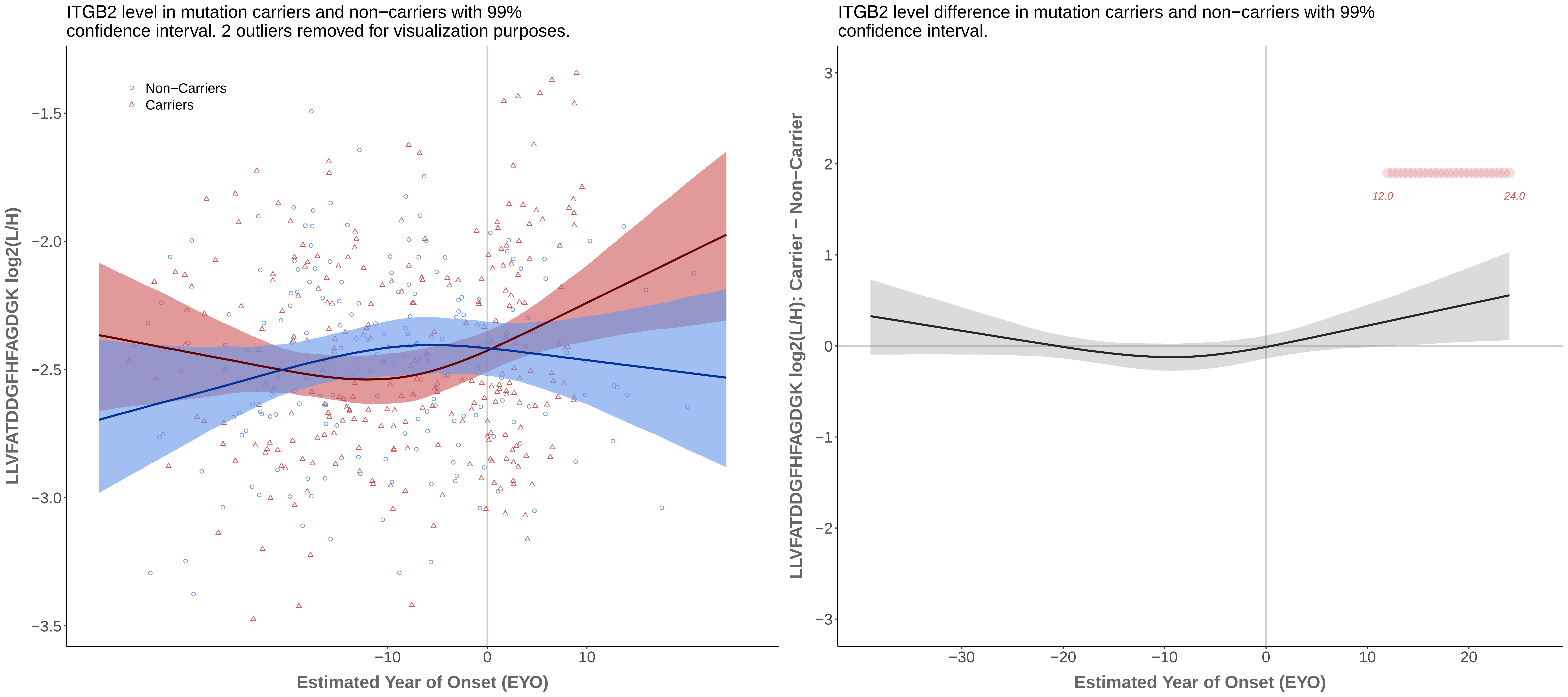

ITGB2 level in mutation carriers and non−carriers with 99% confidence interval. 2 outliers removed for visualization purposes.
Non−Carriers
Carriers
−1.5
−2.0
−2.5
−3.0
−3.5
LLVFATDDGFHFAGDGK log2(L/H)
−10
0
10
Estimated Year of Onset (EYO)
ITGB2 level difference in mutation carriers and non−carriers with 99% confidence interval.
3
2
1
0
−1
−2
−3
LLVFATDDGFHFAGDGK log2(L/H): Carrier − Non−Carrier
12.0
24.0
−30
−20
−10
0
10
20
Estimated Year of Onset (EYO)

Probability of having Global CDR standard score of 0 in mutation carriers and non−carriers
with 99% confidence interval. 14 outliers removed for visualization purposes.

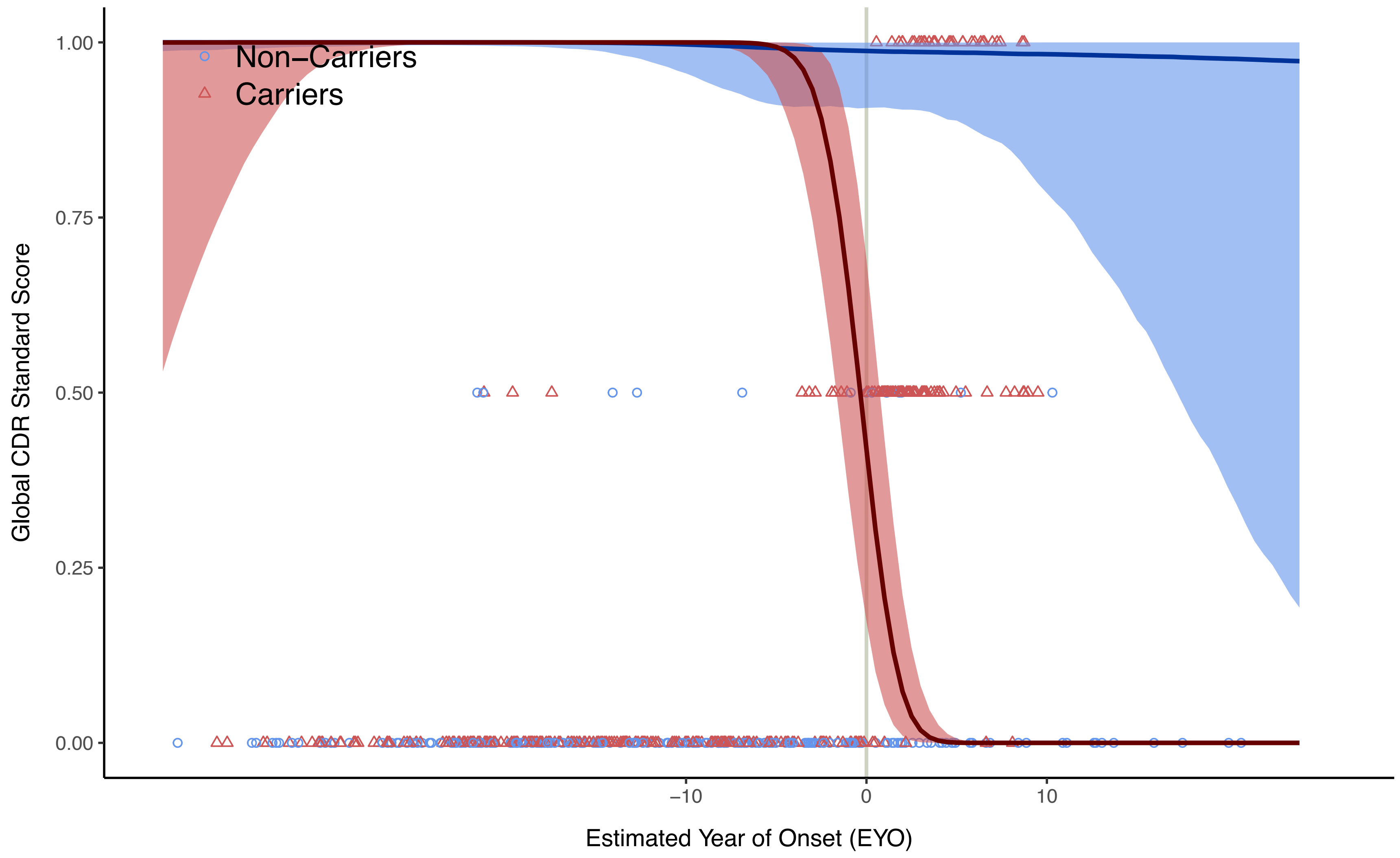

Probability of having Global CDR standard score of 0.5 in mutation carriers and non−carriers
with 99% confidence interval. 14 outliers removed for visualization purposes.

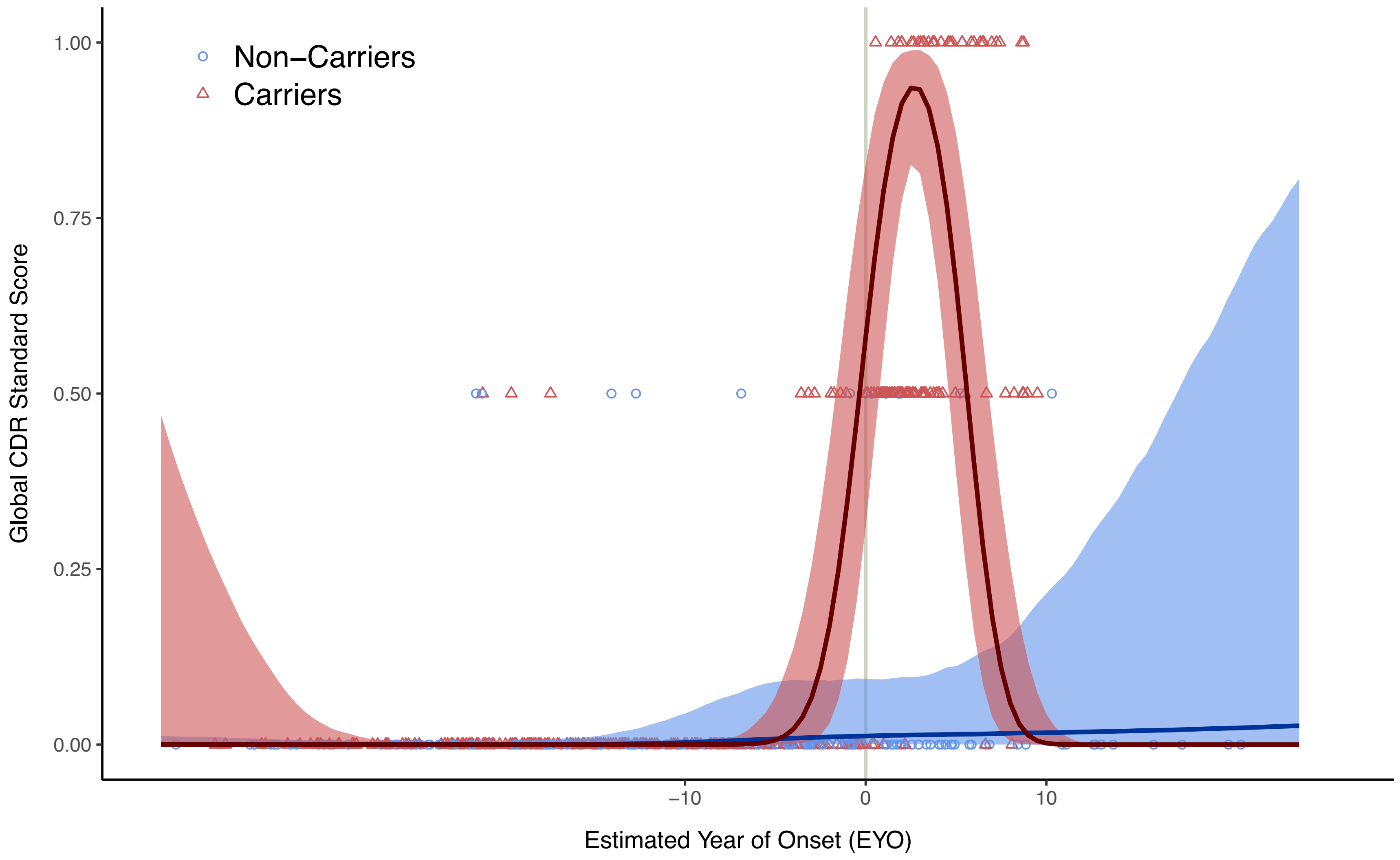

Probability of having Global CDR standard score greater than or equal to 1 in mutation carriers
and non−carriers with 99% confidence interval. 14 outliers removed for visualization purposes.

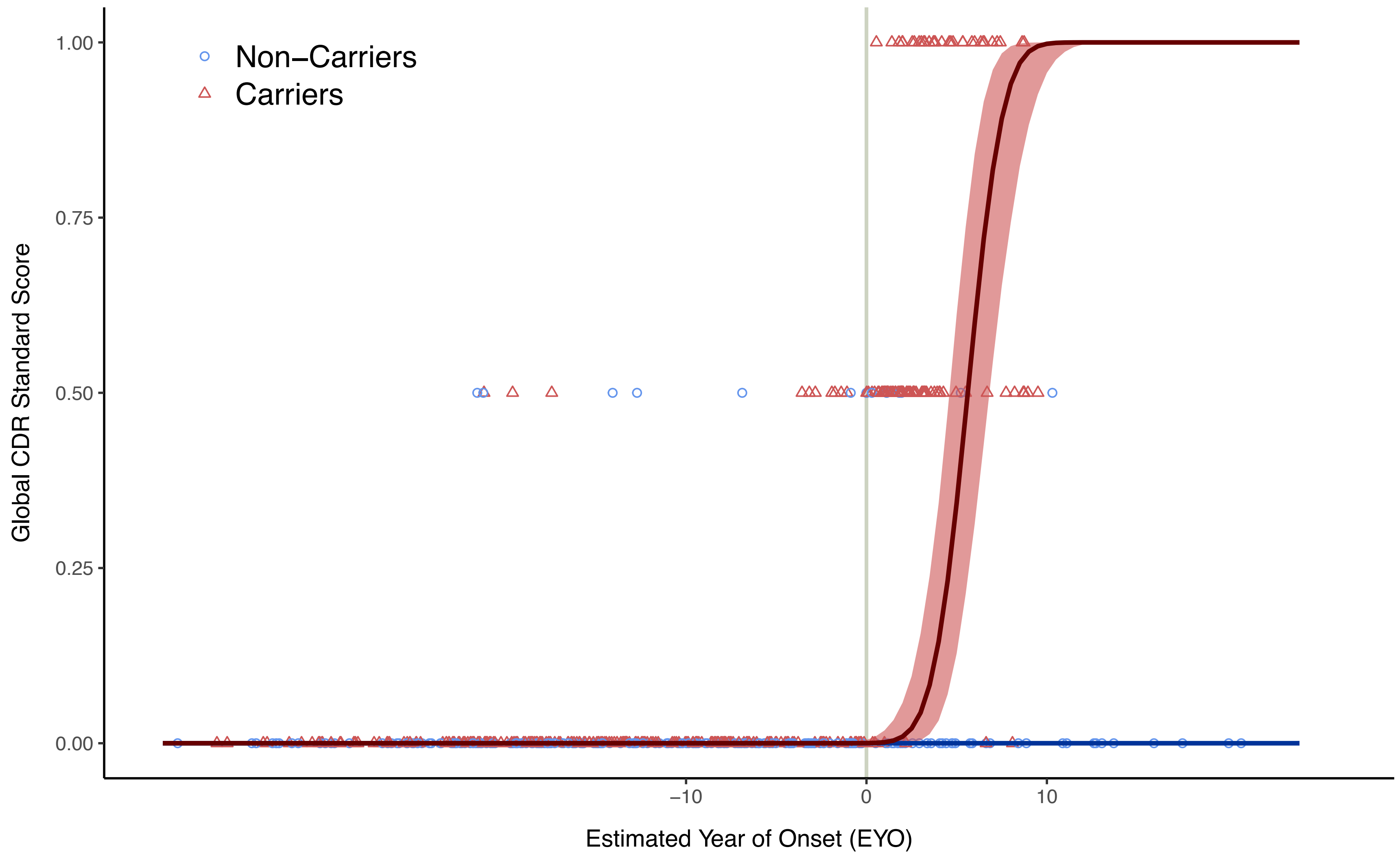

Probability of having MMSE < 19 in mutation carriers and non−carriers with 99% confidence interval.
17 outliers removed for visualization purposes.

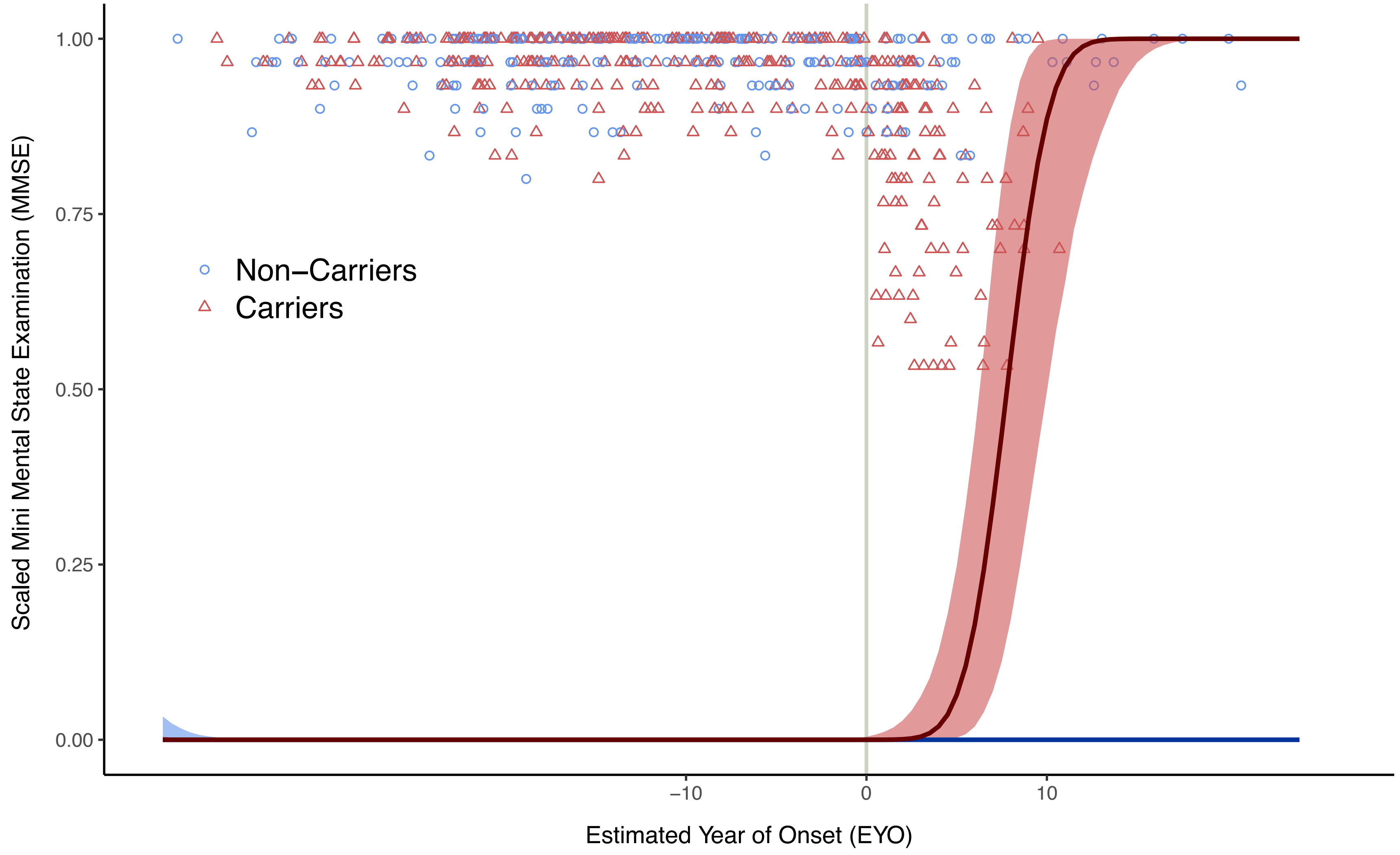

Probability of having MMSE between 19 and 24 (inclusive) in mutation carriers and non−carriers with
99% confidence interval. 17 outliers removed for visualization purposes.

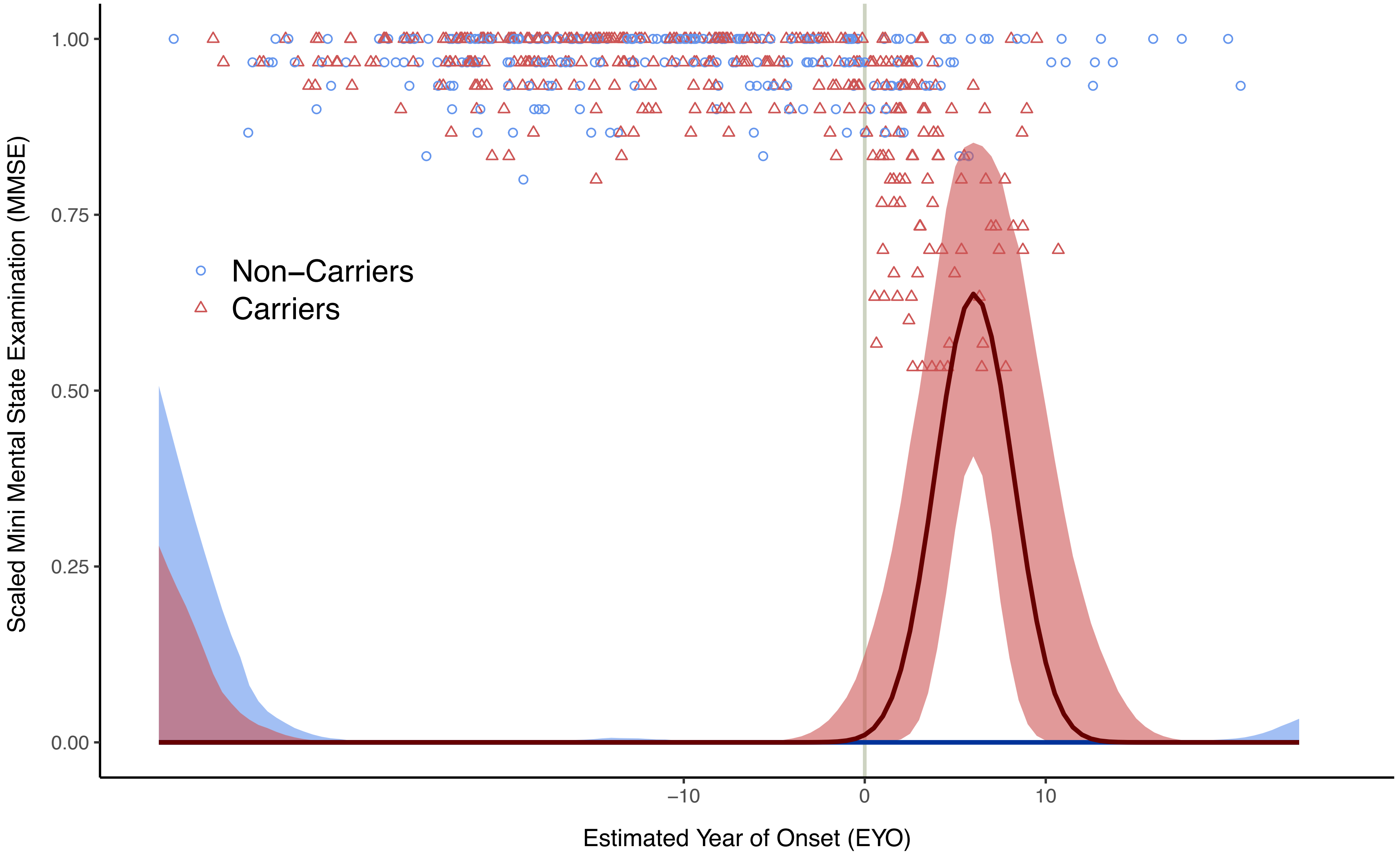

Probability of having MMSE >24 in mutation carriers and non−carriers with 99% confidence interval.
17 outliers removed for visualization purposes.

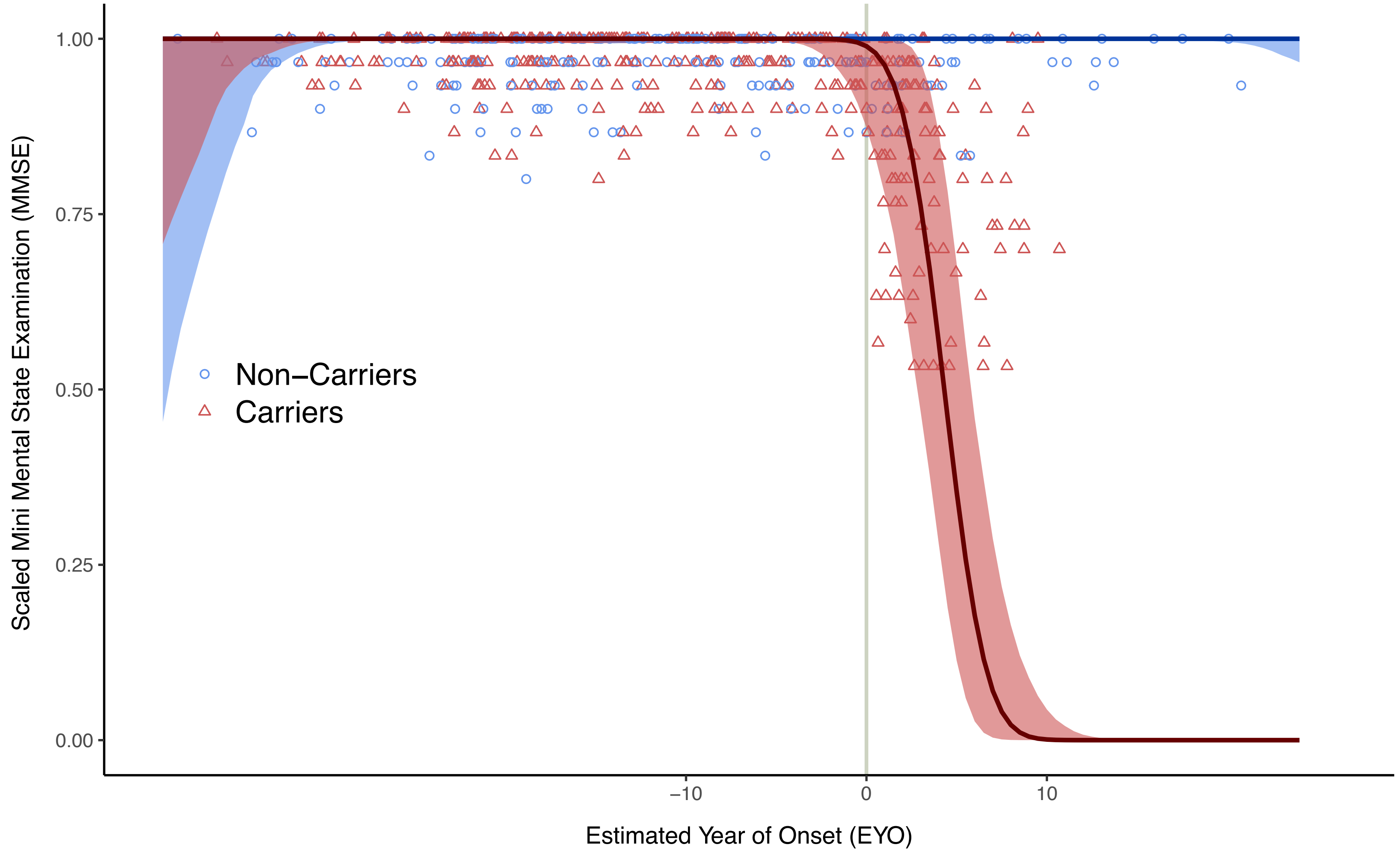

Biomarker Levels by Estimated Year of Disease Onset in ADAD. Periods of significant difference between carriers and non-carriers are highlighted in the difference plot on the right (red indicates significantly increased levels in carriers, blue indicates significantly decreased levels in carriers). Shaded areas represent the 99% credible interval. L/H, ratio of endogenous peptide signal (light) to the isotopically labeled standard peptide signal (heavy).
