# Supplementary material for: Cerebrospinal fluid proteomics define the natural history of autosomal dominant Alzheimer’s disease
Source: Nat Med. 2023 Aug 7;29(8):1979–88. doi: 10.1038/s41591-023-02476-4 (PMC10427428; doi:10.1038/s41591-023-02476-4)
Supplement: Supplementary file 2 — Reporting Summary [file 41591_2023_2476_MOESM2_ESM.pdf]

Corresponding author(s): Erik C.B. Johnson

Last updated by author(s): 06-14-2023

Reporting Summary

Nature Portfolio wishes to improve the reproducibility of the work that we publish. This form provides structure for consistency and transparency in reporting. For further information on Nature Portfolio policies, see our Editorial Policies and the Editorial Policy Checklist.

Statistics

For all statistical analyses, confirm that the following items are present in the figure legend, table legend, main text, or Methods section.

n/a	Confirmed	
☐	☒	The exact sample size (*n*) for each experimental group/condition, given as a discrete number and unit of measurement
☐	☒	A statement on whether measurements were taken from distinct samples or whether the same sample was measured repeatedly
☐	☒	The statistical test(s) used AND whether they are one- or two-sided<br>*Only common tests should be described solely by name; describe more complex techniques in the Methods section.*
☐	☒	A description of all covariates tested
☐	☒	A description of any assumptions or corrections, such as tests of normality and adjustment for multiple comparisons
☐	☒	A full description of the statistical parameters including central tendency (e.g. means) or other basic estimates (e.g. regression coefficient) AND variation (e.g. standard deviation) or associated estimates of uncertainty (e.g. confidence intervals)
☐	☒	For null hypothesis testing, the test statistic (e.g. *F*, *t*, *r*) with confidence intervals, effect sizes, degrees of freedom and *P* value noted<br>*Give P values as exact values whenever suitable.*
☐	☒	For Bayesian analysis, information on the choice of priors and Markov chain Monte Carlo settings
☐	☒	For hierarchical and complex designs, identification of the appropriate level for tests and full reporting of outcomes
☒	☐	Estimates of effect sizes (e.g. Cohen's *d*, Pearson's *r*), indicating how they were calculated

Our web collection on statistics for biologists contains articles on many of the points above.

Software and code

Policy information about availability of computer code

Data collection	Skyline-Daily v22.2.1.351
Data analysis	In-house code was used to curate the data, complete data tables, and create figures with Python v3.10.8, scipy v1.9.3, seaborn v0.12.1, and matplotlib v3.6.2.<br><br>Statistical data analysis was performed in R (4.1.2). The Bayesian generalized linear models were implemented using the open-source R package rstanarm (2.21.3). The Bayesian mixed-effects ordinal regression model was implemented using the open-source R package brms (2.18.0). Figures 1 was plotted using ggplot2 (3.3.6).<br><br>Logistic regression and receiver operating characteristics were performed using sklearn 0.24.2 libraries and custom Python v3.9 code, which is available upon request.

For manuscripts utilizing custom algorithms or software that are central to the research but not yet described in published literature, software must be made available to editors and reviewers. We strongly encourage code deposition in a community repository (e.g. GitHub). See the Nature Portfolio guidelines for submitting code & software for further information.

Data

Policy information about availability of data

All manuscripts must include a data availability statement. This statement should provide the following information, where applicable:

- Accession codes, unique identifiers, or web links for publicly available datasets
- A description of any restrictions on data availability
- For clinical datasets or third party data, please ensure that the statement adheres to our policy

DIAN trait data are available through request. Instructions can be found at https://dian.wustl.edu/our-research/for-investigators/dian-observational-study-investigator-resources/data-request-terms-and-instructions/. Source data are under controlled access to protect mutation carrier confidentiality. Data requests will be reviewed based on scientific merit and feasibility, appropriateness of the investigator's qualifications and resources to protect the data, and appropriateness to DIAN goals/themes. De-identified DIAN data will be made available to investigators to conduct analyses after approval by the PI and the relevant DIAN Core Leader. The data request form can be found at https://dian.wustl.edu/our-research/for-investigators/dian-observational-study-investigator-resources/data-request-form/. Data access requests are typically processed within 30-60 days.

Human research participants

Policy information about studies involving human research participants and Sex and Gender in Research.

Reporting on sex and gender	Sex as a predictor was analyzed in an ad-hoc analysis along with APOE4 status, but was not selected in our final model because it did not have a significant effect on the results.
Population characteristics	Please see Table 1 and Supplementary Table 1 for study population characteristics
Recruitment	Individuals are eligible to be enrolled in DIAN if they are adult children (18 years or older) of a clinically affected parent in an ADAD family in which the parent (or consanguineous relative) has a known mutation causing symptomatic AD. The primary sources of recruitment into DIAN are the ADAD families that are identified by DIAN performance sites located around the world, including kindreds that were previously established and kindreds that have emerged in response to publicity about DIAN. The DIAN Expanded Registry website serves as another recruitment tool for DIAN. Other recruitment sources include the National Centralized Repository for Alzheimer's Disease and Related Dementias (NCRAD), physicians with patients with positive genetic testing results, and individuals from ADAD families who seek information from relevant nonprofit organizations (the Alzheimer's Association; the Alzheimer Research Forum) and the NIA's Alzheimer's Disease Education and Referral Center. For further information on inclusion criteria and study sites, see https://dian.wustl.edu/our-research/observational-study/dian-observational-study-participation/.
Ethics oversight	The institutional review board at Washington University in St. Louis provided supervisory review and human studies approval. All study participants provided informed consent.

Note that full information on the approval of the study protocol must also be provided in the manuscript.

Field-specific reporting

Please select the one below that is the best fit for your research. If you are not sure, read the appropriate sections before making your selection.

☒ Life sciences ☐ Behavioural & social sciences ☐ Ecological, evolutionary & environmental sciences

For a reference copy of the document with all sections, see nature.com/documents/nr-reporting-summary-flat.pdf

Life sciences study design

All studies must disclose on these points even when the disclosure is negative.

Sample size	The first measurement for each trait from all available subjects was analyzed to achieve maximal statistical power to detect differences between mutation carriers and non-carriers.
Data exclusions	Measurements greater than five standard deviations from the mean after log2 transformation were considered technically suspect and removed before analysis. Exclusion criteria were established post-hoc after inspection of the data distributions. A very small fraction of the data were excluded, and inclusion of the outlier data points did not affect the outcome of the analysis. SRM measurements from two subjects were removed based on poor sample quality as determined by light peptide signal intensity.
Replication	Replication was not performed due to the rare nature of ADAD and the difficulty of establishing sufficiently large and well-phenotyped cohorts for study
Randomization	Subject-level randomization was not performed due to the design of the study. Co-variates, particularly genetic relatedness, were controlled for in the statistical model as described in Methods.

Blinding

Investigators were blinded to mutation carrier status during data acquisition to the extent possible. Analysis was necessarily performed unblinded to mutation carrier status on de-identified data so that participants could be correctly assigned to the mutation carrier or non-carrier group for comparison of groups differences across measures.

Reporting for specific materials, systems and methods

We require information from authors about some types of materials, experimental systems and methods used in many studies. Here, indicate whether each material, system or method listed is relevant to your study. If you are not sure if a list item applies to your research, read the appropriate section before selecting a response.

Materials & experimental systems

n/a	Involved in the study	
☐	☒	Antibodies
☒	☐	Eukaryotic cell lines
☒	☐	Palaeontology and archaeology
☒	☐	Animals and other organisms
☒	☐	Clinical data
☒	☐	Dual use research of concern

Methods

n/a	Involved in the study	
☒	☐	ChIP-seq
☒	☐	Flow cytometry
☐	☒	MRI-based neuroimaging

Antibodies

Antibodies used

ELISA measurements of Abeta, Tau, and pTau were obtained using the Luminex, Fujirebio, and Innotest platforms as described in Bateman, R.J., et al. The New England journal of medicine 367, 795-804 (2012). Plasma pTau181 and NEFL ELISA measurements were obtained on the Simoa HD-1 platform as described in Preische, O., et al. Nat Med 25, 277-283 (2019). c-sTREM2 measurements were obtained on the Meso Scale Discovery platform as described in Morenas-Rodriguez, E., et al. Lancet Neurol 21, 329-341 (2022). PRGN measurements were obtained on the Simoa HD-1 platform as described in Suarez-Calvet, M., et al. EMBO Mol Med 10 (2018).

Validation

Please see aforementioned studies for further details

Magnetic resonance imaging

Experimental design

Design type

MRI and PET imaging acquisition in DIAN has been described in detail in McKay, N.S., et al. Neuroimaging within the Dominantly Inherited Alzheimer's Network (DIAN): PET and MRI. bioRxiv, 2022.2003.2025.485799 (2022), https://www.biorxiv.org/content/10.1101/2022.03.25.485799v1

Design specifications

Specify the number of blocks, trials or experimental units per session and/or subject, and specify the length of each trial or block (if trials are blocked) and interval between trials.

Behavioral performance measures

State number and/or type of variables recorded (e.g. correct button press, response time) and what statistics were used to establish that the subjects were performing the task as expected (e.g. mean, range, and/or standard deviation across subjects).

Acquisition

Imaging type(s)

Specify: functional, structural, diffusion, perfusion.

Field strength

Specify in Tesla

Sequence & imaging parameters

Specify the pulse sequence type (gradient echo, spin echo, etc.), imaging type (EPI, spiral, etc.), field of view, matrix size, slice thickness, orientation and TE/TR/flip angle.

Area of acquisition

State whether a whole brain scan was used OR define the area of acquisition, describing how the region was determined.

Diffusion MRI ☐ Used ☒ Not used

Preprocessing

Preprocessing software

Provide detail on software version and revision number and on specific parameters (model/functions, brain extraction, segmentation, smoothing kernel size, etc.).

Normalization

If data were normalized/standardized, describe the approach(es): specify linear or non-linear and define image types used for transformation OR indicate that data were not normalized and explain rationale for lack of normalization.

Normalization template	*Describe the template used for normalization/transformation, specifying subject space or group standardized space (e.g. original Talairach, MNI305, ICBM152) OR indicate that the data were not normalized.*
Noise and artifact removal	*Describe your procedure(s) for artifact and structured noise removal, specifying motion parameters, tissue signals and physiological signals (heart rate, respiration).*
Volume censoring	*Define your software and/or method and criteria for volume censoring, and state the extent of such censoring.*

Statistical modeling & inference

Model type and settings	*Specify type (mass univariate, multivariate, RSA, predictive, etc.) and describe essential details of the model at the first and second levels (e.g. fixed, random or mixed effects; drift or auto-correlation).*
Effect(s) tested	*Define precise effect in terms of the task or stimulus conditions instead of psychological concepts and indicate whether ANOVA or factorial designs were used.*

Specify type of analysis: ☐ Whole brain ☐ ROI-based ☐ Both

Statistic type for inference (See Eklund et al. 2016)	*Specify voxel-wise or cluster-wise and report all relevant parameters for cluster-wise methods.*
Correction	*Describe the type of correction and how it is obtained for multiple comparisons (e.g. FWE, FDR, permutation or Monte Carlo).*

Models & analysis

n/a	Involved in the study
☒	☐ Functional and/or effective connectivity
☒	☐ Graph analysis
☒	☐ Multivariate modeling or predictive analysis
